# Physicochemical, Microbial, and Volatile Compound Characteristics of *Gochujang*, Fermented Red Pepper Paste, Produced by Traditional Cottage Industries

**DOI:** 10.3390/foods11030375

**Published:** 2022-01-27

**Authors:** Srinivasan Ramalingam, Ashutosh Bahuguna, SeMi Lim, Ah-Ryeong Joe, Jong-Suk Lee, So-Young Kim, Myunghee Kim

**Affiliations:** 1 Department of Food Science and Technology, Yeungnam University, Gyeongsan 38541, Korea; sribt27@gmail.com (S.R.); ashubahuguna@gmail.com (A.B.); thfvkalfpeh7@naver.com (S.L.); whdkfud12@naver.com (A.-R.J.); 2 Division of Food & Nutrition and Cook, Taegu Science University, Daegu 41453, Korea; jslee1213@ynu.ac.kr; 3 Department of Agrofood Resources, National Institute of Agricultural Sciences, Rural Development Administration, Wanju 55365, Korea; foodksy@korea.kr

**Keywords:** alcohol, *gochujang*, *Bacillus cereus*, free amino nitrogen, *Zygosaccharomyces rouxii*

## Abstract

*Gochujang*, fermented red pepper paste, is a grain-based Korean traditional food. The quality of *gochujang* produced by cottage industries is not well-documented. Thus, the present study aimed to analyze the quality of *gochujang* from 35 traditional cottage industries for physicochemical and microbial characteristics, along with volatile compound contents. In addition to microbial characteristics, salinity, pH, free amino nitrogen, and alcohol content were evaluated. Ethanol was detected as the predominant alcohol and 57% of tested *gochujang* products harbored >1% of total alcohol content, which was above the recommended level for halal products. *Gochujang* products contained hexadecanoic and linoleic acids predominantly and several volatile compounds belonging to the classes of alcohols, aldehydes, alkanes, nitrogen-containing compounds, and terpenes. A wide range of aerobic mesophilic bacteria (2.79–8.73 log CFU/g) and yeast counts (1.56–7.15 log CFU/g) was observed. Five distinct yeast species were identified, including *Zygosaccharomyces rouxii*. Eight *gochujang* products were found to be contaminated with *Bacillus cereus* (>4 log CFU/g). This study suggests that there is a need to limit *B. cereus* contamination in cottage industry products and reduce alcohol content to comply with halal food guidelines.

## 1. Introduction

*Gochujang* (fermented red pepper paste) is one of the most important grain-based traditional Korean fermented foods and is generally used as a sauce in Korean cuisines and as a seasoning in spicy foods. *Gochujang* has a distinguished flavor and savory taste [1]. In 2017, the total domestic and international retail market revenue of *gochujang* accounted for approximately USD 149.55 million and USD 31.98 million, respectively. *Gochujang* products are exported to several countries, including the US, China, Japan, and Middle Eastern countries [2]. Owing to the high amount of saccharified grain starch (from rice, wheat, or barley), and powdered red hot pepper (*Capsicum annuum* L.), *gochujang* is a red and thick paste. Furthermore, significant amounts of salt, powdered *meju*, and potable water are used in the preparation of *gochujang*. *Meju* is a naturally fermented soybean, which acts as the source of microorganisms (starter culture) in the fermentation of *gochujang*. The mixture of these ingredients starts the fermentation and aging processes [3]. Two major types of *gochujang* are available in the market: a modern large-scale industrial *gochujang* and traditional homemade *gochujang* [4,5]. The modern large-scale industrial *gochujang* is produced in a quality-controlled environment with the use of specific starter cultures (*Aspergillus* and *Bacillus* species) in a short period of fermentation [6].

The preparation of homemade and cottage industrial *gochujang* relies on traditional fermentation techniques using simple equipment. The traditional homemade *gochujang* is produced using an extensive fermentation process with natural microorganisms. The process includes saccharification via heating of glutinous rice and malt, followed by the addition of *meju*, red pepper powder, and salt, depending on the desired characteristics of taste and flavor, and, finally, a fermentation stage, which can last from 1 to 2 years [7]. The microbial composition of *meju* can affect the quality of *gochujang* [7]. The traditional *gochujang* fermentation is influenced by several elements, including local microorganisms such as the *meju* microflora, and surrounding environmental factors such as weather conditions [4,5,8]. Thus, the *gochujang* cottage industry in different provinces generates products with diversified nutritional values and organoleptic properties [9]. Raw ingredients, process methods, microorganisms involved in the fermentation, and duration of the fermentation significantly influence the organoleptic properties of *gochujang*, including its aroma, taste, and texture [6,8].

To make appropriate choices and optimize the production of traditional *gochujang*, it is essential to investigate the physicochemical and microbial characteristics. Although various laboratory and homemade unbranded *gochujang* products have been previously examined [9,10], these studies have not focused on the physicochemical and microbial properties and volatile compound characteristics of indigenous-branded, traditional cottage industrial *gochujang* products. Generally, because of the use of traditional processing technologies adopted by cottage industries, the interbatch quality of *gochujang* remains unvaried [10]. Hence, consumers are highly interested in indigenous branded traditional *gochujang* products owing to the consistently outstanding quality. These traditional cottage industries are operated with minimal capital, and thus lack a quality control department for the analyses of *gochujang* products. Moreover, the physicochemical and microbial features of these products are not monitored by any food and health organization and, therefore, not publicly available.

A previous study detected a significant amount of different alcohol types, particularly ethanol, in *gochujang* during the fermentation process [11]. In addition to the basic ethanol content, some companies supplement the product with a considerable amount of ethanol during the packaging phase to prevent microbial activity. The *gochujang* products containing more than 1% ethanol are prohibited for trade in Muslim countries (halal markets). Moreover, the risk of contamination of traditional cottage industry *gochujang* products with food pathogens, particularly *Bacillus cereus*, remains unexplored. The quality of *gochujang* products produced by cottage industries has not been sufficiently examined. This study aimed to determine the physicochemical and microbial properties, alcohol content, and volatile compounds of *gochujang* products collected from nationwide cottage industries, and to categorize such products based on the findings of biostatistical analyses.

## 2. Materials and Methods

### 2.1. Chemicals

All chemicals used were of analytical grade. Potassium chromate, 0.1 N sodium hydroxide, silver nitrate, methyl alcohol, ethyl alcohol, and sodium chloride were obtained from Duksan Pure Chemicals (Ansan, Gyeonggi-do, Korea). Sodium hydroxide, sodium hydrogen carbonate, and ammonium hydroxide were purchased from Junsei Chemicals (Tokyo, Japan). Formalin solution, standard methanol, ethanol, pentanol, propanol, and butanol were purchased from Sigma-Aldrich (St. Louis, MO, USA). Plate count agar, nutrient agar, potato dextrose agar (PDA), and potato dextrose broth were purchased from Difco (Becton, Dickinson and Company, Sparks, MD, USA). Mannitol egg yolk polymyxin agar (MYP), egg yolk emulsion, and polymyxin B supplement were purchased from Oxoid LTD (Basingstoke, Hampshire, UK). 3M Yeast and Mold Petrifilm was purchased from 3M Health Care (St. Paul, MN, USA). API 50CHB and API 20E were obtained from bioMerieux (Marcy I’Etoile, France).

#### Instruments and Apparatus

A pH meter (Orion Star A211, Thermo Fisher Scientific, Beverly, MA, USA) and Konica Minolta Chromameter, equipped with a CR-400 model chromameter measuring head and DP-400 model data processor, were used to measure the pH and color values, respectively. GC-MS-QP2010 SE (Shimadzu Co., Kyoto, Japan) gas chromatography–mass selective detection (GC-MSD) system with SH-Stabilwax column (30 m × 0.32 mm × 0.25 µm) and Agilent 7890B and 5977B GC-MS system (Agilent, Santa Clara, CA, USA), which includes an Agilent DB-WAX 122-7062 column (60 m × 250 µm × 0.25 µm), were used for the detection of volatile compounds and alcohol content, respectively. Plastic Petri plates (SPL Life Sciences, Pocheon, Gyeonggi, Korea) were used for the microbiological analysis. Internal transcribed spacer (ITS) sequencing of isolated microbes was conducted using the ABI PRISM 3730XL DNA analyzer (Applied Biosystems, Foster City, CA, USA).

### 2.2. Sample Collection

A total of 35 *gochujang* products were purchased from various cottage industries located in different provinces of the Republic of Korea, as previously reported [1]. The major ingredients of *gochujang* products include red pepper powder, glutinous rice powder, powdered soybeans, grain syrup, malt, salt, and water. Detailed ingredients of the purchased *gochujang* products were also previously reported [1].

### 2.3. Physicochemical Characteristics

#### 2.3.1. Determination of pH, Salinity, Color Values, and Free Amino Nitrogen

pH values of *gochujang* products were analyzed according to the protocol of Ramalingam et al. [12]. The salinity of *gochujang* was determined using the Korea Food and Drug Administration method [13]. Color values of *gochujang* were obtained using a chromameter. The tristimulus color analyzer was calibrated to a reference (white porcelain plate) prior to the experiment [14]. The total free amino nitrogen contents of the *gochujang* samples were determined using the titration method as described by the Korea Food and Drug Administration [15] and Cho et al. [16].

#### 2.3.2. Determination of Total Alcohol Content

The alcohol content profiles of *gochujang* products were investigated using gas chromatography–mass spectrometry (GC-MS), according to the method described by Lee et al. [17] and Gil et al. [18]. Briefly, 0.5 g of a sample was mixed with 9.5 mL of dimethyl sulfoxide and stirred at 100 rpm at 40 °C for 1 h in a 20 mL closed container. The reaction solution settled before the supernatant was filtered using the Whatman syringe filter. Subsequently, the supernatant was used for the GC-MS analysis via a GC-MSD system. A temperature of 160 °C was maintained in the GC injector, and 20 µL of the sample was injected with a split ratio of 40:1. The oven temperature was programmed to start at 40 °C for 5 min, and increase 10 °C/min up to 240 °C, and then stop at (isothermal) 240 °C for 9 min. Mass spectrum analysis (70 eV, ion-source temperature 200 °C) was performed at 0.5 s scan intervals. Standard methanol, ethanol, pentanol, butanol, and propanol solutions (0.2%) were used to estimate each alcohol concentration in the *gochujang* samples.

### 2.4. Determination of Volatile Compounds

The volatile compound profiles of *gochujang* products were investigated using a solid-phase microextraction (SPME) method, followed by GC-MS [12]. Approximately 5 g of sample was heated to 70 °C for 20 min in a closed 20 mL container. A carbowax/divinylbenzene polydimethylsiloxane SPME fiber assembly was allowed to absorb the volatile compounds within the samples for 30 min at 70 °C. Temperatures of 250 °C and 230 °C were maintained in the GC injector and MS source, respectively. A split ratio of 20:1 was used to inject the SPME fiber at a purge flow rate of 3 mL/min (with a total flow rate of 24 mL/min) at 18.5 psi. The oven temperature was programmed to start at 40 °C for 2 min, and increase at a rate of 2 °C/min up to 220 °C and 10 °C/min up to 240 °C, and then stop at 240 °C for 10 min. Mass spectrum analysis (70 eV, ion-source temperature 230 °C) was performed at 0.5 s scan intervals. Mass spectra of the unknown compounds of samples were interpreted using the data available in the National Institute of Standards and Technology MS library [19]. The molecular weights, names, and structures of volatile compounds in the samples were determined.

### 2.5. Microbial Profile

The standard methods of the Association of Official Analytical Chemists [20] were adopted to analyze the total number of aerobic mesophilic bacteria and *B*. *cereus* in *gochujang*. 3M Petrifilm, plate count agar, and MYP culturing medium were used according to the manufacturer’s protocol to estimate the total yeast and mold (yeast/mold) [21], aerobic mesophilic bacteria, and *B*. *cereus* counts, respectively. API 50CHB and API 20E kits were used to identify *B*. *cereus* using the manufacturer’s protocol. PDA was used to isolate yeast/mold. The isolated yeast/mold from *gochujang* was subjected to ITS sequencing analysis [12]. The analyzed sequences were aligned with the help of the sequence alignment editor software BioEdit (version 7.0.4). The data on ITS sequences of the isolated microorganisms were documented in the NCBI GenBank database using the BLAST program. Phylogenetic analysis was performed for the isolated microorganisms using the neighbor-joining method [12].

### 2.6. Statistical Analysis

All the experiments were performed at least in triplicate, and the values were presented as the mean ± standard deviation. Statistical analyses were performed using the SPSS software 23 (IBM, Chicago, IL, USA). One-way analysis of variance in a completely randomized design and Duncan’s multiple range comparison tests were used to explore the significant differences between the samples with a 95% confidence interval at *p* < 0.05. The multivariate exploratory techniques of principal component analysis (PCA) were conducted to categorize the *gochujang* samples based on their pH, lightness, redness, yellowness, amino nitrogen content, aerobic mesophilic bacteria count, yeast/mold count, and major volatile compound profile using the XLSTAT package on Microsoft Office Excel 2016 version [1].

## 3. Results and Discussion

### 3.1. Physicochemical Analysis of Gochujang Products

#### 3.1.1. pH

Optimal pH is one of the prerequisite physicochemical parameters of fermented foods and is the main factor influencing the occurrence of several biochemical activities [12]. All selected *gochujang* products exhibited acidic pH between the ranges of 3.57 ± 0.01–4.98 ± 0.01 (Table 1). The mean pH value of *gochujang* was 4.44 ± 0.35. Based on the pH values, all the *gochujang* products were grouped into two categories: samples with pH higher than 4.6 (low-acidic food), and samples with pH below 4.6 (acidic food) (USFDA, Code of Federal Regulations) [22]. A total of 40% of the *gochujang* products (*n* = 14) showed a pH higher than 4.6 (in the range of low-acid food), whereas 60% (*n* = 21) presented pH values below 4.6 (acidic food). The variation in pH between the different *gochujang* products is probably due to the origin of different basic raw materials and the contribution of different microorganisms. A previous report showed a range of low-acidic pH (4.59 ± 0.36–4.79 ± 0.15) measured in 80 different homemade *gochujang* products [23]. However, Lee et al. [23] did not report pH values below 4.0 for any sample. The present investigation detected a slightly acidic pH for some samples, similar to that reported by Kim et al. [4] in several laboratory-made *gochujang*–*meju* samples. In general, the initial pH values of the *gochujang* products ranged from 5.5–6. These values are then reduced to the level of either low-acidic food or acidic food pH values during the fermentation process [24,25]. The decrease in the pH value is dependent on the fermentation time [26], fermenting microbes [24], environmental factors [27], and raw materials [25]. The mean pH value (4.44 ± 0.35) measured in this investigation was similar to that previously reported for other *gochujang* products [23,24,25,26,27,28].

#### 3.1.2. Salinity

The salinity of the tested *gochujang* products was between 3.44 ± 0.00% and 12.68 ± 0.33%, and the mean salinity value was 6.66 ± 2.18% (Table 1). The *gochujang* products were categorized based on salinity in three broad groups, group I (salinity <5%), group II (salinity, 5–10%), and group III (salinity >10%). Most of the samples (68.57%) were placed in group II, followed by groups I (20%) and III (11.43%). This was due to the initial amount of salt added during the manufacturing phase of the *gochujang* products at the cottage industry. In the present study, all the tested *gochujang* products were prepared using salt supplements between 5% and 12%, which further impacted the salinity of the final product [1]. During the *gochujang* fermentation process, salinity increase was also detected by Beak et al. [27], whereas a decrease in salinity was reported by Ryu et al. [24]. The water content of the raw materials and external environment humidity showed a significant influence on the salt concentration of *gochujang* products [4]. The salinity of *gochujang* products reported in previous reports [23,24,27] was consistent with mean salinity observed in the present study (6.66 ± 2.18%). Moreover, Lee et al. [23] reported that none of the *gochujang* samples had salinity below 5% or above 10%.

#### 3.1.3. Free Amino Nitrogen Content

In the 35 *gochujang* products, free amino nitrogen content presented mean values of 60.33 ± 32.51 mg/100 g (Table 1). The free amino nitrogen content in all the samples ranged from 18.69 ± 0.00 mg/100 g to 168.17 ± 16.18 mg/100 g. *Gochujang* products were grouped into three categories based on the free amino nitrogen content, including group I (free amino nitrogen, 0–50 mg/100 g), group II (50–100 mg/100 g), and group III (100–200 mg/100 g). A total of 45.7% of the *gochujang* products were assigned to groups I and II, whereas only 8.6% of samples were placed in group III. It has been reported that the fermentation process increases the amino nitrogen in the *gochujang* products [24,27,28,29,30]. Similarly, the prevalence of *Bacillus* spp. and *Zygosaccharomyces* spp. has a significant correlation with an amino-type nitrogen concentration of *gochujang* products [24]. The difference in free amino nitrogen content in the tested *gochujang* products was due to the distinct initial raw material used (particularly protein-rich matter), fermentation period, and the microorganisms involved in the fermentation of *gochujang* [24,27]. Because the 35 *gochujang* products were prepared with different raw materials, including powdered soybean (a major protein substrate) [12], they had diverse free amino nitrogen content. Similarly, a previous study reported the difference in the free amino nitrogen content in various industrial *gochujang* products [31]. Accordingly, Kim et al. [4] reported the variation in free amino nitrogen content in homemade *gochujang* products prepared with four different types of *meju*.

#### 3.1.4. Color Values

The surface color of all the *gochujang* products was measured using a chromameter and are presented in Table 1. Color is an essential food quality for consumer acceptability. The color of the fermented food is highly dependent on the raw material used and the composition of the final product [12]. The mean values of lightness (L*), redness (a*), and yellowness (b*), of *gochujang* products, were 28.11 ± 2.04, 10.04 ± 3.15, and 7.59 ± 1.53, respectively. The most influential factor responsible for the redness of the products is the red pepper powder. In the present study, Go-30 displayed the highest value for redness (17.72 ± 0.13) owing to the high percentage of red pepper (34%) during preparation, whereas Go-20 had the lowest value (4.96 ± 0.12) due to the limited amount (19%) of red pepper. A previous report revealed that the progression in the fermentation process increased the a* and L* values of *gochujang* products, whereas b* values were decreased [24]. In another investigation, a* and L* values decreased, and no significant changes were observed in the b* values during the 1-year fermentation of *gochujang* products evaluated [27]. In addition to the raw material, the variation in the color values for different *gochujang* products is associated with the microbial composition, which metabolizes the complex biomolecules and converts them into simple molecules responsible for a unique taste, aroma, and color. The present results, including the mean color values of the *gochujang* products, were consistent with those reported in previous reports [24,27,28].

#### 3.1.5. Alcohol Content

**Figure 1:**
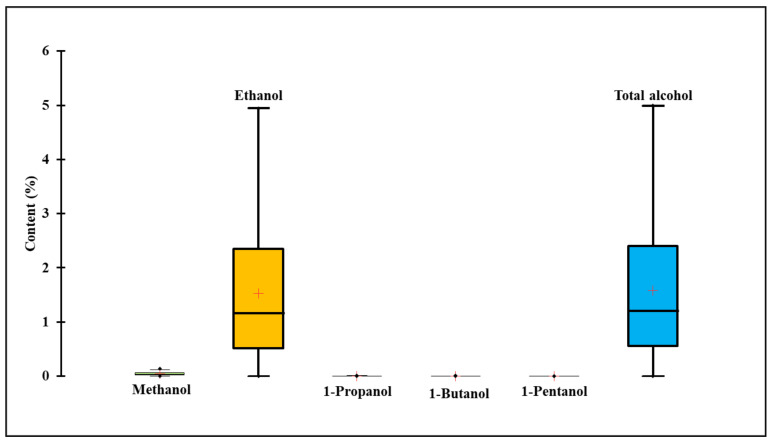
The content of various alcohols in 35 *gochujang* products from the traditional cottage industry.

Alcohols, particularly ethanol, are important volatile components of fermented foods, responsible for imparting a unique flavor and aroma [32]. A wide range (0–4.99%) was noticed in the alcohol content and proportions of the tested *gochujang* products. The mean total alcohol content was 1.58 ± 1.28% (Figure 1 and Supplementary Table S1). Among the tested alcohols (methanol, propanol, butanol, and pentanol), ethanol content was the highest, ranging from 0 to 4.9%. Therefore, ethanol represented the single major contributor to the total alcohol content of *gochujang* products (Figure 1 and Supplementary Table S1). The mean ethanol content of the *gochujang* products was 1.53 ± 1.23%, whereas the mean methanol content was 0.004 ± 0.005% (Figure 1).

None of the products showed an excessively high amount of propanol, butanol, and pentanol, and these alcohols were detected in the range from not detected to 0.016%. *Gochujang* products were grouped into two categories based on the total alcohol content, including group I (0–1%) and group II (>1%) [33]. Approximately 42.85% of the samples (*n* = 15) were placed in group I, whereas the rest of the *gochujang* products (*n* = 20) were assigned in group II. The alcohol content in 57.15% of the tested *gochujang* products (*n* = 20) was higher than the recommended amount (1%) for halal foods [32]. Moreover, six *gochujang* products contained more than 3% of ethanol. In these cases, the extra ethanol was added by the manufacturing companies during the packaging of *gochujang* products to prevent spoilage and microbial activity. Furthermore, the basic ethanol content in *gochujang* products is linked with types and populations of fungi participating in the fermentation [34]. In particular, *Zygosaccharomyces* spp. and *Saccharomyces* spp. yeast isolated from *gochujang* products produced 1.6–3.2% of the ethanol [17], thus contributing to the higher basic level of ethanol in *gochujang* products. Previous data generated using the electronic nose analysis of 25 traditional *gochujang* products revealed the presence of 0.14–2.7% of ethanol in *gochujang* products, and 44% of the products (*n* = 11) contained more than 1% of ethanol content, thus supporting the present findings [35]. An abnormally high amount of alcohol content in *gochujang* products leads to alteration in taste and may cause spoilage. Moreover, ethanol content higher than 1% in food restricts their consumption in Muslim countries due to halal requirements [33].

### 3.2. Volatile Compounds

GC–MS analysis results revealed the presence of various volatile compounds in all tested *gochujang* products. The total number of compounds identified in the *gochujang* products ranged between 53 and 104 (Supplementary Table S2). The compound names, retention times, and percentage peak area for all *gochujang* products are listed in Supplementary Table S2. The predominant compound was identified as 2,3,5,6-tetramethyl pyrazine with a peak area of 54.31% and retention time of 40.054 min in Go-19, followed by ethanol with a peak area of 49.26% and retention time of 9.138 min in Go-7.

Ethanol was found in all tested *gochujang* products, with a peak area percentage range of 1.17–49.26%. Among the 35 *gochujang* products, 17 showed ethanol as the predominant component, depicting a percentage range of the highest peak area between 7.54–49.26%. Linoleic acid ethyl ester and 2,3,5,6-tetramethyl pyrazine were detected as the predominant components in 11 *gochujang* products with a peak area range of 7.92–31.19%, and to a lesser extent, in four other *gochujang* products (Go-14, Go-16, Go-19, and Go-30) with peak area range of 2.7–54.31%. Hexadecanoic acid, an ethyl/methyl ester, was detected in all *gochujang* products and was either the second or third most abundant compound in 26 *gochujang* products with a peak area range of 6.49–25.09%. Compounds detected in the *gochujang* products included low quantities of acids, alcohols, aldehydes, alkanes, alkenes, benzene derivatives, carboxylic acids, cyclic and bicyclic ketones, cyclosiloxanes, esters, fatty acids, furans, hydrocarbons, nitrogen-containing compounds, phenolics, pyranones, pyrazines, sulfur-containing compounds, and terpenes.

Several compounds (such as ethanol, acetic acid, benzaldehyde, benzene acetaldehyde, hexadecanoic acid, ethyl ester, hexanoic acid, hexanol, hexyl ester, linoleic acid ethyl ester, methyl salicylate, nonanoic acid, ethyl ester, octadecanoic acid, ethyl ester, 1-propanol, octanoic acid, and ethyl ester) were previously reported in *gochujang* [36,37,38,39,40,41,42], thus supporting the present results. Similar to the present investigation, previous studies reported a high amount of ethanol in the commercial, traditional, and improved *gochujang* products generated during the fermentation process via the yeast-dependent glycolytic pathway [36,37,43,44,45,46]. Most of the ester compounds detected in the *gochujang* products were ethyl esters, which render a fruity aroma to the product; fatty acid esters are produced due to the esterification of organic acids and fatty acids with the ethanol generated during the fermentation process by the yeast [37]. Several aroma-active compounds, such as acetic acid (pungent sour), 2-furanmethanol (cooked sugar), methyl salicylate (peppermint), ethanol (sweet), linalool (flower and lavender), hexanal (grass, tallow, and fat), benzaldehyde (almond and burned sugar), benzene acetaldehyde (fruity and rosy), nonanal (soapy), tetramethylpyrazine (cocoa, mocha, and milk coffee), acetic acid (sour) and ethyl hexanoate (apple peel, and fruit) were detected in the tested *gochujang* products [37,47]. Diversified classes of compounds with different peak areas were detected in each *gochujang* product due to the difference in raw materials, method of raw material processing, microbial diversity during the fermentation process, and fermentation period and conditions.

### 3.3. Microbial Profile Analysis

#### 3.3.1. Aerobic Mesophilic Bacteria and Yeast/Mold

The tested *gochujang* products harbored aerobic mesophilic bacteria in the range of 2.79 ± 0.10 to 8.73 ± 0.30 log CFU/g (Table 2). The mean value of aerobic mesophilic bacteria present in the *gochujang* products was 6.98 ± 1.42 log CFU/g. In addition to the aerobic mesophilic bacteria, the active presence of fungi and yeast in *gochujang* was previously reported [7]. The yeast/mold count in all products ranged from 1.56 ± 0.06 to 7.15 ± 0.02 log CFU/g (Table 2). The mean value of *gochujang* products’ yeast/mold population was 4.47 ± 1.47 log CFU/g (Table 2). In general, during the *gochujang* fermentation phase, aerobic mesophilic bacterial populations increased from approximately 5 log CFU/g to 8 log CFU/g, and yeast/mold counts decreased [24,26,28]. The findings from the present study are in agreement with those from previous reports that showed similar bacterial and yeast count in different *gochujang* products [25,26,48]. The microbial population in the products highly depended on external environmental factors, physicochemical and microbial profiles of raw materials, and the *meju* used as a starter culture [24,27]. Even though changes occurred in the microbial composition, the total count of aerobic bacteria was nearly constant after 3 months of *gochujang* fermentation [27]. Both bacteria and yeast/mold play important roles in the final features (taste, color, and aroma) of the fermented *gochujang* products [24,27,37,38].

Several reasons can be identified for the variation in yeast/mold count among the *gochujang* products, among which the selection of *meju* may be the most critical. The present results are in accordance with several published reports that indicated the presence of several microorganisms in *gochujang* [7,48,49]. In *gochujang*, various bacterial species have been identified and extensively studied [7,37]. However, studies regarding the presence of yeast in *gochujang* are limited [7]. Thus, the present investigation focused on the isolation and identification of yeast from all *gochujang* products. More than 100 yeast colonies were isolated from 35 *gochujang* products. After microscopic examination and evaluation of colony characteristics, five distinct yeast colonies were analyzed using ITS sequencing and comparative phylogenetic analysis (Supplementary Figures S1–S5). These colonies were identified as *Zygosaccharomyces rouxii*, *Starmerella lactis-condensi*, *Wikerhamomyces subpelliculosus*, *Pichia membranifaciens*, and *Cladosporium welwitschiicola* (Table 2). To the best of our knowledge, *P*. *membranifaciens*, *C*. *welwitschiicola*, and *W*. *subpelliculosus* were reported in the traditional *gochujang* products for the first time. *Zygosaccharomyces rouxii* was detected as a predominant yeast in 82.85% of *gochujang* products (*n* = 29). It produces several aromatic secondary metabolites during fermentation, such as esters, aldehydes, and ketones, with leavening properties [50] that improve the quality of *gochujang* products [24,51,52]. *Z*. *rouxii* is the main yeast species found in the traditional *gochujang* products, whereas *Candida* and *Cryptococcus* species were dominant in the commercial *gochujang* products, supporting the present results [7,48,49]. Phylogenetic analysis displayed a minor variation between the identified *Z*. *rouxii* strains. The high occurrence of *Z*. *rouxii* in *gochujang* products led to its high isolation frequency in the present study (Table 2), which was in accordance with the data from Jang et al. [7].

#### 3.3.2. Detection of *B. cereus* in *Gochujang* Products

The presence of pathogenic bacteria in food represents a major concern for food safety. *Escherichia coli*, *B. cereus*, *Salmonella* species, and *Staphylococcus aureus* are common foodborne pathogens responsible for significant health and economic losses. Although the acidic pH of *gochujang* products acts as a barrier for most of the pathogenic microbes, *B*. *cereus* can proliferate in *gochujang* [8]. In the present study, the presence of *B*. *cereus* was observed in eight *gochujang* products (22.85%) at a level higher than the safety limit (4 log CFU/g) recommended by the Korean Food and Drug Administration [53] (Table 3). Yim et al. [54] measured *B*. *cereus* counts below 4 log CFU/g in all the tested commercial *gochujang* products. Kim et al. [10] reported the presence of *B*. *cereus* in nine industrial and 23 homemade *gochujang* samples and revealed that three homemade *gochujang* samples contained *B*. *cereus* levels higher than the safety limit. In general, the *B*. *cereus* counts increase during the *gochujang* fermentation process [8,27]. The source of *B*. *cereus* in *gochujang* may include contaminated raw materials and cross-contamination during the fermentation process. In summary, the present investigation, supported by several other studies [8,10,52], indicated that although present in *gochujang* products, the *B*. *cereus* count in most products was within the safety limit, suggesting that appropriate sterilization measures were adopted during the preparation process. However, a few *gochujang* products showed higher *B cereus* counts, thus leading to concerns regarding *B*. *cereus* contamination and the need for necessary preventive measures against such contamination.

### 3.4. Principal Component Analysis and Hierarchical Clustering of Gochujang Products

**Figure 2:**
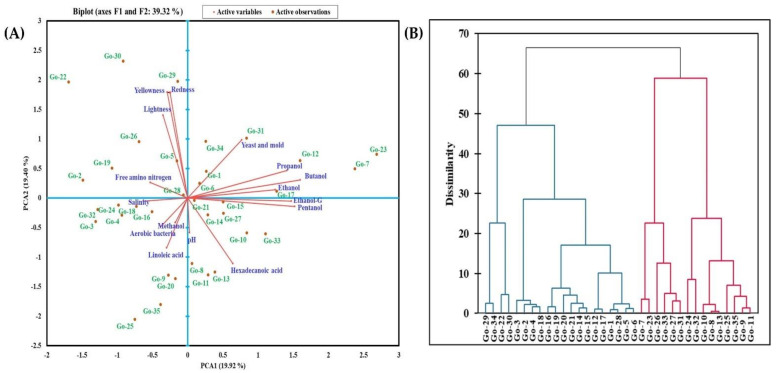
Principal component analysis (**A**) and agglomerative hierarchical clustering analysis (**B**) of 35 different *gochujang* products from the traditional cottage industry.

The PCA and agglomerative hierarchical clustering analysis were performed based on the physicochemical characteristics, microbial count, alcohol content, and the distribution of major volatile components of different *gochujang* products (Figure 2). PC1 grouped Go-1, Go-6, Go-7, Go-12, Go-17, Go-23, Go-31, and Go-34, in the positive plane from the other samples (Figure 2A). The *gochujang* samples located in the positive values of PCA1 were influenced by yeast population and alcohol content. The PC2 showed the variance and grouped Go-2, Go-5, Go-19, Go-22, Go-26, Go-28, Go-29, and Go-30 (in positive values) (Figure 2A). PCA separated and grouped the different clusters of 35 *gochujang* samples based on their pH, salinity, free amino nitrogen, lightness, yellowness, redness, aerobic bacterial count, yeast and mold count, methanol, ethanol, propanol, pentanol, and butanol content, and major components from GC-MS analysis (ethanol, linoleic acid, and hexadecanoic acid). The *gochujang* products grouped in the positive region of PCA2 were influenced by free amino nitrogen content and color values. The *gochujang* samples in the negative plane of PCA1 and 2 varied from other samples owing to the differences in salinity, aerobic bacterial count, methanol content, and linoleic acid composition (one of the major components detected in GC-MS analysis). The pH and hexadecanoic acid content displayed significant variance in *gochujang* products (Go-8, Go-10, Go-11, Go-13, Go-14, Go-15, Go-21, Go-27, and Go-33) (Figure 2A). Agglomerative hierarchical clustering analysis revealed dissimilarities between the *gochujang* products in two key groups (Figure 2B). The group I consisted of five closely-related clusters with 20 *gochujang* samples (cluster 1 = Go-29, Go-34, Go-22, and Go-30; cluster 2 = Go-3, Go-2, Go-4, and Go-18; cluster 3 = Go-16, Go-19, Go-20 Go-21, Go-14, and Go-15; cluster 4 = Go-12, and Go-17; and cluster 5 = Go-1, Go-28, Go-5, and Go-6) (Figure 2B). Group II also comprised five clusters with 15 *gochujang* products (cluster 1 = Go-7 and Go-23; cluster 2 = Go-26, Go-33, Go-27, and Go-31; cluster 3 = Go-24 and Go-32; cluster 4 = Go-10, Go-8, and Go-13; and cluster 5 = Go-25, Go-35, Go-9, and Go-11) (Figure 2B). The *gochujang* products within these 10 clusters were closely related in terms of tested parameters. To the best of our knowledge, no study has categorized *gochujang* products based on their physicochemical and microbial features using multivariate PCA. Only a limited number of previous studies have employed PCA to represent the profiling of microbes and biogenic amines in *gochujang* products [1,8,27,49].

## 4. Conclusions

Diversified physiochemical and microbial profiles were detected in *gochujang* products collected from different provinces of the Republic of Korea. This study highlighted the presence of a variety of alcohols with a predominance of ethanol in the gochujang products. A few samples had ethanol content higher than the recommended limit for halal foods. Similarly, 22% of *gochujang* products were contaminated with *B. cereus.* The study demonstrated the variations in physicochemical, microbiological, and volatile compound characteristics of *gochujang* products, which may be due to the influence of raw material and fermentation conditions. The variation in the microbial profile also influenced physicochemical constituents and volatile compounds of the *gochujang* products. Nonetheless, an exact correlation needs to be established in future studies. The outcome of the study indicates that most of the *gochujang* products were free from toxicogenic microorganisms, though a few *gochujang* products had high ethanol content and *B*. cereus contamination, which needs to be addressed to satisfy the guidelines of food safety and marketability. This study also recommends regular analysis of the *gochujang* products prepared by the cottage industry to ensure their safety toward consumers.

## Figures and Tables

**Table 1:** The pH, salinity, color values, and free amino nitrogen content of *gochujang* products.

Product Code	pH ^#^	Salinity (%) ^#^	Color Values ^#^			Free Amino Nitrogen (mg/100 g) ^#^
			Lightness (L*)	Redness (a*)	Yellowness (b*)	
Go-1	4.96 ± 0.01 ^b^	5.01 ± 0.30 ^pq^	29.70 ± 0.58 ^bcdefg^	13.36 ± 0.63 ^d^	8.74 ± 0.13 ^cdef^	28.03 ± 8.09 ^kl^
Go-2	4.49 ± 0.01 ^n^	10.59 ± 0.32 ^c^	28.26 ± 0.19 ^bcdefgh^	12.24 ± 0.10 ^e^	8.92 ± 0.07 ^cd^	65.40 ± 8.09 ^defgh^
Go-3	4.78 ± 0.01 ^d^	7.74 ± 0.00 ^fg^	26.98 ± 0.59 ^bcdefgh^	10.16 ± 0.16 ^hi^	7.09 ± 0.13 ^jk^	74.74 ± 14.01 ^cdefg^
Go-4	4.65 ± 0.01 ^i^	8.16 ± 0.00 ^ef^	27.25 ± 0.29 ^bcdefgh^	10.12 ± 0.13 ^hi^	7.95 ± 0.08 ^gh^	46.71 ± 14.01 ^hijk^
Go-5	4.74 ± 0.01 ^ef^	4.81 ± 0.27 ^qr^	28.22 ± 0.31 ^bcdefgh^	12.03 ± 0.34 ^ef^	8.93 ± 0.11 ^cd^	74.74 ± 14.01 ^cdefg^
Go-6	4.74 ± 0.01 ^f^	5.22 ± 0.26 ^opq^	29.21 ± 0.87 ^bcdefg^	11.35 ± 0.69 ^g^	8.86 ± 0.30 ^cde^	51.38 ± 8.09 ^ghijk^
Go-7	4.12 ± 0.02 ^u^	6.64 ± 0.33 ^jk^	26.91 ± 0.51 ^cdefgh^	9.33 ± 0.59 ^jk^	7.24 ± 0.44 ^jk^	65.40 ± 16.18 ^defgh^
Go-8	4.62 ± 0.01 ^j^	4.72 ± 0.00 ^qr^	26.06 ± 1.16 ^efghi^	5.76 ± 0.39 ^n^	5.47 ± 0.30 ^opq^	130.80 ± 0.00 ^b^
Go-9	4.20 ± 0.01 ^t^	7.03 ± 0.00 ^hij^	24.53 ± 0.34 ^hi^	6.88 ± 0.10 ^m^	5.89 ± 0.01 ^no^	51.38 ± 16.18 ^ghjik^
Go-10	4.30 ± 0.03 ^p^	6.0 ± 0.00 ^klmn^	25.95 ± 0.68 ^fghi^	7.26 ± 0.07 ^m^	6.47 ± 0.06 ^lm^	93.43 ± 8.09 ^c^
Go-11	3.99 ± 0.00 ^v^	4.98 ± 0.00 ^pq^	26.01 ± 1.03 ^efghi^	5.58 ± 0.78 ^no^	5.80 ± 0.29 ^no^	37.37 ± 8.09 ^ijkl^
Go-12	4.26 ± 0.01 ^q^	5.67 ± 0.67 ^mnop^	28.05 ± 0.26 ^bcdefgh^	10.64 ± 0.16 ^h^	8.47 ± 0.04 ^def^	60.73 ± 0.00 ^efghi^
Go-13	4.40 ± 0.01 ^o^	4.81 ± 0.25 ^qr^	25.88 ± 1.21 ^fghi^	6.03 ± 0.50 ^n^	5.74 ± 0.15 ^nop^	32.70 ± 0.00 ^jkl^
Go-14	4.58 ± 0.02 ^k^	7.35 ± 0.31 ^ghi^	26.33 ± 0.34 ^defgh^	8.91 ± 0.10 ^jkl^	7.07 ± 0.01 ^jk^	42.04 ± 8.09 ^hijkl^
Go-15	3.84 ± 0.01 ^x^	5.81 ± 0.53 ^lmno^	26.72 ± 0.74 ^cdefgh^	7.47 ± 0.55 ^m^	7.03 ± 0.16 ^jk^	37.37 ± 8.09 ^ijkl^
Go-16	4.62 ± 0.01 ^j^	5.95 ± 0.78 ^klmn^	26.76 ± 0.34 ^cdefgh^	9.50 ± 0.12 ^ij^	7.36 ± 0.06 ^ij^	46.71 ± 0.00 ^hijk^
Go-17	4.84 ± 0.01 ^c^	5.14 ± 0.89 ^opq^	27.30 ± 0.68 ^bcdefgh^	10.52 ± 0.09 ^h^	8.10 ± 0.02 ^fgh^	51.38 ± 8.09 ^ghijk^
Go-18	4.26 ± 0.01 ^qr^	6.46 ± 0.50 ^jkl^	27.28 ± 0.43 ^cdefghi^	12.46 ± 0.21 ^e^	9.00 ± 0.03 ^cde^	37.37 ± 8.09 ^ijkl^
Go-19	4.69 ± 0.01 ^g^	7.54 ± 0.30 ^fgh^	28.75 ± 0.62 ^bcdefgh^	11.45 ± 0.23 ^fg^	7.88 ± 0.01 ^gh^	56.06 ± 8.09 ^fghij^
Go-20	4.67 ± 0.01 ^h^	4.20 ± 0.32 ^r^	26.09 ± 0.08 ^efghi^	4.96 ± 0.12 ^o^	4.97 ± 0.10 ^q^	42.04 ± 8.09 ^hijkl^
Go-21	4.98 ± 0.01 ^a^	4.93 ± 0.30 ^q^	27.97 ± 0.16 ^bcdefgh^	9.22 ± 0.24 ^jk^	6.77 ± 0.09 ^kl^	42.04 ± 8.09 ^hijkl^
Go-22	4.29 ± 0.02 ^p^	8.52 ± 0.50 ^de^	31.21 ± 0.57 ^ab^	16.63 ± 0.22 ^b^	11.19 ± 0.14 ^a^	168.17 ± 16.18 ^a^
Go-23	4.23 ± 0.01 ^s^	5.01 ± 0.31 ^pq^	30.28 ± 0.14 ^abcde^	12.63 ± 0.12 ^e^	8.38 ± 0.07 ^efg^	65.4 ± 16.18 ^defgh^
Go-24	3.94 ± 0.01 ^w^	6.06 ± 0.33 ^klm^	30.41 ± 0.17 ^abcd^	8.77 ± 0.21 ^kl^	6.05 ± 0.24 ^mn^	126.13 ± 16.18 ^b^
Go-25	4.79 ± 0.02 ^d^	6.95 ± 0.00 ^hij^	25.67 ± 0.10 ^ghi^	6.95 ± 0.31 ^m^	5.42 ± 0.10 ^opq^	28.03 ± 8.09 ^kl^
Go-26	4.55 ± 0.01 ^l^	12.68 ± 0.33 ^a^	30.90 ± 0.07 ^abc^	14.76 ± 0.04 ^c^	10.08 ± 0.02 ^b^	79.41 ± 8.09 ^cdef^
Go-27	4.56 ± 0.01 ^l^	11.36 ± 0.57 ^b^	28.39 ± 0.03 ^bcdefgh^	8.33 ± 0.04 ^l^	7.06 ± 0.02 ^jk^	46.71 ± 14.01 ^hijk^
Go-28	4.78 ± 0.00 ^d^	6.63 ± 0.34 ^jk^	29.14 ± 0.22 ^i^	10.40 ± 0.15 ^ij^	7.98 ± 0.09 ^cde^	18.69 ± 0.00 ^l^
Go-29	3.99 ± 0.01 ^v^	5.68 ± 0.00 ^mnop^	30.09 ± 0.04 ^abcdef^	13.93 ± 0.07 ^d^	9.05 ± 0.01 ^c^	18.69 ± 0.00 ^l^
Go-30	3.57 ± 0.01 ^z^	6.82 ± 0.57 ^ij^	30.57 ± 0.35 ^abcd^	17.72 ± 0.13 ^a^	10.52 ± 0.03 ^b^	79.41 ± 21.41 ^cdef^
Go-31	4.52 ± 0.01 ^m^	5.30 ± 0.33 ^nopq^	30.85 ± 0.17 ^abc^	13.43 ± 0.17 ^d^	9.13 ± 0.03 ^c^	37.37 ± 8.09 ^ijkl^
Go-32	3.76 ± 0.01 ^y^	5.41 ± 0.33 ^mnopq^	33.90 ± 0.23 ^a^	6.82 ± 0.14 ^m^	7.04 ± 0.07 ^jk^	84.08 ± 16.18 ^cde^
Go-33	4.76 ± 0.01 ^e^	8.94 ± 0.28 ^d^	28.24 ± 0.49 ^bcdefgh^	9.20 ± 0.02 ^jk^	7.07 ± 0.02 ^jk^	65.40 ± 21.41 ^defgh^
Go-34	4.24 ± 0.00 ^rs^	3.44 ± 0.00 ^s^	28.11 ± 0.08 ^bcdefgh^	10.49 ± 0.23 ^h^	7.76 ± 0.11 ^hi^	32.70 ± 14.01 ^jkl^
Go-35	4.53 ± 0.01 ^m^	11.58 ± 0.00 ^b^	25.77 ± 0.04 ^fghi^	5.93 ± 0.06 ^n^	5.24 ± 0.03 ^pq^	93.43 ± 21.41 ^c^
Mean ± SD	4.44 ± 0.35	6.66 ± 2.18	28.11 ± 2.04	10.04 ± 3.15	7.59 ± 1.53	60.33 ± 32.51

^#^—The values are the mean of triplicates with standard deviation. Different superscript letters (a–z) within a column indicate significant differences (*p* < 0.05) between the selected *gochujang* products when subjected to Duncan’s multiple comparison test.

**Table 2:** Microbial profile of the *gochujang* products.

Product Code	Aerobic Bacteria (log CFU/g) *	Yeast and Mold (log CFU/g) *	Isolated and Identified Yeast	GenBank Accession Number
Go-1	6.64 ± 0.16 ^o^	3.89 ± 0.04 ^hi^	*Zygosaccharomyces rouxii*	OL679471
Go-2	7.20 ± 0.17 ^kl^	3.71 ± 0.02 ^i^	*Zygosaccharomyces rouxii*	OL679472
Go-3	8.29 ± 0.07 ^b^	2.67 ± 0.05 ^kl^	*Zygosaccharomyces rouxii*	OL679473
Go-4	7.97 ± 0.23 ^d^	4.92 ± 0.03 ^ef^	*Zygosaccharomyces rouxii*	OL679474
Go-5	7.75 ± 0.16 ^fg^	3.74 ± 0.10 ^hi^	*Zygosaccharomyces rouxii*	OL679475
Go-6	7.82 ± 0.08 ^ef^	3.10 ± 0.08 ^jk^	*Zygosaccharomyces rouxii*	OL679476
Go-7	6.04 ± 0.12 ^q^	5.16 ± 0.06 ^def^	*Zygosaccharomyces rouxii*	OL679477
Go-8	7.31 ± 0.15 ^h^	3.66 ± 0.09 ^i^	*Zygosaccharomyces rouxii*	OL679478
Go-9	7.19 ± 0.11 ^i^	2.69 ± 0.01 ^m^	*Zygosaccharomyces rouxii*	OL679479
Go-10	7.10 ± 0.12 ^lm^	6.22 ± 0.03 ^b^	*Zygosaccharomyces rouxii*	OL679480
Go-11	6.17 ± 0.52 ^s^	3.15 ± 0.04 ^j^	*Zygosaccharomyces rouxii*	OL679481
Go-12	7.94 ± 0.17 ^efg^	6.10 ± 0.04 ^b^	*Zygosaccharomyces rouxii*	OL679482
Go-13	7.86 ± 0.11 ^fg^	5.96 ± 0.04 ^bc^	*Starmerella lactis-condensi*	OL679483
Go-14	7.92 ± 0.22 ^g^	4.94 ± 0.12 ^ef^	*Starmerella lactis-condensi*	OL679484
Go-15	7.92 ± 0.17 ^efg^	5.84 ± 0.10 ^bc^	*Zygosaccharomyces rouxii*	OL679485
Go-16	7.93 ± 0.00 ^e^	4.68 ± 0.03 ^fg^	*Zygosaccharomyces rouxii*	OL679486
Go-17	8.42 ± 0.04 ^a^	5.30 ± 0.15 ^de^	*Zygosaccharomyces rouxii*	OL679487
Go-18	6.35 ± 0.54 ^s^	2.37 ± 0.05 ^l^	*Starmerella lactis-condensi*	OL679488
Go-19	8.12 ± 0.09 ^c^	4.04 ± 0.03 ^hi^	*Zygosaccharomyces rouxii*	OL679489
Go-20	7.01 ± 0.06 ^kl^	4.02 ± 0.10 ^hi^	*Zygosaccharomyces rouxii*	OL679490
Go-21	6.10 ± 0.17 ^r^	4.23 ± 0.02 ^gh^	*Zygosaccharomyces rouxii*	OL679491
Go-22	7.33 ± 0.20 ^jk^	4.23 ± 0.04 ^gh^	*Zygosaccharomyces rouxii*	OL679492
Go-23	6.39 ± 0.22 ^p^	5.50 ± 0.04 ^cd^	*Zygosaccharomyces rouxii*	OL679493
Go-24	8.73 ± 0.30 ^a^	6.13 ± 0.03 ^b^	*Zygosaccharomyces rouxii*	OL679494
Go-25	8.06 ± 0.17 ^d^	2.29 ± 0.02 ^l^	*Zygosaccharomyces rouxii*	OL679495
Go-26	7.86 ± 0.09 ^efg^	6.12 ± 0.03 ^b^	*Zygosaccharomyces rouxii*	OL679496
Go-27	4.65 ± 0.14 ^n^	5.31 ± 0.01 ^de^	*Zygosaccharomyces rouxii*	OL679497
Go-28	7.84 ± 0.09 ^efg^	5.06 ± 0.07 ^def^	*Zygosaccharomyces rouxii*	OL679498
Go-29	3.48 ± 0.12 ^s^	7.15 ± 0.02 ^a^	*Zygosaccharomyces rouxii*	OL679499
Go-30	7.23 ± 0.19 ^j^	4.82 ± 0.02 ^ef^	*Zygosaccharomyces rouxii*	OL679500
Go-31	7.28 ± 0.24 ^kl^	6.90 ± 0.02 ^a^	*Wikerhamomyces subpelliculosus*	OL679501
Go-32	3.55 ± 0.43 ^u^	1.56 ± 0.06 ^m^	*Cladosporium welwitschiicola*	OL679502
Go-33	7.53 ± 0.13 ^h^	2.50 ± 0.02 ^l^	*Zygosaccharomyces rouxii*	OL679503
Go-34	2.79 ± 0.10 ^t^	6.14 ± 0.03 ^b^	*Pichia membranifaciens*	OL679504
Go-35	4.97 ± 0.50 ^m^	2.37 ± 0.04 ^l^	*Wikerhamomyces subpelliculosus*	OL679505
Mean ± SD	6.98 ± 1.42	4.47 ± 1.47		

*—The values are mean of triplicates with standard deviation. Different superscript letters (a–z) within a column indicate significant differences (*p* < 0.05) between the selected *gochujang* products when subjected to Duncan’s multiple comparison test.

**Table 3:** *Bacillus cereus* count in *gochujang* products.

Product Code	*Bacillus cereus* (Log CFU/g) *
Go-13	4.26
Go-16	5.30
Go-17	4.60
Go-19	4.60
Go-22	5.90
Go-24	6.26
Go-26	6.94
Go-31	5.26

* Safe limit of *Bacillus cereus* is 4 log CFU/g (Korea Food and Drug Administration, 2010).

## Data Availability

Data is contained within the article.

## Supplementary Materials

The following supporting information can be downloaded at: https://www.mdpi.com/article/10.3390/foods11030375/s1, Figure S1: Phylogenetic tree analyses of the ITS sequences of the *Zygosaccharomyces rouxii* isolated from *gochujang* products constructed in MEGA 6 software by employing UPGMA method; Figure S2. Phylogenetic tree analyses of the ITS sequences of the *Starmerella lactis-condensi* isolated from *gochujang* products constructed in MEGA 6 software by employing UPGMA method; Figure S3. Phylogenetic tree analyses of the ITS sequences of the *Wikerhamomyces subpelliculosus* isolated from *gochujang* products constructed in MEGA 6 software by employing UPGMA method; Figure S4. Phylogenetic tree analyses of the ITS sequences of the *Pichia membranifaciens* isolated from *gochujang* products constructed in MEGA 6 software by employing UPGMA method; Figure S5. Phylogenetic tree analyses of the ITS sequences of the *Cladosporium welwitschiicola* isolated from *gochujang* products constructed in MEGA 6 software by employing UPGMA method; Table S1. Various alcohol contents in *gochujang* products; Table S2. Various volatile compounds found in *gochujang* products.
