# Supplementary material for: Physicochemical, Microbial, and Volatile Compound Characteristics of Gochujang, Fermented Red Pepper Paste, Produced by Traditional Cottage Industries
Source: Foods. 2022 Jan 27;11(3):375. doi: 10.3390/foods11030375 (PMC8834593; doi:10.3390/foods11030375)
Supplement: Supplementary file 1 [file foods-11-00375-s001.zip › foods-1530944-supplementary.pdf]

Supplementary Data

Research Article

Physicochemical, Microbial, and Volatile Compound Characteristics of *Gochujang*, Fermented Red Pepper Paste, Produced by Traditional Cottage Industries

Srinivasan Ramalingam [1,†], Ashutosh Bahuguna [1,†], SeMi Lim [1], Jong-Suk Lee [2], Ah-ryeong Joe [1], So-Young Kim [3], Myunghee Kim [1,*]

[1] Department of Food Science and Technology, Yeungnam University, Gyeongsan 38541, Korea; sribt27@gmail.com (S.R.); ashubahuguna@gmail.com (A.B.); thfvkalfpeh7@naver.com (S.L.); whdkfud12@naver.com (A.-r.J.)
[2] Division of Food & Nutrition and Cook, Taegu Science University, Daegu 41453, Korea; jslee1213@ynu.ac.kr
[3] Department of Agrofood Resources, National Institute of Agricultural Sciences, Rural Development Administration, Wanju 55365, Korea; foodksy@korea.kr
* Correspondence: foodtech@ynu.ac.kr; Tel.: +82-53-810-2958
† Both authors contributed equally to this work.

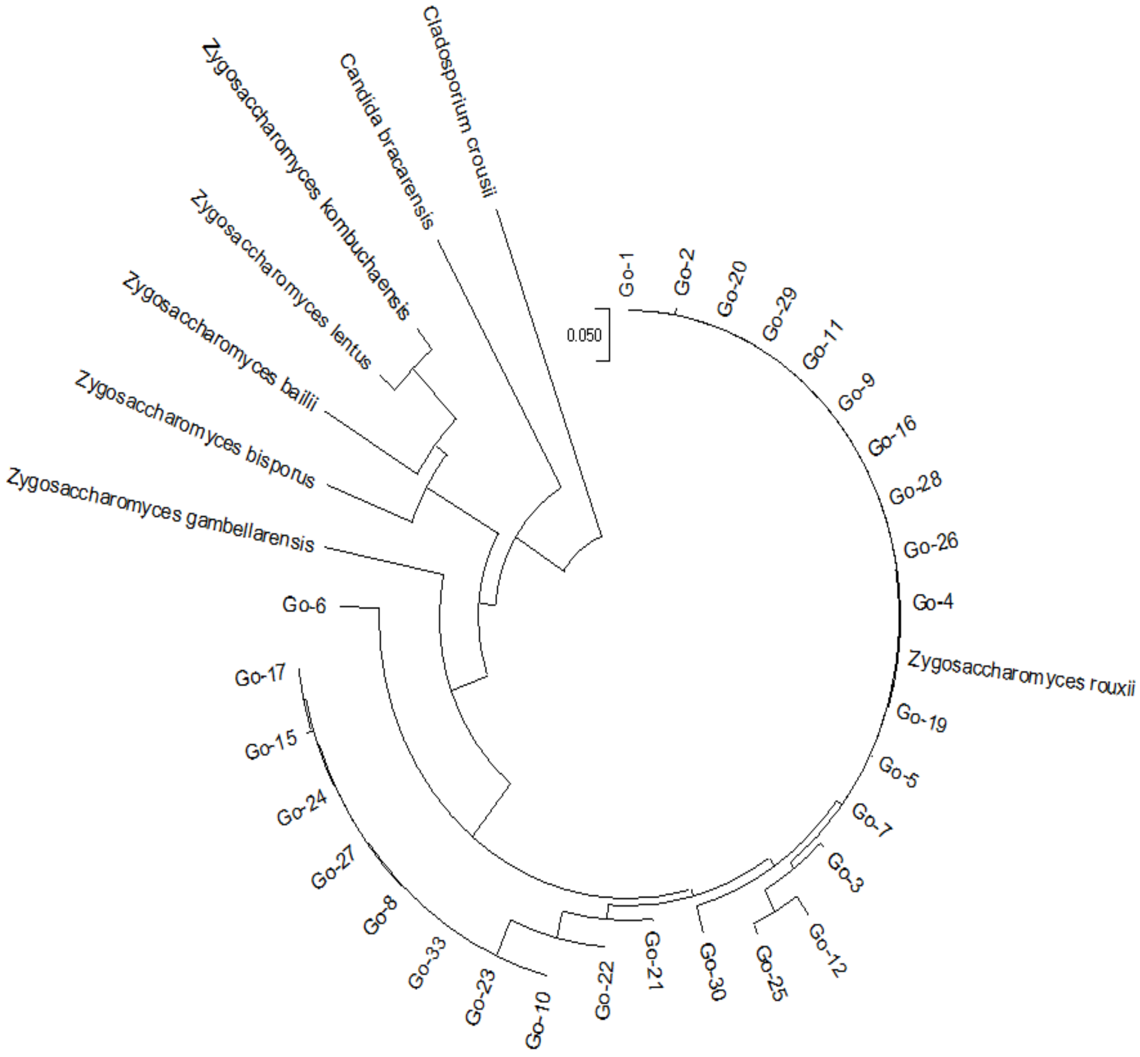

Figure S1. Phylogenetic tree analyses of the ITS sequences of the *Zygosaccharomyces rouxii* isolated from *gochujang* products constructed in MEGA 6 software by employing UPGMA method.

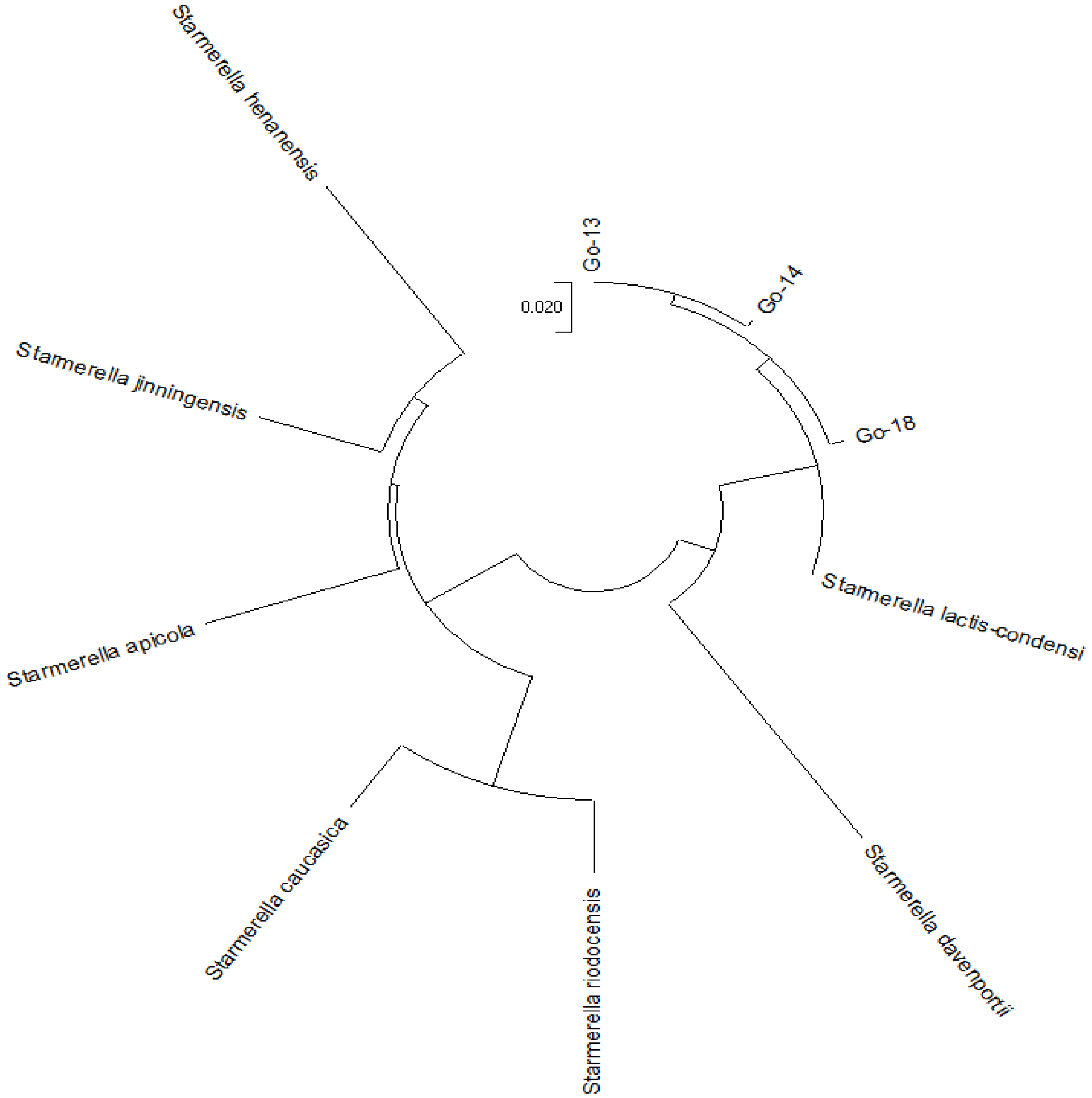

Figure S2. Phylogenetic tree analyses of the ITS sequences of the *Starmerella lactis-condensi* isolated from *gochujang* products constructed in MEGA 6 software by employing UPGMA method.

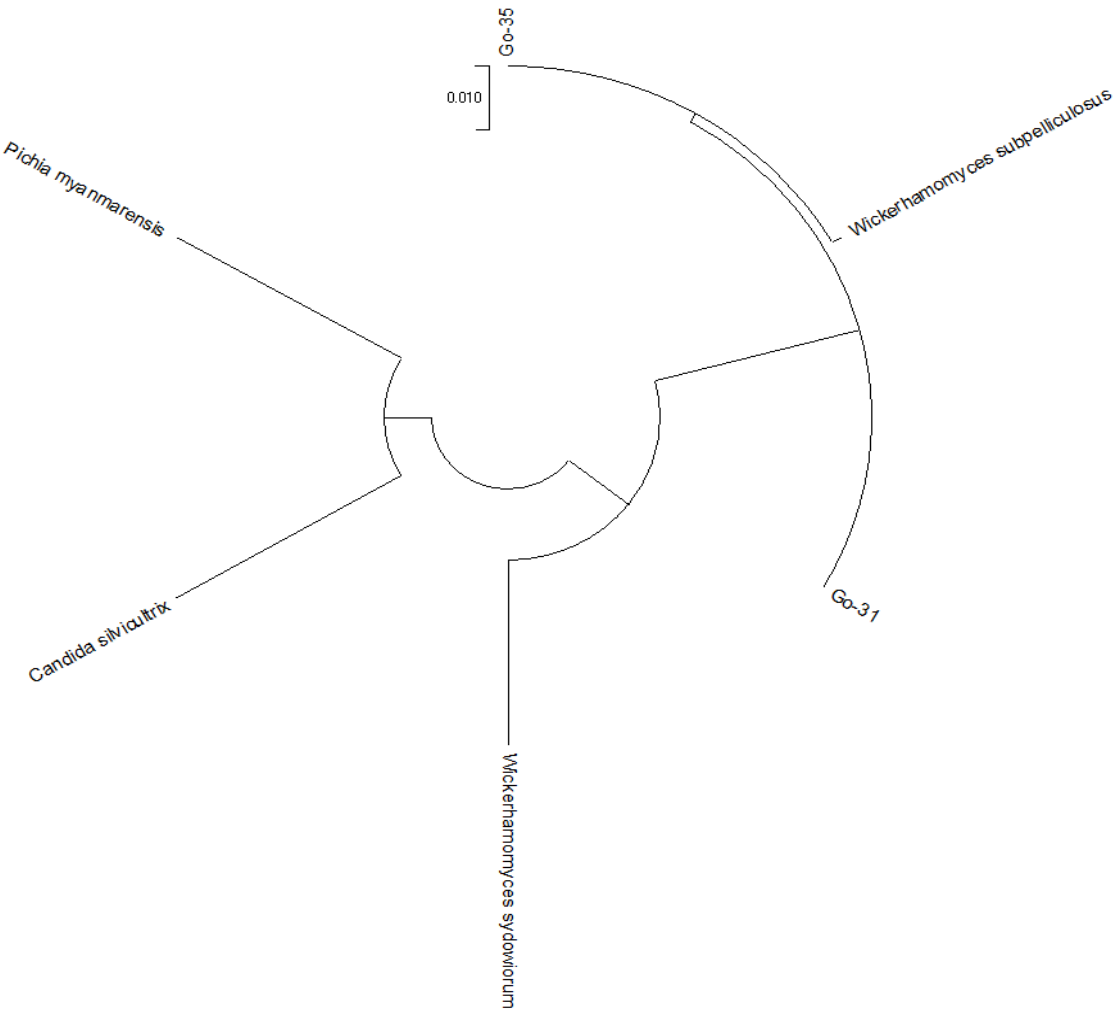

Figure S3. Phylogenetic tree analyses of the ITS sequences of the *Wikerhamomyces subpelliculosus* isolated from *gochujang* products constructed in MEGA 6 software by employing UPGMA method.

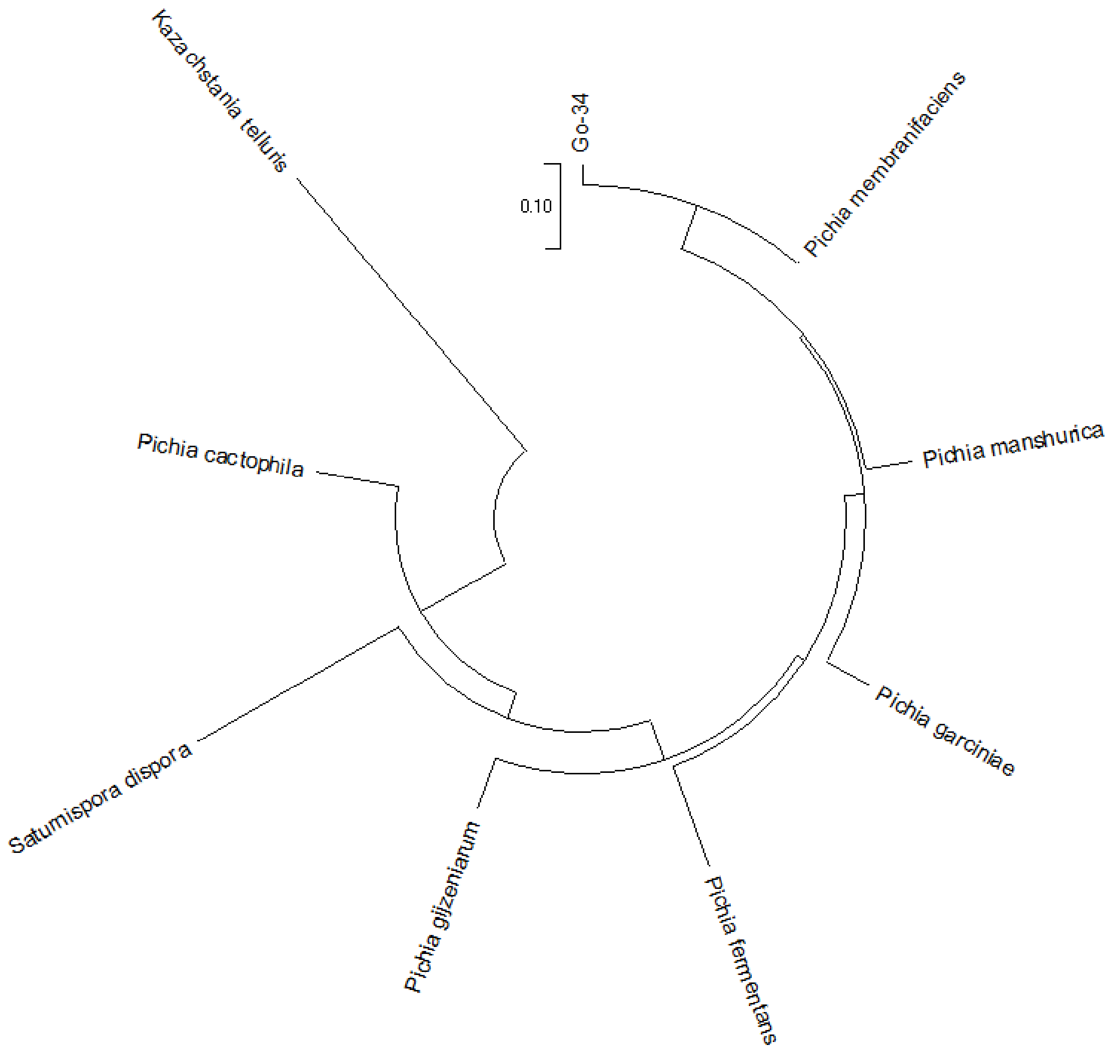

Figure S4. Phylogenetic tree analyses of the ITS sequences of the *Pichia membranifaciens* isolated from *gochujang* products constructed in MEGA 6 software by employing UPGMA method.

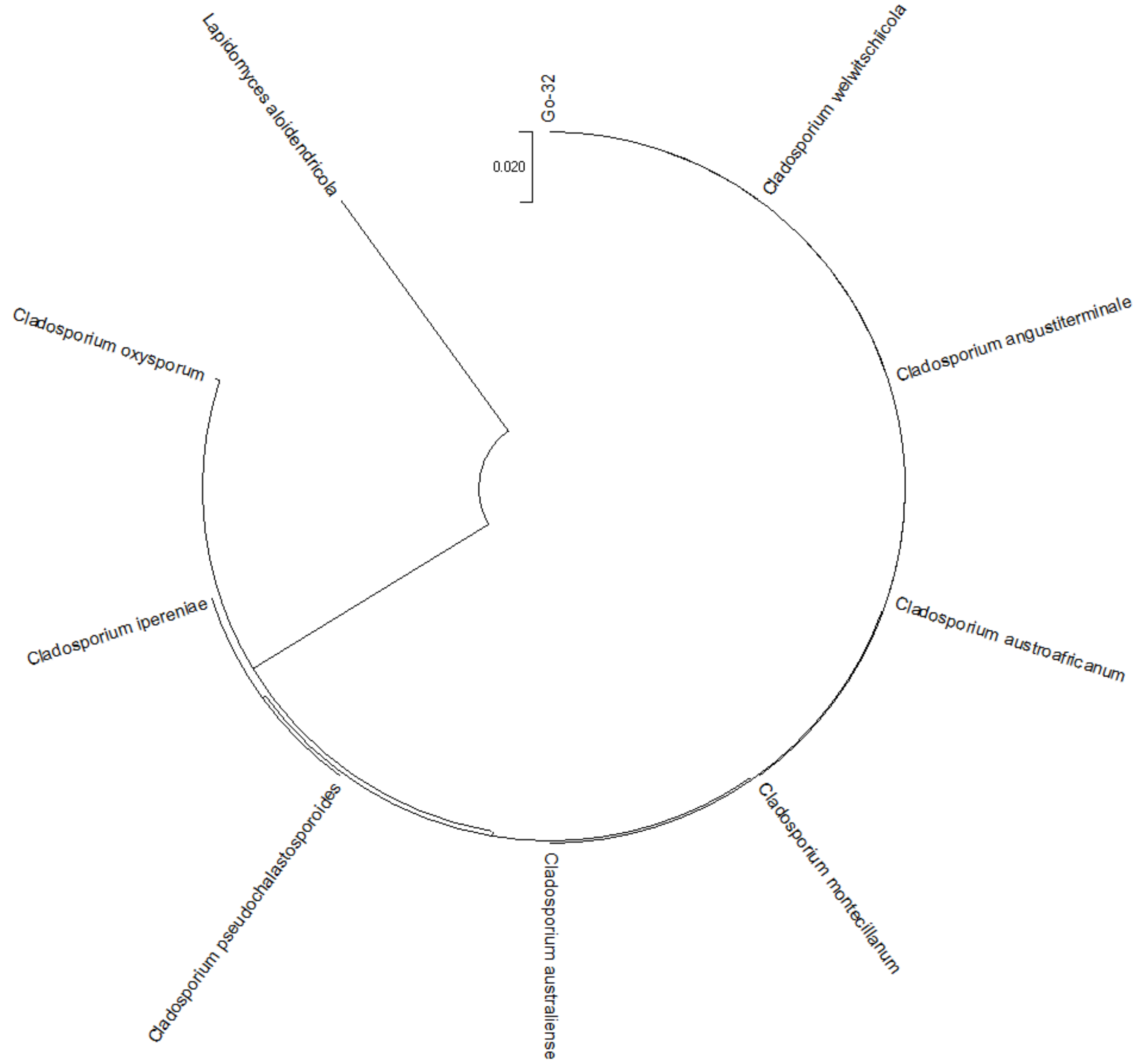

Figure S5. Phylogenetic tree analyses of the ITS sequences of the *Cladosporium welwitschiicola* isolated from *gochujang* products constructed in MEGA 6 software by employing UPGMA method.

Table S1. Various alcohol contents in *gochujang* products.

Product code	Methanol (%)	Ethanol (%)	1-Propanol (%)	1-Butanol (%)	1-Pentanol (%)	Total
Go-1	0.000	3.157	ND*	ND	ND	3.157
Go-2	0.113	0.391	ND	ND	ND	0.504
Go-3	0.120	0.139	ND	ND	ND	0.259
Go-4	0.078	0.426	ND	ND	ND	0.504
Go-5	0.064	1.723	0.005	ND	0.002	1.795
Go-6	0.093	1.084	0.004	0.003	0.003	1.186
Go-7	0.047	2.767	0.016	0.007	0.003	2.840
Go-8	0.027	1.117	0.004	0.003	ND	1.151
Go-9	0.038	1.159	0.005	0.001	ND	1.204
Go-10	0.031	2.342	0.008	0.001	0.002	2.384
Go-11	0.060	2.392	0.006	ND	0.002	2.461
Go-12	0.026	4.010	ND	0.006	0.004	4.047
Go-13	0.022	1.183	ND	ND	0.002	1.206
Go-14	0.012	0.869	0.003	0.002	0.002	0.888
Go-15	0.023	0.874	0.005	0.002	0.001	0.906
Go-16	ND	0.000	ND	ND	ND	0.000
Go-17	0.022	1.375	0.007	0.004	0.002	1.411
Go-18	0.087	1.802	ND	ND	ND	1.889
Go-19	0.000	0.120	ND	ND	ND	0.120
Go-20	0.059	0.438	ND	ND	0.001	0.498
Go-21	0.008	0.318	ND	ND	0.002	0.327
Go-22	0.021	0.598	ND	ND	0.000	0.618
Go-23	0.076	3.219	0.014	0.010	0.005	3.324
Go-24	0.032	1.218	ND	ND	ND	1.250

Go-25	0.135	0.841	ND	ND	0.001	0.977
Go-26	0.078	4.278	ND	ND	ND	4.356
Go-27	0.048	2.365	0.006	0.005	ND	2.424
Go-28	0.019	0.880	0.004	0.002	0.002	0.908
Go-29	0.020	0.624	0.009	0.002	ND	0.656
Go-30	0.028	0.392	0.007	ND	ND	0.426
Go-31	0.050	3.090	0.015	ND	ND	3.155
Go-32	0.037	0.351	ND	ND	ND	0.388
Go-33	0.036	4.944	0.008	0.002	0.002	4.991
Go-34	0.023	1.446	0.004	0.001	ND	1.474
Go-35	0.047	1.547	0.005	ND	0.001	1.600
Mean ± SD	0.045 ± 0.05	1.528 ± 1.23	0.007 ± 0.002	0.003 ± 0.002	0.002 ± 0.001	1.580 ± 1.28

ND-not detected.

Table S2. Various volatile compounds found in *gochujang* products.

Peak Number	Retention Time (min)	Area (%)	Compound Name
			**Go-1**
1	7.677	0.49	ethyl acetate
2	9.138	43.94	ethanol
3	15.971	0.28	1-propanol, 2-methyl-
4	18.982	0.14	1-propene, 3,3'-thiobis-
5	19.922	0.30	cyclopentasiloxane, decamethyl-
6	22.896	1.39	1-butanol, 3-methyl-
7	24.496	0.24	hexanoic acid, ethyl ester
8	25.567	0.13	1,3,6-octatriene, 3,7-dimethyl-, (e)-
9	25.748	0.35	styrene
10	27.342	0.15	1,3-dithiane
11	27.556	0.13	2-butanone, 3-hydroxy-
12	29.390	0.05	2(1h)-pyrimidinone, 4-(methylamino)-
13	29.790	0.09	1-butanol
14	30.156	0.09	dimethyl pyrazine
15	31.128	1.54	cyclohexasiloxane, dodecamethyl-
16	32.293	0.13	1-hexanol
17	32.481	0.13	tridecane, 2-methyl-, nonanal
18	34.082	0.19	4-methylpentyl 2-methylbutanoate
19	34.730	0.05	nonanal
20	35.276	0.11	butanoic acid, 3-methyl-, hexyl ester

21	35.505	0.13	pyrazine, trimethyl-
22	37.460	0.27	Octanoic acid, ethyl ester
23	38.446	0.07	1-Octen-3-ol
24	38.829	0.33	1H-Imidazole, 1,4-dimethyl-
25	39.540	0.06	1-Tetradecanamine
26	39.681	0.06	(4Z)-2-Methyl-4-Decene
27	40.020	0.48	2,3,5,6 Tetramethyl Pyrazine
28	40.110	0.73	Diallyl disulphide
29	40.448	0.28	(Z)-1-Allyl-2-(prop-1-en-1-yl)disulfane
30	40.895	0.30	Ethyl tridecanoate
31	41.356	0.11	Hydrazinecarbothioamide, N-ethyl-
32	41.474	0.23	(3E,5E)-Hepta-3,5-dien-2-one
33	41.803	1.03	Tetradecamethyl-cycloheptasiloxane
34	42.551	0.20	Benzaldehyde
35	42.709	0.17	Hexanoic acid, hexyl ester
36	43.165	0.08	Thiodiglycol
37	43.759	0.10	Nonanoic acid, ethyl ester
38	44.385	0.09	Cyclohexene, l-methyl-4-(1-methylethylidene)-
39	46.064	0.15	2-Butanone, 3-hydroxy-
40	46.768	0.24	Trisulfide, methyl 2-propenyl
41	46.908	0.07	Pyridine, 4-methoxy-
42	47.034	0.28	Hexadecanoic acid, ethyl ester
43	47.559	0.07	Dodecane, 2,6,10-trimethyl-
44	48.075	1.10	2-Acetyl-4,4-dimethyl-cyclopent-2-enone

45	48.659	0.10	1-Cyclohexene-l-carboxaldehyde, 2,6,6-trimethyl-
46	49.658	0.15	Benzeneacetaldehyde
47	49.815	0.17	Decanoic acid, ethyl ester
48	50.038	0.16	1,3-Cyclohexadiene-l-carboxaldehyde, 2,6,6-trimethyl-
49	50.307	0.23	Silanediol, dimethyl-
50	50.810	0.22	2-Furanmethanol
51	51.104	0.09	Chloroacetic acid, nonyl ester
52	51.198	0.16	Benzoic acid, ethyl ester
53	51.608	0.26	Cyclooctasiloxane, hexadecamethyl-
54	51.816	0.14	Butanedioic acid, diethyl ester
55	52.948	0.15	Valerena-4,7(11)-diene
56	57.184	1.25	Methyl salicylate
57	57.751	0.52	1-methoxycarbonyl-2-trideuteromethylaminoethene
58	58.014	0.52	Trisulfide, di-2-propenyl
59	59.710	0.08	Silanediol, dimethyl-
60	61.141	0.31	Dodecanoic acid, ethyl ester
61	61.625	0.21	Phenol, 2-methoxy-
62	62.605	0.16	Benzenemethanol
63	64.445	1.85	Benzeneethanol
64	64.806	0.18	1H-Benzimidazole, 2-methyl-
65	65.965	0.16	3-Buten-2-one, 4-(2,6,6-trimethyl- 1-cyclohexen-l-yl)-
66	70.972	0.11	l,6,10-Dodecatrien-3-ol, 3,7,11-trimethyl-, (E)-
67	71.490	1.03	Tetradecanoic acid, ethyl ester
68	74.051	0.41	Ethyl 13-methyl-tetradecanoate

69	76.352	0.16	D-Gluconic acid, 2,3,4,6-tetra-O-methyl-, .delta.-lactone
70	76.701	0.25	Phenol, 2-methoxy-4-(2-propenyl)-
71	77.950	0.35	2-Methoxy-4-vinylphenol
72	80.995	13.58	Hexadecanoic acid, ethyl ester
73	82.128	0.62	Ethyl-9-hexadecenoate
74	84.595	0.29	Dihydroactinidiolide
75	89.731	0.30	Octadecanoic acid, 17-methyl-, methyl ester
76	90.524	2.37	(E)-9-Octadecenoic acid ethyl ester
77	90.862	0.40	1,4,7,10,13,16-Hexaoxacyclooctacane
78	92.436	13.25	Linoleic acid ethyl ester
79	94.476	2.92	Ethyl 9,12,15-octadecatrienoate
			**Go-2**
1	6.463	0.16	Acetic acid, methyl ester
2	7.679	0.78	Acetic acid ethyl ester
3	7.956	0.72	Formic acid, propyl ester
4	8.368	0.08	Allyl ethyl ether
5	8.490	0.17	Butanal, 3-methyl-
6	9.112	11.46	Ethanol
7	15.270	0.09	Hexanal
8	15.996	0.09	1,2,15-Pentadecanetriol
9	19.920	0.31	Cyclopentasiloxane, decamethyl-
10	21.486	0.06	Hexanoic acid methyl ester
11	22.815	0.10	1-Butanol, 2-methyl-
12	22.896	0.26	1-Butanol 3-methyl-

13	24.257	0.12	Furan, 2-pentyl-
14	24.495	0.27	Ethyl hexanoate
15	27.562	0.10	2-Butanone, 3-hydroxy-
16	27.960	0.06	Octanal
17	30.157	0.20	Pyrazine, 2,6-dimethyl-
18	31.127	1.46	Cyclohexasiloxane, dodecamethyl-
19	32.294	0.11	4-Pentenal
20	32.476	0.13	Eicosane, 10-methyl-
21	34.548	0.12	Octanoic acid, methyl ester
22	34.728	0.07	Nonanal
23	35.273	0.11	Heneicosane
24	35.505	0.34	Pyrazine, trimethyl-
25	36.941	0.11	7-Oxa-bicyclo [4.1.0]heptane
26	37.460	0.58	Octanoic acid, ethyl ester
27	37.981	0.06	2-Furanmethanol, 5-ethenyltetrahydro-.alpha.,.alpha.,5-trimethyl-, trans-
28	38.343	0.05	Propanal, 3-(methylthio)-
29	38.440	0.17	1-Octen-3-ol
30	38.837	4.60	2-Furan-carboxaldehyde
31	39.150	0.03	1H-Imidazole 1,5-dimethyl-
32	39.693	0.09	1-Undecene, 7-methyl-
33	40.018	2.74	Pyrazine, tetramethyl-
34	40.485	0.16	Acetic acid
35	40.897	0.07	l-(Ethylsulfanyl)Ethylene
36	41.469	0.48	Ethanone, 1- (2-furanyl)-

37	41.796	2.00	Cycloheptasiloxane, tetradecamethyl-
38	42.551	1.45	Benzaldehyde
39	43.537	0.07	Decan-1-ol
40	43.759	0.08	Nonanoic acid, ethyl ester
41	43.891	0.11	2,3-Butanediol
42	44.387	1.18	L-Linalool
43	45.580	0.56	2-Furancarboxaldehyde, 5-methyl-
44	46.099	0.14	2,3-Butanediol
45	46.157	0.19	3-Methyl-3-butenyl ether
46	46.906	0.24	6-Methyl-3,5-heptadiene-2-one
47	47.562	0.14	Cyclohexanol, 2,6-dimethyl-
48	48.069	0.46	5-Hepten-1-ol, 2,6-dimethyl-
49	48.284	0.09	3-Cyclohexene-1-acetaldehyde, .alpha.,4-dimethyl-
50	48.560	0.52	Benzoic acid, methyl ester
51	49.655	1.22	Benzeneacetaldehyde
52	50.044	0.79	1,3-Cyclohexadiene-1-carboxaldehyde, 2,6,6-trimethyl-
53	50.294	0.48	Silanediol, dimethyl-
54	50.813	1.46	2- Furanmethanol
55	51.207	0.35	2-Furancarboxamide, N-methyl-
56	51.600	0.84	Cyclooctasiloxane, hexadecamethyl-
57	51.845	0.12	Trimethylsilyl Ester of Furan-3-Carboxylic Acid
58	52.634	0.07	2,6,6-Trimethyl-2-cyclohexene-1,4-dione
59	52.950	0.05	[4aR,8aR] - 1,2,3,4,4a,5,8,8a - octahydro - 1,1,4a,6 - tetramethyl -5- methylene-naphthalene
60	53.140	0.38	3-Cyclohexene-1-methanol, .alpha., alpha., 4-trimethyl-

61	56.367	0.15	Benzeneacetic acid, methyl ester
62	57.186	1.18	Methyl salicylate
63	57.823	0.14	Benzeneacetic acid, ethyl ester
64	58.920	0.25	Dodecanoic acid, methyl ester
65	59.435	0.18	Acetamide, N-(2-phenylethyl)-
66	59.728	0.08	Silanediol, dimethyl-
67	60.357	0.10	1,1,1,5,7,7,7-Heptamethyl-3,3-bis(trimethylsiloxy)tetrasiloxane
68	61.140	0.84	Dodecanoic acid, ethyl ester
69	61.629	0.28	Phenol, 2-methoxy-
70	62.602	0.21	Benzyl alcohol
70	64.440	2.26	Benzeneethanol
71	65.325	0.12	Benzeneacetaldehyde, alpha.- ethylidene-
72	65.968	0.20	Trans-.beta.-Ionone
73	67.369	0.41	Ethanone, 1-(1H-pyrrol-2-yl)
74	69.007	0.20	Phenol
75	69.553	0.56	Myristic acid, methyl ester
76	70.249	0.37	Guaiacol, 4-ethyl-
77	71.490	1.55	Tetradecanoic acid, ethyl ester
78	74.056	0.20	Ethyl 13-methyl-tetradecanoate
79	77.075	0.16	Phenol, 2-ethyl-
80	77.953	0.78	2-Methoxy-4-vinylphenol
81	79.292	3.36	Hexadecanoic acid, methyl ester
82	80.476	0.24	9-Hexadecanoic acid, methyl ester, (Z)-
83	80.989	7.26	Hexadecanoic acid, ethyl ester

84	82.125	0.64	Ethyl 9-hexadecenoate
85	84.595	0.42	2(4H)-Benzofuranone, 5,6,7,7a-tetrahydro-4,4,7a-trimethyl-, (R)-
86	86.493	0.25	4-Vinylphenol
87	89.092	1.97	9-Octadecenoic acid (Z)-, methyl ester
88	89.737	0.21	Octadecanoic acid, ethyl ester
89	90.531	5.01	Ethyl (9Z)-9-Octadecenoate
90	90.871	2.29	1,4,7,10,13,16-Hexaoxacyclooctadecane
91	91.056	7.79	9,12-Octadecadienoic acid, methylester, (E,E)-
92	92.441	17.58	Linoleic acid ethyl ester
93	93.505	0.84	9,12,15-Octadecatrienoic acid, methyl ester, (Z,Z,Z)-
94	94.478	2.45	Ethyl 9,12,15-octadecatrienoate
			**Go-3**
1	6.462	0.17	Acetic acid, methyl ester
2	7.679	0.33	Acetic acid ethyl ester
3	7.957	1.53	Formic acid, propyl ester
4	8.489	0.22	Butanal, 3-methyl-
5	9.108	13.41	Ethanol
6	15.241	0.23	4-Isopropylamino-2-methylbutan-2-o
7	16.109	0.09	2,2’-Oxybis (acethydrazide)
8	19.742	0.11	Methyl-2,3,4-tri-o-methyl-.beta.-darabopyranoside- 5,5-d2
9	19.915	1.02	Cyclopentasiloxane, decamethyl-
10	22.834	0.15	1-Butanol, 2-methyl-
11	22.913	0.36	1-Butanol, 3-methyl-
12	22.248	0.18	Furan, 2-pentyl-

13	24.499	0.09	1-Propene, 3-(methylthio)-
14	27.328	0.18	(Z)-1-Methyl-2-(prop-1-en-1-yl)disulfane
15	27.571	0.24	Acetoin
16	27.958	0.18	Octanal
17	28.667	0.06	Cyclohexane, l,4-dimethyl-
18	30.155	2.39	Pyrazine, *2*,5-dimethyl-
19	30.560	0.20	Pyrazine., 2,6-dimethyl-
20	31.125	3.28	Cyclohexasiloxane, dodecamethyl-
21	32.297	0.17	1-Hexanol
22	32.475	0.57	Tridecane, 2-methyl-
23	33.709	0.41	Dimethyl trisulfide
24	34.727	0.23	Nonanal
25	35.271	0.19	Tetradecane
26	35.506	1.87	Pyrazine, trimethyl-
27	36.938	0.21	Octenal
28	37.979	0.11	2-Furanmethanol, 5-ethenyltetrahydro-.alpha., .alpha.,5-trimethyl-, cis-
29	38.106	0.21	Pyrazine, 2-ethyl-3,5-dimethyl-
30	38.442	0.32	1-Octen-3-ol
31	38.830	3.40	2-furan-carboxaldehyde
32	39.164	0.18	Pyrazine, 2-ethyl-3,5-dimethyl-
33	39.688	2.02	Acetic acid
34	40.017	3.75	Pyrazine, tetramethyl-
35	40.853	0.11	l-Carbomethoxy-4-formylcyclohexane
36	41.354	0.10	l-Methoxy-3-(2-hydroxyethyl)nonane

37	41.468	0.70	(3E,5E)-Hepta-3,5-dien-2-one
38	41.796	3.18	Cycloheptasiloxane, tetradecamethyl-
39	42.442	0.16	2,3,5-Trimethyl-6-ethylpyrazine 4-hydroxy-1,3-benzenedicarboxaldehyde
40	42.551	2.00	Benzaldehyde
41	42.712	0.15	1,3-Dithiolane
42	43.555	0.31	Cis-4-Nonene
43	43.891	0.45	2,3-Butanediol
44	44.385	0.48	Cyclohexene, 1-methyl-4-(1-methylethylidene)-
45	44.935	0.24	Eicosane, 7-hexyl-
46	45.578	0.33	2-Furancarboxaldehyde, 5-methyl-
47	46.148	0.78	2-Octanol, (R)-
48	46.761	0.09	Butanoic acid, 4,4'-dithiobis-
49	46.907	0.33	Ethanone, l-(2-methyl-l-cyclopenten-l-yl)-
50	47.244	1.34	Propanoic acid, 2-methyl-
51	47.563	0.59	Hexadecane
52	47.697	0.14	Hydrazine, 1,1-diethyl-
53	49.661	1.47	Benzeneacetaldehyde
54	50.031	0.81	Prop-2-enyl (2S)-2-methyl-3-phenylpropionate
55	50.305	0.86	Silanediol, dimethyl-
56	50.812	1.83	2-Furanmethanol
57	51.202	0.7	2-ethyl-5-propylthiophene
58	51.603	1.14	Cyclooctasiloxane, hexadecamethyl-
59	52.316	6.93	Pentanoic acid
60	52.529	6.99	Butanoic acid, 3-methyl-

61	53.137	0.50	DL-Serine
62	56.372	0.46	Benzeneacetic acid, methyl ester
63	57.184	1.93	Methyl salicylate
64	57.826	0.22	Ethyl 2-phenylacetate
65	59.702	0.15	Silanediol, dimethyl-
66	61.627	0.37	Phenol, 2-methoxy-
67	62.600	1.49	Benzenemethanol
68	64.443	1.15	Benzeneethanol
69	65.965	0.23	(3E)-4-(2,6,6-Trimethyl-1-Cyclohexen-1-yl)-3-Buten-2-one
70	67.360	0.41	Ethanone, 1-(1H-pyrrol-2-yl)-
71	67.937	0.18	4H-Pyran-4-one, 3-hydroxy-2-methyl
72	69.553	0.30	Myristic acid, methyl ester
73	70.966	0.26	(6E)-3-Hydroxy-3,7,11-trimethyl-6,10-dodecadienyl acetate
74	71.488	0.49	Tetradecanoic acid, ethyl ester
75	77.949	6.36	2-Methoxy-4-vinylphenol
76	79.288	1.45	Hexadecanoic acid, methyl ester
77	80.984	2.00	Hexadecanoic acid, ethyl ester
78	84.591	0.35	2(4H)-Benzofuranone, 5,6,7,7a-tetrahydro-4,4,7a-trimethyl -, (R)-
79	86.497	1.53	4-Vinylphenol
80	91.049	2.09	9,12-Octadecadienoic acid (Z,Z)-,methyl ester
81	92.426	3.13	Linoleic acid ethyl ester
82	94.209	4.8	1,4,7,10,13,16-Hexaoxacyclooctadecane
			**Go-4**
1	7.673	0.24	Acetic acid ethyl ester

2	7.949	0.31	Acetic acid hydroxy-
3	8.482	0.13	Butanal, 3-methyl-
4	9.103	10.73	Ethanol
5	15.910	0.13	1-Propanol, 2-methyl-
6	19.927	0.57	Cyclopentasiloxane, decamethyl-
7	22.804	0.16	1-Butanol, 2-methyl-
8	22.884	0.72	1-Butanol, 3-methyl-
9	24.267	0.06	Furan, 2-pentyl-
10	24.496	0.12	Hexanoic acid, ethyl ester
11	27.553	0.08	2-Butanone, 3-hydroxy-
12	27.964	0.09	Octanal
13	30.148	0.78	Pyrazine, 2,5-dimethyl-
14	31.125	1.67	Cyclohexasiloxane, dodecamethyl-
15	31.399	0.06	2-Butanol, 3-methyl-
16	32.286	0.06	1-Hexanol
17	32.478	0.15	Heneicosane
18	35.275	0.08	Undecane, 2,7-dimethyl-
19	35.500	0.65	Pyrazine, trimethyl-
20	36.939	0.06	2-Hydroxy-1-oxacyclooctane
21	37.460	0.11	Octanoic acid, ethyl ester
22	37.980	0.11	Ethyl 2-(5-methyl-5-vinyltetrahydrofuran-2-yl)propan-2-yl carbonate
23	38.105	0.06	Pyrazine, 3-ethyl-2,5-dimethyl-
24	38.443	0.09	1-Octen-3-ol
25	38.834	4.79	2-furan-carboxaldehyde

26	39.150	0.14	Pyrazine, 2-ethyl-3,5-dimethyl-
27	39.691	0.13	3-Undecene, 10-methyl-
28	40.015	2.49	2,3,5,6 Tetramethyl Pyrazine
29	41.467	0.69	Ethanone, 1-(2-furanyl)-
30	41.800	1.26	Tetradecamethylcycloheptasiloxane
31	42.438	0.22	2,3,5-Trimethyl-6-ethylpyrazine
32	42.548	0.4	Benzaldehyde
33	43.753	0.04	Nonanoic acid, ethyl ester
34	44.384	0.50	L-Linalool
35	44.933	0.07	Octadecane
36	45.577	0.63	2-Furancarboxaldehyde, 5-methyl-
37	45.808	0.05	1H-Pyrazole-4-carboxylic acid
38	46.156	0.24	Oxirane, 2-methyl-3-propyl-, cis-
39	46.902	0.28	6-Methyl-3,5-heptadiene-2-one
40	47.040	0.09	.Alpha.-D-Glucopyranoside, methyl 2,3,4,6-tetra-O-methyl-
41	47.554	0.14	Hexadecane
42	48.072	0.41	2-Allyl-2-methyl-l,3-cyclopentanedione
43	48.276	0.1	Decane-3,4-d2
44	48.472	0.07	3-Cyclohexene-l-acetaldehyde, .alpha.,4-dimethyl-
45	48.558	0.06	Benzoic acid, hydrazide
46	48.646	0.16	1H-Inden-1-ol, 3a,4,5,6,7,7a-hexahydro-l-methyl-
47	49.645	0.83	Benzeneacetaldehyde
48	50.041	0.52	1,3-Cyclohexadiene-1-carboxaldehyde, 2,6,6-trimethyl-
49	50.812	2.33	2-Furanmethanol

50	51.204	0.11	3H-Pyrrol-3-one, 1-ethyl-1,2-dihydro-2-methyl-
51	51.606	0.41	Cyclooctasiloxane, hexadecamethyl-
52	51.814	0.10	Butanedioic acid, diethyl ester
53	52.619	0.07	2-Cyclohexene-l,4-dione, 2,6,6-trimethyl-
54	52.951	0.05	Cis-(-)-2,4a,5,6,9a-Hexahydro-3,5,5,9-tetramethyl(lH)benzocycloheptene
55	53.150	0.19	3-Cyclohexene-l-methanol, .alpha., .alpha.,4-trimethyl-
56	56.368	0.16	Benzeneacetic acid, methyl ester
57	57.185	0.23	Benzoic acid, 2-hydroxy-, methyl ester
58	57.829	0.21	Ethyl 2-phenylacetate
59	58.921	0.12	Dodecanoic acid, methyl ester
60	59.428	0.10	Acetic acid, 2-phenylethyl ester
61	59.751	0.04	Silanediol, dimethyl-
62	60.355	0.04	Cyclononasiloxane, octadecamethyl-
63	61.141	0.52	Dodecanoic acid, ethyl ester
64	61.631	0.46	Phenol, 2-methoxy-
65	62.604	0.51	Benzyl alcohol
66	64.442	1.64	Benzeneethanol
67	65.322	0.19	Benzeneacetaldehyde, .alpha.-ethylidene-
68	65.971	0.17	3-Buten-2-one, 4-(2,6,6-trimethyl-1-cyclohexen-l-yl)-
69	67.366	0.77	Ethanone, l-(lH-pyrrol-2-yl)-
70	69.000	0.38	Phenol
71	69.556	0.38	Myristic acid, methyl ester
72	69.937	0.16	1H-Pyrrole-2-carboxaldehyde
73	70.243	0.24	3,5-Diacetyl-1H-pyrazole

74	70.968	0.09	l,6,10-Dodecatrien-3-ol, 3,7,11-trimethyl-
75	71.489	1.35	Tetradecanoic acid, ethyl ester
76	72.189	0.16	Pentadecanoic acid, methyl ester
77	72.454	0.07	(2E)-5-Methyl-2-phenyl-2-hexenal
78	72.905	0.10	Tetradecanoic acid, 12-methyl-, methyl ester
79	73.662	0.12	1H-Pyrrole-2-carboxaldehyde, 1-methyl-
80	74.047	0.38	Pentadecanoic acid, ethyl ester
81	74.767	0.23	Ethyl 13-methyl-tetradecanoate
82	76.350	0.11	Pentadecanoic acid, ethyl ester
83	77.950	1.96	2-Methoxy-4-vinylphenol
84	79.294	4.28	Hexadecanoic acid, methyl ester
85	80.476	0.14	9-Hexadecenoic acid, methyl ester, (Z)-
86	80.991	11.47	Hexadecanoic acid, ethyl ester
87	81.280	0.29	4H-Pyran-4-one, 2,3-dihydro-3,5-di hydroxy-6-methyl-
88	82.129	0.53	Ethyl 9-hexadecenoate
89	84.599	0.42	Dihydroactinidiolide
90	86.495	0.26	4-Vinylphenol
91	88.250	0.16	Heptadecanoic acid, 16-methyl-, methyl ester
92	89.094	1.93	9-Octadecenoic acid, methyl ester, (E)-
93	89.738	0.36	Octadecanoic acid, ethyl ester
94	90.534	4.85	Ethyl (9Z)-9-Octadecenoate
95	90.916	1.98	5-Hydroxymethylfurfural
96	91.054	5.95	9,12-Octadecadienoic acid (Z,Z)-,methyl ester
97	92.438	13.78	Linoleic acid ethyl ester

98	93.506	1.04	9,12,15-Octadecatrienoic acid, methyl ester, (Z,Z,Z)-
99	94.202	4.05	1,4,7,10,13,16-Hexaoxacyclooctadecane
100	94.473	2.52	Ethyl 9,12,15-octadecatrienoate
101	95.308	0.29	21-Krone-7
			**Go-5**
1	7.675	0.59	Acetic acid ethyl ester
2	7.956	0.10	Formic acid. ethyl ester
3	9.113	17.60	Ethanol
4	15.321	0.05	DL-Cystathionine
5	15.907	0.31	1-Propanol, 2-methyl-
6	16.560	0.04	Ethanol, 2-bromo-
7	19.004	0.26	Diallyl sulfide
8	19.929	0.36	Cyclopentasiloxane, decamethyl-
9	22.804	0.23	1-Butanol, 2-methyl-
10	22.882	0.43	1-Butanol, 3-methyl-
11	24.498	0.09	Ethyl hexanoate
12	27.337	1.18	Disulfide, methyl 2-propenyl
13	27.544	0.26	2-Butanone, 3-hydroxy-
14	27.859	0.47	(Z)-l-Methyl-2-(prop-l-en-l-yl)disulfane
15	29.081	0.05	Pentanoic acid., 2-(methoxymethyl)-4-oxo-
16	30.145	0.16	Dimethyl pyrazine
17	31.126	1.86	Cyclohexasiloxane, dodecamethyl-
18	32.286	0.13	1-Hexanol
19	33.712	1.67	Dimethyl trisulfide

20	35.271	0.06	Methoxyacetic acid, 2-tridecyl ester
21	35.499	0.36	Pyrazine, trimethyl-
22	36.943	0.09	(E)-l-(Prop-l-en-l-yl)-2-propyldisulfane
23	37.462	0.13	Octanoic acid, ethyl ester
24	38.442	0.07	1-Octen-3-ol
25	38.833	0.48	2-furan-carboxaldehyde
26	40.023	8.75	2,3,5,6 Tetramethyl pyrazine
27	40.116	6.18	Diallyl disulfide
28	40.485	2.51	(E)-l-Allyl-2-(prop-l-en-l-yl)disulfane
29	41.466	0.14	Bicyclo[4.1.0]heptane, 1-methyl-
30	41.796	1.88	Tetradecamethylcycloheptasiloxane
31	42.436	0.36	4-Ethyl-2,5,6-Trimethylpyrimidine
32	42.550	0.46	Benzaldehyde
33	42.688	6.51	3H-l,2-Dithiole
34	43.760	0.06	Nonanoic acid, ethyl ester
35	44.377	0.09	L-Linalool
36	45.584	0.10	5 Methyl furfural
37	46.146	0.15	2-Butanol, 3-methyl-
38	46.769	6.98	Trisulfide, methyl 2-propenyl
39	47.033	0.07	1-Propene, l-(methylthio)-, (E)-
40	48.070	0.09	Neopentylidenecyclohexane
41	49.649	0.19	Benzeneacetaldehyde
42	49.812	0.10	Propanoic acid, 2-(phenylmethoxy)-, methyl ester
43	50.043	0.19	1,3-Cyclohexadiene-1-carboxaldehyde, 2,6,6-trimethyl-

44	50.808	0.18	2- Furanmethanol
45	51.202	0.06	5-Hepten-2-ol, 6-methyl-
46	51.603	0.65	Cyclooctasiloxane, hexadecamethyl-
47	52.084	0.05	2, 6 -Octadienal, 3,7-dimethyl-, (Z)
48	53.139	0.06	3-Cyclohexene-l-methanol, .alpha., .alpha.,4-trimethyl-
49	55.069	4.22	3-Vinyl-l,2-dithiacyclohex-4-ene
50	57.182	0.68	Methyl salicylate
51	57.824	0.15	Acetic acid, phenyl-, ethyl ester
52	58.023	11.93	Trisulfide, di-2-propenyl
53	59.171	0.09	2,4-Decadienal, (E,E)-
54	59.693	0.10	Silanediol, dimethyl-
55	60.358	0.10	Cyclononasiloxane, octadecamethyl-
56	60.846	1.72	3-Vinyl-l,2-dithiacyclohex-5-ene
57	61.138	0.23	Dodecanoic acid, ethyl ester
58	61.622	0.21	Phenol, 2-methoxy-
59	64.436	1.20	Benzeneethanol
60	65.965	0.12	3-Buten-2-one, 4-(2,6,6-trimethyl-1-cyclohexen-l-yl)-
61	67.363	0.16	Ethanone, l-(lH-pyrrol-2-yl)-
62	68.150	0.09	7-Amino-4-methyl-2-quinolinol
63	71.487	0.82	Tetradecanoic acid, ethyl ester
64	74.048	0.12	Pentadecanoic acid, ethyl ester
65	74.491	0.12	Acetyl butyl disulfide
66	76.354	0.08	15-Crown-5
70	76.699	0.05	1,4,7,10,13,16-Hexaoxacyclooctadecane

67	77.950	2.28	2-Methoxy-4-vinylphenol
68	79.295	0.15	Hexadecanoic acid, methyl ester
69	80.983	5.85	Hexadecanoic acid, ethyl ester
70	82.125	0.21	Ethyl 9-hexadecenoate
71	84.591	0.23	2(4H)-Benzofuranone, 5,6,7,7a-tetrahydro-4,4,7a-trimethyl-, (R)-
72	86.495	0.67	Benzofuran, 2,3-dihydro-
73	90.526	0.66	9-Octadecenoic acid (Z)-, ethyl ester
74	92.426	3.13	Linoleic acid ethyl ester
75	94.475	1.79	1,4,7,10,13,16-Hexaoxacyclooctadecane
			**Go-6**
1	7.677	0.83	Acetic acid ethyl ester
2	7.953	0.20	Formic acid, ethyl ester
3	9.110	11.96	Ethanol
4	15.905	0.19	1-Propanol, 2-methyl-
5	16.994	0.04	2-Butanol
6	18.999	0.22	1-Propene, 3,3'-thiobis-
7	19.929	0.27	Cyclopentasiloxane, decamethyl-
8	22.808	0.12	1-Butanol 2-methyl-
9	22.883	0.27	1-Butanol 3-methyl-
10	24.264	0.05	Furan, 2-pentyl-
11	24.499	0.19	Hexanoic acid, ethyl ester
12	26.267	0.63	(Z)-l-Methyl-2-(prop-l-en-l-yl)disulfane
13	27.338	0.57	Disulfide, methyl 2-propenyl
14	27.548	0.21	2-Butanone, 3-hydroxy-

15	30.146	0.22	Pyrazine, 2,5-dimethyl-
16	31.128	1.19	Cyclohexasiloxane, dodecamethyl-
17	32.287	0.24	1-Hexanol
18	33.714	0.25	Trisulfide, dimethyl
19	33.939	0.25	Dimethyl trisulfide
20	34.740	0.05	Ethane, 1,1-diethoxy-
21	35.269	0.06	Tetradecane
22	35.497	0.46	Pyrazine, trimethyl-
23	36.947	0.22	(Z)-l-(Prop-l-en-l-yl)-2-propyldisulfane
24	37.458	0.24	Octanoic acid, ethyl ester
25	38.440	0.14	1-Octen-3-ol
26	38.828	0.31	2-furan-carboxaldehyde
27	39.175	1.06	(Z)-l-Allyl-2-(prop-l-en-l-yl)disulfane
28	40.018	3.76	2,3,5,6 Tetramethyl pyrazine
29	40.115	4.96	Diallyl disulphide
30	40.485	2.06	(E)-l-Allyl-2-(prop-l-en-l-yl)disulfane
31	41.470	0.20	(3E,5E)-Hepta-3,5-dien-2-one
32	41.799	1.42	Tetradecamethylcycloheptasiloxane
33	42.436	0.25	2,3,5-Trimethyl-6-ethylpyrazine
34	42.549	0.18	Benzaldehyde
35	42.689	5.59	3H-l,2-Dithiole
36	43.546	0.06	2-Nonenal, (E)-
37	43.756	0.09	Nonanoic acid, ethyl ester
38	43.875	0.18	Ethanol, 2,2'-oxybis-

39	44.380	0.14	l,6-Octadien-3-ol, 3,7-dimethyl-
40	45.589	0.16	3,5-Octadien-2-one
41	45.803	0.11	(2S,6S)-Bicyclo[2.2.2]octane-2,6-diol
42	46.097	0.33	2,3-Butanediol
43	46.765	3.98	Trisulfide, methyl 2-propenyl
44	46.893	0.27	6-Methyl-3, 5-heptadiene-2-one
45	47.563	0.08	Dodecane, 2,6,10-trimethyl-
46	48.073	0.09	Neopentylidenecyclohexane
47	48.204	0.04	Cis-anti-cis Dicyclohexyl-18-crown-6
48	48.629	0.00	1-Cyclohexene-l-carboxaldehydej 2,6,6-trimethyl-
49	49.653	0.23	Phenyl acetaldehyde
50	49.809	0.18	Decanoic acid, ethyl ester
51	50.042	0.31	1,3-Cyclohexadiene-l-carboxaldehyde, 2,6,6-trimethyl-
52	50.335	0.17	(S-Nitroso)thiomethane
53	50.810	0.45	2- Furanmethanol
54	51.192	0.14	Benzoic acid, ethyl ester
55	51.604	0.40	Cyclooctasiloxane, hexadecamethyl-
56	51.805	0.07	Thiazole, 5-ethoxy-
57	53.142	0.07	3-Cyclohexene-l-methanol, .alpha.,.alpha.,4-trimethyl-
58	55.070	3.18	3-Vinyl-l,2-dithiacyclohex-4-ene
59	57.178	0.86	Benzoic acid, 2-hydroxy-, methyl ester
60	57.825	0.24	Acetic acid, phenyl-, ethyl ester
61	58.025	12.54	Trisulfide, di-2-propenyl
62	59.171	0.15	2,4-Decadienal, (E,E)-

63	59.732	0.06	Silanediol, dimethyl-
64	60.359	0.07	Cyclononasiloxane, octadecamethyl-
65	60.844	1.31	3-Vinyl-l,2-dithiacyclohex-5-ene
66	61.138	0.59	Dodecanoic acid, ethyl ester
67	61.620	0.29	Ethanone, l-(l-cyclohexen-l-yl)-
68	62.606	0.11	Benzyl alcohol
69	64.441	0.83	Benzeneethanol
70	65.316	0.40	Benzeneacetaldehyde, .alpha.-ethyl idene-
71	65.961	0.18	Trans-.beta.-Ionone
72	67.363	0.17	Ethanone, l-(lH-pyrrol-2-yl)-
73	71.488	1.56	Tetradecanoic acid, ethyl ester
74	74.050	0.27	Pentadecanoic acid, ethyl ester
75	74.492	0.30	1,2-Dithiolane
76	74.762	0.16	Ethyl 13-methyl-tetradecanoate
77	75.087	0.04	1,4,7,10,13,16, 19-Heptaoxa-2-cycloheneicosanone
78	76.349	0.16	l,2-Dimethyl-4-heptylcyclohexane
79	76.690	0.05	5-Methoxy-2-allylphenol
80	77.945	3.35	2-Methoxy-4-vinylphenol
81	78.782	0.05	12-Crown-4
82	79.288	0.53	Hexadecanoic acid, methyl ester
83	80.989	11.48	Hexadecanoic acid, ethyl ester
84	82.126	0.36	Ethyl 9-hexadecenoate
85	84.593	0.33	2(4H)-Benzofuranone, 5,6,7,7a- tetrahydro-4,4,7a-trimethyl-, (R)-
86	86.492	1.13	4-Vinylphenol

87	89.734	0.27	Octadecanoic acid. 17-methyl-, methyl ester
88	90.526	1.57	Ethyl oleate
89	91.047	1.5	1,4,7,10,13,16-Hexaoxacyclooctadecane
90	92.429	6.3	Linoleic acid ethyl ester
91	94.473	2.58	Ethyl 9,12,15-octadecatrienoate
92	95.142	0.85	21-Krone-7
			**Go-7**
1	7.676	0.72	Ethyl Acetate
2	7.832	0.07	Ethane, 1,1-diethoxy-
3	7.958	0.24	Hydroxyacetic acid, hydrazide
4	9.138	49.26	Ethanol
5	16.014	0.98	l-Propanol, 2-methyl-
6	17.596	0.10	3-Methylbutan-2-yl propyl carbonate
7	19.921	0.37	Cyclopentasiloxane, decamethyl-
8	22.903	4.51	1-Butanol, 3-methyl- (impure)
9	24.492	0.15	Ethyl hexanoate
10	27.560	0.21	2-Butanone, 3-hydroxy-
11	29.782	0.05	Formic acid, butyl ester
12	30.177	0.07	2-Heptanol
13	31.127	1.32	Cyclohexasiloxane, dodecamethyl-
14	31.402	0.06	2-Hexanol, (R)-
15	32.287	0.08	4-Pentenal
16	32.477	0.11	Tridecane, 2-methyl-
17	32.765	0.06	(1H-Imidazol-4-yl)oxoacetic acid

18	33.712	0.07	1-Propanol, 3-ethoxy-
19	35.275	0.05	10-Methylnonadecane
20	35.505	0.05	Pyrazine, trimethyl-
21	37.455	0.39	Octanoic acid, ethyl ester
22	37.976	0.07	2-(5-Methyl-5-vinyltetrahydro-2-furanyl)-2-propanol
23	38.438	0.13	l-Octen-3-ol
24	38.822	0.47	Furfural
25	39.686	0.10	4-Decene, 2-methyl-, (E)-
26	40.012	0.57	2,3,5,6 Tetramethyl pyrazine
27	41.467	0.29	1,4-Hexadiene, 2,5-dimethyl-
28	41.801	1.17	Cycloheptasiloxane, tetradecamethy1-
29	42.547	0.34	Benzaldehyde
30	43.755	0.08	Nonanoic acid, ethyl ester
31	44.381	0.59	Linalool
32	44.932	0.05	Pentadecane, 2-methyl-
33	45.586	0.11	2-Furancarboxaldehyde, 5-methyl-
34	45.808	0.06	3,5-Octadien-2-one
35	46.084	0.95	1,3-Butanediol
36	46.912	0.07	2-Cyclopenten-1-one, 3,5,5-trimethyl-
37	47.033	0.27	Pentadecanoic acid, ethyl ester
38	47.555	0.07	Cyclohexanol, 2,6-dimethyl-
39	48.071	0.51	Neopentylidenecyclohexane
40	48.659	0.02	3-Ethyl-2-ethoxy-phenol
41	49.644	0.20	Benzeneacetaldehyde

42	49.813	0.45	Decanoic acid, ethyl ester
43	50.038	0.21	1,3-Cyclohexadiene-l-carboxaldehyde, 2,6,6-trimethyl-
44	50.297	0.17	Silanediol, dimethyl-
45	50.808	0.73	2-Furanmethanol
46	51.201	0.13	2-Isopropylfuran
47	51.606	0.33	Cyclooctasiloxane, hexadecamethyl-
48	51.821	0.10	Butanedioic acid, diethyl ester
49	52.950	0.09	5-Hydroxy-6-methoxy-8-[(4-amino-l-methylbutyl)amino]quinoline trihydrobromide
50	53.136	0.30	2-(4-Methyl-3-cyclohexen-l-yl)-2-propanol
51	57.181	0.73	Methyl salicylate
52	58.933	0.22	2,6-Di-O-methyl-d-galactopyranose
53	59.433	1.81	Acetic acid, 2-phenylethyl ester
54	59.723	0.07	Silanediol, dimethyl-
55	59.858	0.07	2-Buten-l-one, l-(2,6,6-trimethyl- 1,3-cyclohexadien-l-yl)-, (E)-
56	60.359	0.03	Cyclononasiloxane, octadecamethyl-
57	61.137	0.83	Dodecanoic acid. ethyl ester
58	61.617	0.13	Phenol, 2-methoxy-
59	63.055	0.15	Acetic acid, methoxy-, 2-phenylethyl ester
60	64.441	10.15	Benzeneethanol
61	65.964	0.15	3-Buten-2-one, 4-(2,6,6-trimethyl-l-cyclohexen-l-yl) -
62	67.363	0.56	Ethanone, 1-(lH-pyrrol-2-yl)-
63	67.862	0.15	4,5-Diamino-6-hydroxypyrimidine
64	69.554	0.11	Myristic acid, methyl ester
65	70.226	0.12	2(3H)-Furanone, dihydro-5-pentyl-

66	71.028	0.12	DL-cis-2-amino-trans-2-hydroxy-trans-decalin-9-carboxylic lactam
67	71.486	1.22	Tetradecanoic acid, ethyl ester
68	72.734	0.05	18-Crown-6, [2-(tetramethyl-1,3,2-dioxaborolan-2-yl)phenyl]-
69	74.049	0.11	Ethyl 13-methyl-tetradecanoate
70	76.162	0.13	2-Methyl-trans-2,trans-4-hexadiene dioic acid
71	76.347	0.08	12-Crown-4
72	77.951	0.26	4-Vinyl-2-methoxy-phenol
73	79.290	0.70	Hexadecanoic acid, methyl ester
74	80.983	6.49	Hexadecanoic acid, ethyl ester
75	82.127	0.27	Ethyl-9-hexadecenoate
76	84.590	0.20	Dihydroactinidiolide
77	86.494	0.17	2-Methyl-l,3-dithiacyclopentane
78	89.091	0.08	Octaethylene glycol monododecyl ether
79	90.523	0.86	(E)-9-Octadecenoic acid ethyl ester
80	92.427	3.17	Linoleic acid ethyl ester
81	94.473	1.77	Ethyl 9,12,15-octadecatrienoate
82	96.983	0.92	1,4,7,10,13,16-Hexaoxacyclooctadane
			**Go-8**
1	7.671	0.93	Acetic acid ethyl ester
2	7.954	0.10	Acetic acid, hydroxy-, ethyl ester
3	8.482	0.11	Butanal, 3-methyl-
4	9.113	25.52	Ethanol
5	16.073	0.13	2-Propanol, 1-amino-
6	19.023	0.04	Hexane-2,5-diol-D2

7	19.916	0.29	Cyclopentasiloxane, decamethyl-
8	22.826	0.05	1-Butanol, 2-methyl-
9	22.903	0.22	1-Butanol, 3-methyl-
10	24.489	0.09	Ethyl hexanoate
11	27.328	0.37	Disulfide, methyl 2-propenyl
12	27.564	0.14	2-Butanone, 3-hydroxy-
13	27.850	0.08	(1E)-1-(Methyldisulfanyl)-1-propen
14	27.954	0.09	Octanal
15	30.149	0.06	2-Butenal, 3-methyl-
16	31.125	1.26	Cyclohexasiloxane, dodecamethyl-
17	31.41	0.19	Acetic acid, methoxy-, methyl este
18	33.711	0.11	Trisulfide, dimethyl
19	37.454	0.10	Octanoic acid, ethyl ester
20	38.438	0.06	1-Octen-3-ol
21	38.830	0.79	2-Furan-carboxaldehyde
22	39.171	0.18	(E)-l-Allyl-2-(prop-l-en-l-yl)disulfane
23	39.434	0.78	Acetic acid
24	40.103	1.88	Diallyl disulphide
25	40.480	0.65	(Z)-1-Allyl-2(prop-1-en-1-yl)disulfane
26	41.801	0.83	Tetradecamethylcycloheptasiloxane
27	42.545	5.75	Benzaldehyde
28	42.678	1.11	3H-l,2-Dithiole
29	43.750	0.11	Nonanoic acid, ethyl ester
30	43.885	0.85	2,3-Butanediol

31	45.577	0.07	2-Furancarboxaldehyde, 5-methyl-
32	46.761	1.40	Trisulfide, methyl 2-propenyl
33	46.902	0.05	2-Cyclopenten-l-one, 3,5,5-trimethyl-
34	48.069	0.07	2-Propen-l-one, l-cyclohexyl-2-methyl-
35	48.557	0.08	Benzoic acid, methyl ester
36	48.723	0.08	3-Butenoic acid
37	49.640	0.89	Benzeneacetaldehyde
38	50.304	0.28	Silanediol, dimethyl-
39	50.804	0.31	2-Furanmethanol
40	51.189	0.5	Benzoic acid, ethyl ester
41	51.608	0.10	Cystine, TBS 2X
42	51.801	0.42	Butanedioic acid, diethyl ester
43	52.620	0.10	2-Cyclohexene-1,4-dione, 2,6,6-trimethyl-
44	55.063	1.31	3-Vinyl-l,2-dithiacyclohex-4-ene
45	57.173	0.27	Benzoic acid, 2-hydroxy-, methyl ester
46	57.819	0.10	Acetic acid, phenyl-, ethyl ester
47	58.011	1.83	Trisulfide, di-2-propenyl
48	59.433	0.09	Ethylphenyl-N-ethylamine
49	59.755	0.07	Silanediol, dimethyl-
50	60.840	0.58	3-Vinyl-l,2-dithiacyclohex-5-ene
51	61.133	0.17	Dodecanoic acid, ethyl ester
52	62.599	1.49	Benzenemethanol
53	64.438	0.43	Phenylethyl Alcohol
54	66.818	0.15	4H-Pyran-4-one, 3-hydroxy-2,6- dimethyl-

55	67.356	0.11	Ethanone, 1- (lH-pyrrol-2-yl)-
56	68.145	0.09	7-Amino-4-methyl-2-quinolinol
57	68.990	0.26	Phenol
58	70.247	0.58	Benzeneethanol, 2-methoxy-
59	71.485	0.66	Tetradecanoic acid, ethyl ester
60	74.047	0.24	Pentadecanoic acid, ethyl ester
61	76.693	0.07	2-Methoxy-4-[(1E)-l-propenyl]phenol
62	77.068	0.35	Phenol, 4-ethyl-
63	77.945	0.15	5-Methoxy-2,3-dihydrobenzofuran
64	79.288	0.14	Hexadecanoic acid, methyl ester
65	80.992	12.84	Hexadecanoic acid, ethyl ester
66	82.125	0.31	Ethyl 9-hexadecenoate
67	84.589	0.20	2(4H)-Benzofuranone, 5,6,7,7a-tetrahydro-4,4,7a-trimethyl-, (R)-
68	89.732	0.63	Octadecanoic acid, ethyl ester
69	90.528	5.71	Ethyl oleate
70	92.441	17.45	Linoleic acid ethyl ester
71	94.475	3.54	9,12,15-Octadecatrienoic acid, ethyl ester, (Z,Z,Z)-
72	96.981	3.04	1,4,7,10,13,16-Hexaoxacyclooctadecane
			**Go-9**
1	7.675	1.98	Acetic acid ethyl ester
2	7.955	0.11	1,2-Hydrazinedicarboxaldehyde
3	9.112	14.46	Ethanol
4	15.916	0.28	1- Propanol, 2-methyl-
5	17.631	0.42	1- Butanol, 3-methyl-, acetate

6	19.424	0.13	Beta.-D-Ribopyranoside, methyl 2,3,4-tri-O-methyl-
7	19.927	0.25	Cyclopentasiloxane, decamethyl-
8	22.807	0.20	2-Methylbutan-1-ol
9	22.884	1.01	Oxirane, 2 -(1,1-dirnethylethyl) -3-methyl-
10	24.496	0.15	Hexanoic acid, ethyl ester
11	27.544	0.07	2-Butanone, 3-hydroxy-
12	27.956	0.07	Octanal
13	28.223	0.06	Ethyl cis-4-hexenoate
14	30.151	0.09	Pyrazine, 2,5-dimethyl-
15	31.125	1.22	Cyclohexasiloxane, dodecamethyl-
16	31.395	0.47	Ethyl 2-hydroxypropanoate
17	35.501	0.24	Pyrazine, trimethyl-
18	37.460	0.19	Octanoic acid, ethyl ester
19	37.971	0.07	*cis*-Linaloloxide
20	38.38	0.69	2-Furan-carboxaldehyde
21	39.062	1.85	Acetic acid
22	40.015	4.24	2,3,5,6 Tetramethyl pyrazine
23	41.354	0.08	Ethyl octan-2-yl carbonate
24	41.457	0.20	(3E,5E)-Hepta-3,5-dien-2-one
25	41.807	1.81	Cycloheptasiloxane, tetradecamethyl-
26	42.436	0.13	4-Ethyl-2,5,6-Trimethylpyrimidine
27	42.544	0.37	Benzaldehyde
28	43.747	0.10	Nonanoic acid, ethyl ester
29	43.880	0.15	1,3-Butanediol

30	44.077	0.10	2-Hexanol
31	44.379	0.30	l,6-Octadien-3-ol, 3,7-dimethyl-
32	45.407	0.10	Ethyl 3-(methylsulfanyl)propanoate
33	45.571	0.10	1H-Pyrazole, 1,3,5-trimethyl-
34	45.808	0.06	1-Propanone, l-(2-furanyl)-
35	46.078	0.45	l,3-Butanediol
36	46.900	0.09	6-Methyl-3,5-heptadien-2-one
37	47.543	0.05	Cyclohexanol, 1,2-dimethyl-, stereoisomer
38	48.074	0.33	(4E)-2,6-Dimethyl-2,4-heptadiene
39	48.552	0.01	Benzoic acid, methyl ester
40	49.647	0.22	Benzeneacetaldehyde
41	49.810	0.17	Decanoic acid, ethyl ester
42	50.038	0.13	1,3-Cyclohexadiene-1-carboxaldehyde, 2,6,6-trimethyl-
43	50.805	0.65	2-Furanmethanol
44	51.192	0.37	Benzoic acid, ethyl ester
45	51.606	0.18	Benzoic acid, 2,5-bis(trimethylsiloxy)-, trimethylsilyl ester
46	51.805	0.23	Butanedioic acid, diethyl ester
47	53.132	0.18	3-Cyclohexene-1-methanol, .alpha.,.alpha.,4-trimethyl-
48	57.179	0.30	Methyl salicylate
49	57.821	1.02	Benzeneacetic acid, ethyl ester
50	59.433	0.93	Acetic acid, 2-phenylethyl ester
51	59.714	0.06	Silanediol, dimethyl-
52	61.136	0.94	Dodecanoic acid, ethyl ester
53	61.624	0.14	Phenol, 2-methoxy-

54	62.597	0.11	Benzyl alcohol
55	63.105	0.34	Ethyl 3-phenylpropanoate
56	64.439	3.41	Benzeneethanol
57	65.313	0.13	Benzeneacetaldehyde, .alpha.-ethylidene-
58	67.361	0.34	Ethanone, 1-(lH-pyrrol-2-yl)-
59	68.142	0.14	1-Methyl-5-phenyl-4-imidazolin-2-one
60	70.245	2.36	Benzeneethanol, 2-methoxy-
61	71.031	0.07	1,3-Dioxane, 2,4,4,6-tetramethyl-
62	71.485	2.25	Tetradecanoic acid, ethyl ester
63	72.445	0.06	5-Methyl-2-phenyl-2-hexenal
64	72.731	0.04	Hexanoic acid, 5-hydroxy-4-oxo-, methyl ester,
65	72.893	0.04	15-Crown-5
66	73.656	0.07	1H-Pyrrole-2-carboxaldehyde, 5-methyl-
67	74.050	0.25	Undecanoic acid., 2,8-dimethyl-, methyl ester
68	74.762	0.21	Methyl 2,6,10-trimethyltridecanoate
69	76.167	0.06	Methyl 6-oxo-7-(2-oxocyclopentyl)heptanoate
70	76.349	0.14	Pentadecanoic acid, ethyl ester
71	77.063	0.14	*p*-Ethyl-phenol
72	77.945	0.21	2-Methoxy-4-vinylphenol
73	78.584	2.49	Sorbic Acid
74	78.969	0.08	2,4-Hexadienoic acid, (E,E)-
75	79.286	0.94	Hexadecanoic acid, methyl ester
76	80.995	14.65	Hexadecanoic acid, ethyl ester
77	82.123	0.74	Ethyl 9-hexadecenoate

78	84.594	0.11	2(4H)-Benzofuranone, 5,6,7,7a-tetrahydro-4,4,7a-trimethyl-
79	89.733	0.53	Octadecanoic acid, 17-methyl-, methyl ester
80	90.526	5.72	Ethyl (9Z)-9-octadecenoate
81	91.049	0.86	9,12-Octadecadienoic acid (Z,Z)-, methyl ester
82	92.441	19.71	Linoleic acid ethyl ester
83	94.472	3.02	9,12,15-Octadecatrienoic acid, ethyl ester, (Z,Z,Z)-
84	94.625	0.06	21-Krone-7
85	95.517	0.39	3,6,9,12,15-Pentaoxanonadecan-1-ol
86	96.988	2.1	1,4,7,10,13,16-Hexaoxacyclooctadecane
			**Go-10**
1	7.675	0.47	Acetic acid., ethyl ester
2	7.962	0.09	Acetic acid, hydroxy-
3	9.132	25.69	Ethanol
4	16.099	0.30	1-Propanol, 2-methyl-
5	17.557	0.05	1-Butanol, 3-methyl-, acetate
6	19.366	0.06	l,3,4-Thiadiazol-2-amine
7	19.748	0.03	Sulfurous acid, di(2-methyl-4-methoxybutyl) ester
8	19.916	0.10	N-(4'-Chlorophenyl)-8-fluoro-3-methyl-isoalloxazine
9	22.013	0.02	l,3-dioxane-5,5-dimethanol, 2-methyl-
10	22.911	1.30	1-Butanol, 3-methyl-
11	24.487	0.09	Ethyl hexanoate
12	27.568	0.12	2-Butanone, 3-hydroxy-
13	28.221	0.27	Ethyl trans-4-hexenoate
14	29.020	0.03	2-Hexenoic acid, ethyl ester

15	31.127	0.47	Cyclohexasiloxane, dodecamethyl-
16	31.409	0.08	Ethyl (S)-(-)-lactate
17	32.767	0.03	l-Methyl-3-cyclohexene-l-carboxylic acid
18	33.719	0.04	1-Propanol, 3-ethoxy-
19	35.266	0.03	Ethanol, 2-(hexadecyloxy)-
20	35.501	0.06	Pyrazine, trimethyl-
21	37.453	0.14	Octanoic acid, ethyl ester
22	37.979	0.03	2-Furanmethanol, 5-ethenyltetrahydro-.alpha.,.alpha.,5-trimethyl-, cis-
23	38.828	0.20	2-Furan-carboxaldehyde
24	40.015	1.26	Pyrazine, tetramethyl-
25	40.340	0.05	Aloxiprin
26	41.460	0.11	(3E,5E)-Hepta-3,5-dien-2-one
27	41.805	1.56	2,4-Hexadienoic acid, ethyl ester,(2E,4E)-
28	42.433	0.04	4-Ethyl-2,5,6-trimethylpyrimidine
29	42.544	0.16	Benzaldehyde
30	43.749	0.09	Nonanoic acid, ethyl ester
31	44.376	0.10	L-Linalool
32	45.407	0.03	3-(Methylthio)propanoic acid ethyl ester
33	45.569	0.06	2-Furancarboxaldehyde, 5-methyl-
34	46.087	1.07	2, 3-Butanediol
35	46.912	0.03	2-Pentanol
36	47.027	0.05	Undecanoic acid, ethyl ester
37	47.555	0.03	Methoxyacetic acid, 2-tridecyl ester
38	47.716	0.04	Furan, tetrahydro-2,5-dimethyl-

39	48.073	0.16	9-Undecenol, 2,10-dimethyl-
40	49.658	0.10	Oxirane, phenyl-
41	49.809	0.18	Decanoic acid, ethyl ester
42	50.038	0.11	1,3-Cyclohexadiene-1-carboxaldehyde, 2,6,6-trimethyl-
43	50.299	0.15	Silanediol, dimethyl-
44	50.806	0.38	2-Furanmethanol
45	51.191	0.10	Benzoic acid, ethyl ester
46	51.605	0.19	Cyclooctasiloxane, hexadecamethyl-
47	51.804	0.09	Butanedioic acid, diethyl ester
48	53.131	0.06	Beta. Fenchyl alcohol
49	57.176	0.14	Benzoic acid, 2-hydroxy-, methyl ester
50	57.821	0.69	Acetic acid, phenyl-, ethyl ester
51	58.360	0.25	Ethanone, l-(2-furanyl)-
52	59.432	0.38	Acetamide, N-(2-phenylethyl)-
53	59.692	0.03	Silanediol, dimethyl-
54	60.353	0.02	Cyclononasiloxane, octadecamethyl-
55	61.135	1.02	Dodecanoic acid, ethyl ester
56	61.624	0.06	Phenol, 2-methoxy-
57	63.096	0.39	Ethyl 3-phenylpropionate
58	64.438	3.67	Benezeneethanol
59	67.358	0.13	Ethanone, 1-(lH-pyrrol-2-yl)-
60	68.133	0.11	Beta.-Ethylphenethyl alcohol
61	70.246	2.58	Guaiacol, 4-ethyl-
62	71.033	0.05	1,3,7,7-Tetramethyl-9-oxo-2-oxabicyclo[4.4.0]decane

63	71.487	1.93	Tetradecanoic acid, ethyl ester
64	72.448	0.04	(2E)-5-Methyl-2-phenyl-2-hexenal
65	72.724	0.04	18-Crown-6, [2-(l,3,2-dioxaborolan-2-yl)phenyl]-
66	72.898	0.07	6-Ethyltetrazolo[l,5-c]pyrimidin-5(6H)-one
67	74.049	0.23	Ethyl 13-methyl-tetradecanoate
68	74.759	0.14	Pentadecanoic acid, ethyl ester
69	76.168	0.03	(E/Z)-l-Methoxy-2-cyclopropylcyclopropane
70	76.340	0.12	Ethyl 13-methyl-tetradecanoate
71	77.070	0.19	Phenol, 4-ethyl-
72	77.940	0.12	2-Methoxy-4-vinylphenol
73	78.333	1.34	Sorbic acid
74	79.287	0.22	Pentadecanoic acid, 14-methyl-, methyl ester
75	81.014	16.77	Hexadecanoic acid, ethyl ester
76	81.809	0.09	15-Crown-5
77	82.126	0.75	Ethyl 9-hexadecenoate
78	83.749	0.14	12-Crown-4
79	84.599	0.08	2(4H)-Benzofuranone, 5,6,7,7a-tetra-trimethyl-
80	89.735	0.59	Octadecanoic acid, ethyl ester
81	90.535	5.62	Ethyl (9Z)-9-Octadecenoate
82	90.860	0.55	Ethyl oleate
83	91.052	0.37	9,12-Octadecadienoic acid, methyl ester
84	92.460	20.00	Linoleic acid ethyl ester
85	94.473	2.82	9,12,15-Octadecatrienoic acid, ethyl ester, (Z,Z,Z)-
86	95.142	2.28	1,4,7,10,13,16-Hexaoxacyclooctadecane

			**Go-11**
1	7.675	0.71	Acetic acid ethyl ester
2	7.958	0.07	Acetic acid, hydroxy-
3	9.122	10.98	Ethanol
4	15.935	0.18	1 -Propanol, 2-methyl-
5	17.629	0.06	1- Butanol, 3-methyl-, acetate
6	19.420	0.05	Beta.-D-Ribopyranoside, methyl 2,3,4-tri-o-methyl-
7	19.775	0.03	Hexahydro-5H-imidazo[5,1-b:4,3-b'] bisthiazole
8	19.926	0.05	6-Aza-5,7,12,14-tetrathiapentacene
9	22.886	0.56	1-Butanol, 3-methyl-
10	24.495	0.06	Hexanoic acid, ethyl ester
11	27.550	0.06	2-Butanone, 3-hydroxy-
12	27.963	0.02	1, 2, 4-Butanetriol
13	28.226	0.02	Hex-4-enolc acid, ethyl ester
14	31.127	0.37	Cyclohexasiloxane, dodecamethyl-
15	31.397	0.32	Propanoic acid, 2-hydroxy-, ethyl ester, (S)-
16	35.267	0.03	Octadecane, 2-methyl-
17	37.455	0.09	Octanoic acid, ethyl ester
18	37.974	0.03	Ethyl 2-(5-methyl-5-vinyltetrahydrofuran-2-yl)propan-2-yl carbonate
19	38.824	0.37	2-Furan-carboxaldehyde
20	40.009	0.40	Pyrazine, tetramethyl-
21	41.809	0.72	2, 4-Hexadienoic acid, ethyl ester
22	42.540	1.63	Benzaldehyde
23	43.748	0.06	Nonanoic acid, ethyl ester

24	43.878	0.09	2, 3-Butanediol
25	44.075	0.04	Oxirane, (methoxymethyl)-
26	44.377	0.06	Cyclohexene, 1-methyl-4-(l-methylethylidene)-
27	45.569	0.09	2-Furancarboxaldehyde, 5-methyl-
28	45.803	0.02	1-Propanone, 1-(2-furanyl)-
29	46.083	0.21	2,3-Butanediol
30	46.910	0.03	4-Hydroxy-4-methyl-2,5-cyclohexadienone
31	48.069	0.13	9-Undecenol, 2,10-dimethyl-
32	48.65	0.00	(R*,S*)-2-(l-hydroxyethyl)oxirancarboxylate
33	49.645	0.04	Phenyl acetaldehyde
34	49.810	0.14	Decanoic acid, ethyl ester
35	50.036	0.07	1,3-Cyclohexadiene-l-carboxaldehyde, 2,6,6-trimethyl-
36	50.296	0.07	Silanediol, dimethyl-
37	50.802	0.13	2- Furanmethanol
38	51.195	0.11	Benzoic acid, ethyl ester
39	51.608	0.02	Hexadecamethylcyclooctasiloxane
40	51.801	0.44	Butanedioic acid, diethyl ester
41	52.950	0.02	Imidodicarbonic acid, diethyl ester
42	53.135	0.03	Beta. Fenchyl alcohol
43	55.596	0.06	Ethyl 2-Ethylcyclopentaneacetate
44	57.176	0.23	Methyl salicylate
45	57.819	0.39	Benzeneacetic acid, ethyl ester
46	58.358	0.97	1-Butanone, l-(2-furanyl)-
47	59.430	0.27	Acetic acid, 2-phenylethyl ester

48	59.709	0.02	Silanediol, dimethyl-
49	61.137	1.32	Dodecanoic acid, ethyl ester
50	61.617	0.05	Phenol, 2-methoxy-
51	62.597	1.81	Benzyl alcohol
52	63.100	0.05	Ethyl 3-phenylpropionate
53	64.436	1.27	Benzeneethanol
54	65.315	0.08	Benzeneacetaldehyde, .alpha.-ethylidene-
55	65.961	0.05	Trans-.beta.-Ionone
56	67.355	0.08	Ethanone, 1-(lH-pyrrol-2-yl)-
57	69.118	0.10	Tetradecanoic acid, ethyl ester
58	69.549	0.05	Myristic acid, methyl ester
59	70.245	2.71	Benzeneethanol, 2-methoxy-
60	70.929	0.23	Butanedioic acid, hydroxy-, diethy1 ester, (. +-.)-
61	71.490	2.86	Tetradecanoic acid, ethyl ester
62	72.450	0.06	5-Methyl-2-phenyl-2-hexenal
63	72.907	0.13	6-Ethyltetrazolo[l,5-c]pyrimidin-5(6H)-one
64	73.101	0.05	3-Hydroxy-N,N-dimethylpropanamide
65	73.661	0.06	1H-Pyrrole-2-carboxaldehyde, 1-methyl-
66	74.763	0.52	Pentadecanoic acid, ethyl ester
67	76.169	0.14	1-(2-Ethoxyethenyl)cyclopropane
68	76.338	0.17	Pentadecanoic acid, ethyl ester
69	76.836	9.3	Sorbic acid
70	77.058	2.28	2,4-Hexadienoic acid, (E,E)-
71	79.285	0.67	Hexadecanoic acid, methyl ester

72	81.024	16.48	Hexadecanoic acid, ethyl ester
73	82.127	1.07	Ethyl 9-hexadecenoate
74	84.592	0.10	2(4H)-Benzofuranone, 5,6,7,7a-tetrahydro-4,4,7a-trimethyl-
75	84.703	0.08	Methyl 9,12-heptadecadienoate
76	85.441	0.04	Heptadecanoic acid, ethyl ester
77	89.739	0.53	Octadecanoic acid, ethyl ester
78	90.538	7.56	Ethyl oleate
79	91.052	0.73	9,12-Octadecadienoic acid (Z,Z)-,methyl ester
80	92.476	24.10	Linoleic acid ethyl ester
81	94.478	2.61	9,12,15-Octadecatrienoic acid, ethyl ester, (Z,Z,Z)-
82	95.144	1.96	1,4,7,10,13,16-Hexaoxacyclooctadecane
			**Go-12**
1	7.676	0.53	Ethyl Acetate
2	9.119	29.17	Ethanol
3	15.919	0.53	1-Propanol, 2-methyl-
4	17.629	0.07	Ethane, 1,1-diethoxy-
5	19.425	0.06	1-Propene, 1,3,3-trimethoxy-
6	19.926	0.63	Cyclopentasiloxane, decamethyl-
7	22.180	0.06	Oxazole, trimethyl-
8	22.884	2.82	1-Butanol, 3-methyl-
9	24.266	0.09	Furan, 2-pentyl-
10	24.499	0.15	Ethyl hexanoate
11	27.541	0.21	2-Butanone, 3-hydroxy-
12	30.154	0.18	Pyrazine, 2,5-dimethyl-

13	31.125	1.91	Cyclohexasiloxane, dodecamethyl-
14	32.288	0.12	1-Hexanol
15	35.494	0.76	Pyrazine, trimethyl-
16	37.458	0.13	Octanoic acid, ethyl ester
17	38.437	0.07	1-Octen-3-ol
18	38.823	0.39	2 -Furan-carboxaldehyde
19	39.156	0.08	Pyrazine, 2,6-diethyl-
20	40.023	19.71	Pyrazine, tetramethyl-
21	41.459	0.25	(3E,5E)-Hepta-3,5-dien-2-one
22	41.800	1.10	Cycloheptasiloxane, tetradecamethy1-
23	42.433	0.52	2,3,5-Trimethyl-6-ethylpyrazine
24	42.544	0.30	Benzaldehyde
25	43.747	0.06	Nonanoic acid, ethyl ester
26	44.377	0.15	L-Linalool
27	45.409	0.09	Ethyl 3-(methylsulfanyl)propanoate
28	46.088	0.41	1,3-Butanediol
29	46.895	0.08	5-Hepten-2-one, 3-methylene-
30	47.531	0.09	6-Hydroxy-2,6-dimethyl-5,6-dihydro-2H-pyrane
31	47.733	0.16	Propanoic acid, 2-methyl-
32	47.924	0.14	Hydrazine, 1,1-diethyl-
33	48.066	0.33	Hexyl 8-methylnon-6-enoate
34	49.658	0.36	Phenyl acetaldehyde
35	50.034	0.11	1,3-Cyclohexadiene-l-carboxaldehyde, 2,6,6-trimethyl-
36	58.801	0.49	2-Furanmethanol

37	51.192	0.09	(3aS,3bR,9bR,10R,11S,11aS)-10-(benzoyloxy)-2,2-dimethyl-4-[(4-methyl phenyl)sulfonyl]-7,8-bis(trimethylsilyl)- 3a,3b,4,5,9b,10,11,11a-octahydro[l,3]dioxolo[4,5-c]phenanthridin-11-yl benzoate
38	51.605	0.06	Cyclododecasiloxane, tetracosamethyl-
39	51.804	0.19	Butanedioic acid, diethyl ester
40	53.129	0.09	L-.Alpha.-terpineol
41	57.176	0.23	Benzoic acid, 2-hydroxy-, methyl ester
42	57.820	0.40	Benzeneacetic acid, ethyl ester
43	59.431	0.19	Acetamide, N-(2-phenylethyl)-
44	59.730	0.06	Silanediol, dimethyl-
45	61.133	0.28	Dodecanoic acid, ethyl ester
46	62.592	0.20	Benzyl alcohol
47	64.436	3.84	Benzeneethanol
48	67.356	0.36	Ethanone, 1-(1H-pyrrol-2-yl)-
49	68.139	0.20	Benzene, 1,3-diisocyanato-2-methyl
50	68.997	0.11	Phenol
51	70.230	0.17	2(3H)-Furanone, dihydro-5-pentyl-
52	71.481	0.98	Tetradecanoic acid, ethyl ester
53	74.047	0.15	Pentadecanoic acid, ethyl ester
54	74.759	0.12	Alpha.-D-galactopyranoside, methy 1 2,3,4,6-tetra-O-methyl-
55	76.342	0.08	12-Crown-4
56	77.941	0.70	2-Methoxy-4-vinylphenol
57	78.783	0.11	15-Crown-5
58	79.284	0.46	Hexadecanoic acid, methyl ester
59	80.984	10.48	Hexadecanoic acid, ethyl ester

60	82.117	0.22	Ethyl 9-hexadecenoate
61	84.592	0.26	2(4H)-Benzofuranone, 5,6,7,7a-tetrahydro-4,4,7a-trimethyl-
62	89.733	7.4	1,4,7,10,13,16-Hexaoxacyclooctadecane
63	90.524	1.99	Ethyl oleate
64	92.427	7.81	Linoleic acid ethyl ester
65	95.307	0.42	21-Krone-7
			**Go-13**
1	7.676	0.78	Ethyl Acetate
2	7.958	0.18	Ethanol, 2-ethoxy-
3	9.126	22.81	Ethanol
4	16.019	0.15	1-Propanol, 2-methyl-
5	17.588	0.05	1 -Butanol, 3-methyl-, acetate
6	19.920	0.26	Cyclopentasiloxane, decamethyl-
7	21.766	0.03	Pentanoic acid, 4-methyl-, ethyl ester
8	22.819	0.13	1-Butanol, 2-methyl-
9	22.895	0.46	1-Butanol, 3-methyl-
10	24.491	0.19	Hexanoic acid, ethyl ester
11	27.560	0.11	2-Butanone, 3-hydroxy-
12	27.955	0.03	Guanosine
13	30.146	0.12	Pyrazine, 2,5-dimethyl-
14	30.959	0.02	Ethyl heptanoate
15	31.124	0.79	Cyclohexasiloxane, dodecamethyl-
16	31.400	0.23	Ethyl 2-hydroxypropanoate
17	32.282	0.05	1-Hexanol

18	32.471	0.03	10-Methylnonadecane
19	32.758	0.04	(1H-Imidazol-4-yl)oxoacetic acid
20	35.261	0.03	Decane
21	35.498	0.18	Pyrazine, trimethyl-
22	37.454	0.20	Octanoic acid. ethyl ester
23	37.980	0.03	2-(5-Methyl-5-vinyltetrahydro-2-furanyl)-2-propanol
24	38.175	0.04	2-Propen-l-ol, 2-methyl-
25	38.438	0.03	1-Octen-3-ol
26	38.826	0.81	Furfural
27	39.158	0.57	Acetic acid
28	40.013	1.97	Pyrazine, tetramethyl-
29	40.937	0.11	2-Heptanol
30	41.461	0.18	(3E,5E)-Hepta-3,5-dien-2-one
31	41.793	1.08	Cycloheptasiloxane, tetradecamethyl-
32	42.434	0.06	2,3,5-Trimethyl-6-ethylpyrazine
33	42.540	0.16	Benzaldehyde
34	43.746	0.05	Nonanoic acid, ethyl ester
35	43.876	0.29	2,3-Butanediol
36	44.075	0.06	Ether, sec-butyl isopropyl
37	44.379	0.14	Cyclohexene, l-methyl-4-(l-methylethylidene)-
38	45.571	0.19	2-Furancarboxaldehyde, 5-methyl-
39	45.801	0.06	(2S,6S)-Bicyclo[2.2.2]octane-2,6-diol
40	46.078	0.53	1,3-Butanediol
41	46.899	0.07	6-Methyl-3,5-heptadiene-2-one

42	47.026	0.07	Tetradecanoic acid, ethyl ester
43	47.552	0.04	Cyclohexanol, 1,2-dimethyl-, cis-
44	47.907	0.05	6-Nitrohexan-2,3-dione
45	48.070	0.38	9-Undecenol, 2,10-dimethyl-
46	48.654	0.12	1-Cyclohexene-l-propanol, .alpha.,Gamma,2,6,6-pentamethyl-
47	49.652	0.10	Benzeneacetaldehyde
48	49.806	0.11	Decanoic acid, ethyl ester
49	50.037	0.09	1,3-Cyclohexadiene-1-carboxaldehyde, 2,6,6-trimethyl-
50	50.304	0.08	Silanediol, dimethyl-
51	50.803	0.56	2- Furanmethanol
52	51.193	0.11	Benzoic acid. ethyl ester
53	51.373	0.04	2,3,4-Trimethylfuran
54	51.600	0.38	Cyclooctasiloxane, hexadecamethyl-
55	51.800	0.34	Butanedioic acid, diethyl ester
56	52.942	0.02	(-)-Isolongifolol, acetate
57	53.134	0.11	2-(4-Methyl-3-cyclohexen-l-yl)-2-propanol
58	57.175	0.37	Methyl salicylate
59	57.819	0.96	Benzeneacetic acid, ethyl ester
60	58.356	0.05	l-(Furan-2-yl)-4-methylpentan-l-on
61	59.430	0.29	2-Pentanone, 5-phenyl-
62	59.724	0.03	Silanediol, dimethyl-
63	60.349	0.03	Cyclononasiloxane, octadecamethyl-
64	61.134	0.72	Dodecanoic acid, ethyl ester
65	61.615	0.09	Phenol, 2-methoxy-

66	62.598	0.16	Benzyl alcohol
67	63.101	0.22	Ethyl 3-phenylpropionate
68	64.434	1.53	Benzeneethanol
69	65.311	0.15	Benzeneacetaldehyde, .alpha.-ethylidene-
70	65.961	0.09	Trans-.beta.-ionone
71	67.357	0.25	Ethanone, l-(1H-pyrrol-2-yl)-
72	68.995	0.02	Phenol
73	69.550	0.05	Methyl tetradecanoate
74	70.225	0.12	2(3H)-Furanone, dihydro-5-pentyl-
75	70.945	0.06	Butanedioic acid, hydroxy-, diethy1 ester, (. +/-.)-
76	71.484	2.30	Tetradecanoic acid, ethyl ester
77	72.902	0.04	Tetraethyleneglycol monomethylethe
78	73.657	0.07	3-Pyridinemethanol
79	74.047	0.24	Pentadecanoic acid, ethyl ester
80	74.161	0.03	l,6-Dideoxy-2,4-monoethylene-d-altritol
81	74.760	0.15	Octadecanolc acid, 17-methyl-, methyl ester
82	76.160	0.14	l-(2-Ethoxyethenyl)cyclopropane
83	76.337	0.09	Ethyl 13-methyl-tetradecanoate
84	77.943	0.24	4-Vinyl-2-methoxy-phenol
85	78.774	0.06	Hexadecanoic acid, ethyl ester
86	79.026	0.11	Diethyl azelate
87	79.287	0.56	Hexadecanoic acid, methyl ester
88	81.012	20.32	Hexadecanoic acid, ethyl ester
89	82.126	0.61	Ethyl 9-hexadecenoate

90	84.586	0.17	2(4H)-Benzofuranone, 5,6,7,7a-tetrahydro-4,4,7a-trimethyl-, (R)-
91	86.487	2.34	1,4,7,10,13,16-Hexaoxacyclooctadecane
92	89.734	0.70	Octadecanoic acid, ethyl ester
93	90.528	5.61	Ethyl oleate
94	90.888	0.95	5-Hydroxymethylfurfural
95	91.049	0.84	9,12-Octadecadienoic acid (Z,Z)-, methyl ester
96	92.454	20.00	Linoleic acid ethyl ester
97	94.471	3.06	Ethyl 9,12,15-octadecatrienoate
98	95.295	0.25	3,6,9,12,15-Pentaoxanonadecan-1-ol
			**Go-14**
1	7.676	0.75	Ethyl Acetate
2	7.960	0.15	Hydrazine, 2-propenyl-
3	9.110	17.40	Ethanol
4	15.958	0.26	1-Propanol, 2-methyl-
5	17.621	0.09	1-Butanol, 3-methyl-, acetate
6	19.924	0.33	Cyclopentasiloxane, decamethyl-
7	22.182	0.08	Oxazole, trimethyl-
8	22.887	1.46	1-Butanol, 3-methyl- (impure) (CAS
9	24.260	0.08	Furan, 2-pentyl-
10	27.549	0.20	2-Butanone, 3-hydroxy-
11	30.144	0.49	Pyrazine, 2,5-dimethyl-
12	31.125	1.67	Cyclohexasiloxane, dodecamethyl-
13	31.395	0.23	Ethyl 2-hydroxypropanoate
14	32.282	0.12	1-Hexanol

15	35.496	1.49	Pyrazine, trimethyl-
16	37.457	0.13	Octanoic acid, ethyl ester
17	37.972	0.10	2-(5-Methyl-5-vinyltetrahydro-2-furanyl)-2-propanol
18	38.173	0.15	2-Propen-1-ol, 2-methyl-
19	38.437	0.08	1-Octen-3-ol
20	38.824	0.48	2-Furan-carboxaldehyde
21	39.165	0.14	2,3-Dimethyl-5-ethylpyrazine
22	40.024	22.34	Pyrazine, tetramethyl-
23	41.459	0.46	Ethanone, 1-(2-furanyl)-
24	41.795	1.64	Tetradecamethyl-cyclo-hepta-siloxane
25	42.432	0.78	4-Ethyl-2,5,6-trimethylpyrimidine
26	42.540	0.35	Benzaldehyde
27	43.872	0.72	2,3-Butanediol
28	44.059	0.12	2-Hexanol
29	44.376	0.45	Linalool
30	45.581	0.06	2-Furancarboxaldehyde, 5-methyl-
31	46.895	0.25	6-Methyl-3,5-heptadiene-2-one
32	47.151	0.11	Thiazole, 5-methoxy-
33	47.543	0.09	Cyclohexanol, 2,6-dimethyl-
34	47.729	0.16	Propanoic acid, 2-methyl-
35	47.921	0.52	Acetic acid ethyl ester
36	49.633	0.56	Benzeneacetaldehyde
37	50.040	0.44	1,3-Cyclohexadiene-l-carboxaldehyde, 2,6,6-trimethyl-
38	50.319	0.16	Silanediol, dimethyl-

39	50.802	0.78	2-Furanmethanol
40	51.194	0.13	2-Isopropylfuran
41	51.602	0.52	Cyclooctasiloxane, hexadecamethyl-
42	51.814	0.14	4-(Difluoromethyl)pyridine
43	53.135	0.19	3-Cyclohexene-l-methanol, .alpha.,.alpha.,4-trimethyl-
44	57.817	0.44	Benzeneacetic acid, ethyl ester
45	59.426	0.20	B-Phenylethyl acetate
46	59.723	0.07	Silanediol, dimethyl-
47	60.353	0.05	Octadecamethylcyclononasiloxane
48	61.132	0.30	Dodecanoic acid, ethyl ester
49	61.619	0.14	Phenol, 2-methoxy-
50	62.592	0.22	Benzyl alcohol
51	64.435	2.99	Benzeneethanol
52	65.964	0.12	Trans-.beta.-ionone
53	67.355	0.91	Ethanone, 1- (lH-pyrrol-2-yl)-
54	68.137	0.20	4H-l-Benzopyran-2-carboxaldehyde, 4-oxo-
55	68.996	0.27	Phenol
56	70.223	0.17	Thiazole
57	71.483	1.44	Tetradecanoic acid, ethyl ester
58	73.652	0.10	Nicotinyl alcohol
59	74.043	0.14	Pentadecanoic acid, ethyl ester
60	74.759	0.17	Ethyl 13-methyl-tetradecanoate
61	77.947	0.30	2-Methoxy-4-vinylphenol
62	79.286	0.44	Hexadecanoic acid, methyl ester

63	80.983	12.36	Hexadecanoic acid, ethyl ester
64	82.124	0.35	Ethyl 9-hexadecenoate
65	84.587	0.34	2(4H)-Benzofuranone, 5,6,7,7a-tetrahydro-4,4,7a-trimethyl-
66	89.731	0.40	Octadecanoic acid, 17-methyl-, methyl ester
67	90.522	3.46	Ethyl oleate
68	90.890	0.66	2-Furaldehyde, 5-(hydroxymethyl)-
69	92.428	9.37	Linoleic acid ethyl ester
70	94.471	3.82	Ethyl 9,12,15-octadecatrienoate
71	94.705	0.38	21-Krone-7
72	95.029	3.28	1,4,7,10,13,16-Hexaoxacyclooctadecane
			**Go-15**
1	7.675	0.71	Ethyl Acetate
2	7.957	0.18	Propane, 1-ethoxy-
3	9.120	25.98	Ethanol
4	15.992	0.24	1-Propanol, 2-methyl-
5	19.918	0.62	Cyclopentasiloxane, decamethyl-
6	22.185	0.05	Oxazole, trimethyl-
7	22.891	1.09	1-Butanol, 3-methyl-
8	27.549	0.17	2-Butanone, 3-hydroxy-
9	27.952	0.06	Octanal
10	30.147	0.27	Pyrazine, 2,5-dimethyl-
11	31.123	1.58	Cyclohexasiloxane, dodecamethyl-
12	31.392	0.11	Ethyl 2-hydroxypropanoate
13	32.285	0.09	1-Hexanol

14	33.709	0.04	1-Propanol, 3-ethoxy-
15	35.496	0.68	Pyrazine, trimethyl-
16	36.932	0.09	2-Cyclohexen-1-ol
17	37.455	0.14	Octanoic acid, ethyl ester
18	37.979	0.06	Alpha.-Methyl-.alpha.-[4-methyl-3-pentenyl]oxiranemethanol
19	38.176	0.10	2-Propen-l-ol, 2-methyl-
20	38.440	0.08	1-Octen-3-ol
21	38.822	0.48	2 -Furan- carboxaldehyde
22	39.157	0.07	Pyrazine, 2,6-diethyl-
23	39.597	0.55	Acetic acid
24	40.024	17.85	Pyrazine, tetramethyl-
25	41.460	0.20	(3E,5E)-Hepta-3,5-dien-2-one
26	41.800	0.84	Tetradecamethylcycloheptasiloxane
27	42.431	0.51	2,3,5-Trimethyl-6-ethylpyrazine
28	42.538	0.30	Benzaldehyde
29	43.751	0.03	Nonanoic acid, ethyl ester
30	43.874	0.26	2,3-Butanediol
31	44.374	0.22	Linalool
32	45.571	0.12	2-Furancarboxaldehyde, 5-methyl-
33	46.082	0.45	2,3-Butanediol
34	46.892	0.13	6-Methyl-3,5-heptadien-2-one
35	47.150	0.09	Butane, 1-isothiocyanato-
36	47.546	0.09	Cyclohexanol, 1,2-dimethyl-, cis-
37	47.907	0.13	Ethyl-5,5-diethoxy valerate

38	48.070	0.44	2-Allyl-2-methyl-l,3-cyclopentanedione
39	48.468	0.12	Propane-2-13C
40	48.650	0.15	1-Cyclohexene-l-propanol, .alpha.,Gamma,2,6,6-pentamethyl-
41	49.696	0.25	Benzeneacetaldehyde
42	49.799	0.11	Propanoic acid, 2-(phenylmethoxy)-, methyl ester
43	50.039	0.29	1,3-Cyclohexadiene-l-carboxaldehyde, 2,6,6-trimethyl-
44	50.305	0.16	Silanediol, dimethyl-
45	50.801	0.45	2-Furanmethanol
46	51.185	0.20	Benzoic acid, ethyl ester
47	51.606	0.08	Hexadecamethylcyclooctasiloxane
48	51.799	0.60	Butanedioic acid, diethyl ester
49	53.127	0.10	3-Cyclohexene-l-methanol, .alpha.,.alpha.,4-trimethyl-
50	57.174	0.24	Benzoic acid, 2-hydroxy-, methyl ester
51	57.819	0.49	Benzeneacetic acid, ethyl ester
52	58.355	0.16	Bicyclo[4.1.0]heptane, 1-methyl-
53	59.430	0.26	Acetic acid, 2-phenylethyl ester
54	59.703	0.05	Silanediol, dimethyl-
55	61.133	0.36	Dodecanoic acid, ethyl ester
56	61.611	0.12	2-Cyclopenten-l-one, 2,3,4-trimethyl-
57	62.596	0.62	Benzenemethanol
58	63.101	0.12	Ethyl 3-phenylpropanoate
59	64.431	2.47	Benzeneethanol
60	65.959	0.16	Trans-.beta.-Ionone
61	67.360	0.22	Acetylpyrrole

62	68.993	0.13	Phenol
63	70.235	0.28	4-Isopropyl-thiophenol
64	70.925	1.01	Butanedioic acid, hydroxy-, diethy1 ester, (. +/-.)-
65	71.481	1.47	Tetradecanoic acid, ethyl ester
66	73.653	0.06	1-Methyl-2-pyrrolecarboxaldehyde
67	74.047	0.21	Pentadecanoic acid, ethyl ester
68	74.759	0.22	Octadecanoic acid, ethyl ester
69	76.159	0.22	2(1H)-Naphthalenone, octahydro-
70	76.686	0.12	Phenol, 2-methoxy-4-(2-propenyl)-
71	77.937	0.34	2-Methoxy-4-vinylphenol
72	78.777	0.05	12-Crown-4
73	79.287	0.37	Hexadecanoic acid, methyl ester
74	80.985	12.56	Hexadecanoic acid, ethyl ester
75	82.123	0.33	Ethyl 9-hexadecenoate
76	84.587	0.37	Dihydroactinidiolide
77	89.101	1.86	1,4,7,10,13,16-Hexaoxacyclooctadecane
78	89.733	0.45	Octadecanoic acid, 17-methyl-, methyl ester
79	90.524	3.51	Ethyl (9Z)-9-octadecenoate
80	90.908	1.24	5-Hydroxymethylfurfural
81	92.429	10.54	Linoleic acid ethyl ester
82	94.472	1.68	Ethyl 9,12,15-octadecatrienoate
			**Go-16**
1	7.675	0.83	Acetic acid ethyl ester
2	7.956	0.16	1,4-Butanediamine

3	9.110	18.67	Ethanol
4	15.98	0.25	1-Propanol, 2-methyl-
5	17.621	0.09	Alpha.-Hydroperoxy diethyl ether
6	18.554	0.05	Benzene, 1,4-dimethyl-
7	19.922	0.52	Cyclopentasiloxane, decamethyl-
8	22.176	0.09	Oxazole, trimethyl-
9	22.812	0.25	1-Butanol, 2-methyl-
10	22.889	1.34	1-Butanol, 3-methyl-
11	24.261	0.09	Furan, 2-pentyl-
12	24.491	0.22	Ethyl hexanoate
13	26.009	0.14	Ethanedioic acid, diethyl ester
14	27.553	0.20	2-Butanone, 3-hydroxy-
15	30.145	0.50	Pyrazine, 2,5-dimethyl-
16	31.125	1.81	Cyclohexasiloxane, dodecamethyl-
17	31.398	0.19	Propanoic acid, 2-hydroxy-, ethyl ester, (S)-
18	32.285	0.11	1-Hexanol
19	35.496	1.43	Pyrazine, trimethyl-
20	37.458	0.16	Octanoic acid, ethyl ester
21	37.975	0.11	Ethyl 2-(5-methyl-5-vinyltetrahydrofuran-2-yl)propan-2-yl carbonate
22	38.175	0.15	2-Ethoxyethyl nonanoate
23	38.437	0.07	1-Octen-3-ol
24	38.825	0.56	2-Furan-carboxaldehyde
25	39.159	0.21	Pyrazine, 5-ethyl-2,3-dimethyl-
26	40.024	22.03	2,3,5,6 Tetramethyl pyrazine

27	41.461	0.50	Ethanone, 1-(2-furanyl)-
28	41.799	1.37	Cycloheptasiloxane, tetradecamethyl-
29	42.432	0.78	2,3,5-Trimethyl-6-ethylpyrazine
30	42.541	0.39	Benzaldehyde
31	43.744	0.05	Nonanoic acid. ethyl ester
32	43.872	0.39	1,3-Butanediol
33	44.079	0.12	2-Pentanol, 4-methyl-
34	44.376	0.52	L-Linalool
35	45.580	0.09	1,3-Cyclohexadien-1-yl methyl ether
36	46.082	0.41	1,3-Butanediol
37	46.895	0.30	6-Methyl-3, 5-heptadiene-2-one
38	47.145	0.09	Pentane, 2,2'-oxybis-
39	47.540	0.10	3-Acetyl-2,6-heptanedione
40	47.729	0.12	6-Nitrohexan-2,3-dione
41	47.861	0.13	Propanoic acid, 2-methyl-
42	48.068	0.18	Hydrazine, 1,1-diethyl-
42	48.649	0.10	1-Cyclohexene-l-propanol, .alpha.,Gamma,2,6,6-pentamethyl-
43	49.657	0.44	Benzeneacetaldehyde
44	49.803	0.20	Phenyl acetaldehyde
45	50.039	0.49	1,3-Cyclohexadiene-l-carboxaldehyde, 2,6,6-trimethyl-
46	50.305	0.26	Silanediol, dimethyl-
47	50.803	0.78	2-Furanmethanol
48	51.192	0.16	2-(N-Methylamido)furan
49	51.604	0.46	Cyclooctasiloxane, hexadecamethyl-

50	51.800	0.16	Butanedioic acid, diethyl ester
51	53.139	0.02	2-(4-Methyl-3-cyclohexen-l-yl)-2-propanol
52	57.818	0.44	Benzeneacetic acid, ethyl ester
53	59.429	0.19	B-Phenylethyl acetate
54	59.726	0.06	Silanediol, dimethyl-
55	60.347	0.05	Cyclononasiloxane, octadecamethyl-
56	61.134	0.31	Dodecanoic acid, ethyl ester
57	61.618	0.15	Gamma, gamma-dimethylallenyl-ethynyl sulfide
58	62.589	0.19	Benzyl alcohol
59	64.433	2.92	Benzeneethanol
60	65.961	0.12	Trans-beta-ionone
61	67.357	0.91	Ethanone, l-(lH-pyrrol-2-yl)-
62	68.991	0.26	Phenol
63	70.227	0.19	2(3H)-Furanone, dihydro-5-pentyl-
64	71.484	1.64	Tetradecanoic acid, ethyl ester
65	73.653	0.10	1H-Pyrrole-2-carboxaldehyde, 5-methyl-
66	74.046	0.13	Pentadecanoic acid, ethyl ester
67	74.764	0.20	Ethyl 13-methyl-tetradecanoate
68	76.346	0.11	15-Crown-5
69	77.949	0.28	2-Methoxy-4-vinylphenol
70	79.285	0.51	Hexadecanoic acid, methyl ester
71	80.984	13.60	Hexadecanoic acid, ethyl ester
72	82.122	0.38	Ethyl 9-hexadecenoate
73	84.588	0.33	Dihydroactinidiolide

74	89.730	0.44	Octadecanoic acid, ethyl ester
75	90.524	3.68	Ethyl (9Z)-9-Octadecenoate
76	90.885	0.65	5-Hydroxymethylfurfural
77	91.045	1.62	1,4,7,10,13,16-Hexaoxacyclooctadecane
78	92.427	10.07	Linoleic acid ethyl ester
79	94.468	1.71	Ethyl 9,12,15-octadecatrienoate
			**Go-17**
1	7.675	0.91	Acetic acid, ethyl ester
2	7.955	0.16	Hydroxyacetic acid, hydrazide
3	9.132	46.03	Ethanol
4	16.058	0.34	1-Propanol, 2-methyl-
5	17.599	0.09	2-Pentanol
6	19.920	0.17	6-Aza-5,7,12,14-tetrathiapentacene
7	22.906	1.92	1-Butanol, 3-methyl-
8	24.249	0.06	Furan, 2-pentyl-
9	24.486	0.18	Ethyl hexanoate
10	27.564	0.05	2-Butanone, 3-hydroxy-
11	29.765	0.04	Acetic acid, 2-cyano-, 2-(ethoxycarbonyl)hydrazide
12	30.148	0.13	Dimethyl pyrazine
13	31.124	0.95	Cyclohexasiloxane, dodecamethyl-
14	31.401	0.05	2-Pentanol
15	32.286	0.07	1-Hexanol
16	32.472	0.05	Heptacosane
17	32.754	0.05	2-[2-(Methoxy)ethyl]-4(5)-methylimidazole

18	35.262	0.05	Ethanol, 2-(hexadecyloxy)-
19	35.496	0.33	Pyrazine, trimethyl-
20	37.453	0.24	Octanoic acid, ethyl ester
21	38.441	0.06	1-Octen-3-ol
22	38.822	0.28	3-Furaldehyde
23	40.013	5.12	2,3,5,6 Tetramethyl pyrazine
24	41.458	0.16	Ethanone, l-(2-furanyl)-
25	41.793	1.47	Cycloheptasiloxane, tetradecamethyl-
26	42.429	0.13	l,3-Benzodioxole-5-carboxaldehyde
27	42.543	0.21	Benzaldehyde
28	43.747	0.11	Nonanoic acid, ethyl ester
29	43.886	0.20	2,3-Butanediol
30	44.378	0.16	L-Linalool
31	45.407	0.06	Ethyl 3-(methylsulfanyl)propanoate
32	46.082	0.40	1,3-Butanediol
33	46.897	0.08	6-Methyl-3,5-heptadiene-2-one
34	47.024	0.07	Undecanoic acid, ethyl ester
35	47.537	0.04	Cyclohexanol, 1,2-dimethyl-, cis-
36	47.704	0.26	Propanoic acid, 2-methyl-
37	48.064	0.33	8-Methyl-6-nonenoic acid
38	49.636	0.20	Benzeneacetaldehyde
39	49.807	0.19	Decanoic acid, ethyl ester
40	50.033	0.22	1,3-Cyclohexadiene-l -carboxaldehyde, 2,6,6-trimethyl-
41	50.302	0.18	Silanediol, dimethyl-

42	50.802	0.34	2-Furanmethanol
43	51.196	0.08	Benzoic acid, ethyl ester
44	51.599	0.48	Cyclooctasiloxane, hexadecamethyl-
45	51.798	0.14	Butanedioic acid, diethyl ester
46	53.133	0.10	2-(4-Methyl-3-cycl0hexen-l-yl)-2-propanol
47	57.174	0.23	Methyl salicylate
48	57.819	0.48	Benzeneacetic acid, ethyl ester
49	59.428	0.46	Acetic acid, 2-phenylethyl ester
50	59.712	0.05	Silanediol, dimethyl-
51	60.350	0.04	Cyclononasiloxane, octadecamethyl-
52	61.131	0.48	Dodecanoic acid, ethyl ester
53	62.593	0.10	Benzenemethanol
54	64.434	4.88	Benzeneethanol
55	65.955	0.09	3-Buten-2-one, 4-(2,6,6-trimethyl-1-cyclohexen-l-yl)-
56	67.354	0.32	Ethanone, 1-(lH-pyrrol-2-yl)-
57	68.994	0.09	Phenol
58	69.111	0.13	Octadecanoic acid, 17-methyl-, methyl ester
59	70.223	0.14	2(3H)-Furanone, dihydro-5-pentyl-
60	71.482	1.56	Tetradecanoic acid, ethyl ester
61	74.046	0.34	Pentadecanoic acid, ethyl ester
62	74.758	0.27	Methyl 2,12-dimethyltetradecanoate
63	77.943	0.35	4-Vinyl-2-methoxy-phenol
64	79.283	0.33	Pentadecanoic acid, 14-methyl-, methyl ester
65	80.987	11.97	Hexadecanoic acid, ethyl ester

66	82.125	0.28	Ethyl 9-hexadecenoate
67	84.586	0.24	2(4H)-Benzofuranone, 5,6,7,7a-tetrahydro-4,4,7a-trimethyl-
68	89.729	0.23	Octadecanoic acid, 17-methyl-, methyl ester
69	90.518	2.06	Ethyl oleate
70	90.860	1.73	1,4,7,10,13,16-Hexaoxacyclooctadecane
71	92.428	7.92	Linoleic acid ethyl ester
72	94.471	2.27	Ethyl 9,12,15-octadecatrienoate
			**Go-18**
1	6.459	0.17	Acetic acid, methyl ester
2	7.675	2.68	Ethyl acetate
3	7.956	0.36	Acetic acid, hydroxy-
4	8.366	0.11	Butanal, 2-methyl-
5	8.487	0.18	Butanal, 3-methyl-
6	9.116	18.95	Ethanol
7	15.983	0.05	Butanoic acid, 3-hydroxy-, methyl ester
8	17.613	0.08	1- Butanol, 3-methyl-, acetate
9	19.922	0.16	Cyclopentasiloxane, decamethyl-
10	22.806	0.05	2-butoxy-ethanol
11	22.892	0.12	1-Butanol, 3-methyl-
12	24.260	0.03	Furan, 2-pentyl-
13	24.490	0.09	Ethyl hexanoate
14	27.563	0.03	2-Butanone, 3-hydroxy-
15	27.954	0.05	Octanal
16	30.151	0.04	(E)-2,3-Epoxy-1-(methoxymethoxy)octane

17	31.124	0.84	Cyclohexasiloxane, dodecamethyl-
18	31.400	0.13	Ethyl 2-hydroxypropanoate
19	32.471	0.06	Hexadecane, 3-methyl-
20	36.944	0.05	6-Methoxy-2,2-dimethyl-3-chromene
21	37.453	0.22	Octanoic acid, ethyl ester
22	37.973	0.03	Alpha.-Methyl-.alpha.-[4-methyl-3-pentenyl]oxiranemethanol
23	38.093	0.11	1-Pentadecanol
24	38.328	0.05	Propanal, 3-(methylthio)-
25	38.826	5.69	2-Furan-carboxaldehyde
26	39.682	0.29	Acetic acid
27	40.008	0.49	Pyrazine, tetramethyl-
28	41.352	0.07	Decanal
29	41.460	0.12	(3E,5E)-Hepta-3,5-dien-2-one
30	41.792	1.30	Tetradecamethyl-cyclo-hepta-siloxane
31	42.538	0.83	Benzaldehyde
32	43.750	0.06	Nonanoic acid, ethyl ester
33	43.877	0.06	2,3-Butanediol
34	44.374	0.30	L-Linalool
35	45.568	0.31	2-Furancarboxaldehyde, 5-methyl-
36	46.078	0.24	1,3-Butanediol
37	46.892	0.09	6-Methyl-3,5-heptadiene-2-one
38	47.019	0.08	10-Bromodecanoic acid, ethyl ester
39	48.064	0.18	Neopentylidenecyclohexane
40	48.548	0.17	Benzoic acid, methyl ester

41	49.643	0.94	Benzeneacetaldehyde
42	50.031	0.36	Benzene, 1,1'-[l,2-ethanediylbis(oxymethylene)]bis-
43	50.293	0.30	Silanediol, dimethyl-
44	50.800	0.63	2-Furanmethanol
45	51.186	0.28	Benzoic acid, ethyl ester
46	51.599	0.39	Cyclooctasiloxane, hexadecamethyl-
47	51.802	0.06	Succinic acid, ethyl 3-(2-methoxyethyl)nonyl ester
48	52.621	0.06	4-Ketoisophorone
49	52.939	0.04	cis-(-)-2,4a,5,6,9a-Hexahydro-3,5,5,9-tetramethyl(1H)benzocycloheptene
50	53.129	0.10	3-Cyclohexene-1-methanol, .alpha.,.alpha.,4-trimethyl-
51	57.170	0.88	Methyl salicylate
52	57.821	0.10	Benzeneacetic acid, ethyl ester
53	58.913	0.08	Dodecanoic acid, methyl ester
54	59.426	0.11	B-Phenylethyl acetate
55	59.703	0.05	Silanediol, dimethyl-
56	60.352	0.03	Cyclononasiloxane, octadecamethyl-
57	61.134	0.83	Dodecanoic acid, ethyl ester
58	61.619	0.14	Phenol, 2-methoxy-
59	64.431	0.67	Benzeneethanol
60	65.311	0.14	Benzeneacetaldehyde, .alpha.-ethylidene-
61	67.353	0.15	Ethanone, 1-(lH-pyrrol-2-yl)-
62	69.544	0.30	Myristic acid, methyl ester
63	69.926	0.16	1H-Pyrrole-2-carboxaldehyde
64	70.223	0.10	(1E)-1-(Ethylsulfanyl)-1,3-butadiene

65	70.412	0.14	Pentadecanal
66	71.481	2.20	Tetradecanoic acid, ethyl ester
67	72.449	0.11	5-Methyl-2-phenyl-2-hexenal
68	74.053	0.14	Ethyl 13-methyl-tetradecanoate
69	76.332	0.07	Pentadecanoic acid, ethyl ester
70	77.062	0.05	*p*-Ethyl-phenol
71	77.943	0.42	2-Methoxy-4-vinylphenol
72	80.471	0.08	3-Pentanol, 3-ethyl-2-methyl-
73	80.991	14.37	Hexadecanoic acid, methyl ester
74	82.123	0.70	Ethyl 9-hexadecenoate
75	84.589	0.08	2(4H)-Benzofuranone, 5,6,7,7a-tetrahydro-4,4,7a-trimethyl-
76	86.489	0.21	4-Vinyl - phenol
77	88.243	2.9	1,4,7,10,13,16-Hexaoxacyclooctacane
78	89.088	1.13	9-Octadecenoic acid (Z)-, methyl ester
79	89.732	0.39	Octadecanoic acid, ethyl ester
80	90.529	6.44	Ethyl (9Z)-9-Octadecenoate
81	90.866	0.56	(E)-9-Octadecenoic acid ethyl ester
82	91.050	3.83	9,12-Octadecadienoic acid (Z,Z)-, methyl ester
83	92.449	21.27	Linoleic acid ethyl ester
84	94.473	2.12	Ethyl 9,12,15-octadecatrienoate
85	95.143	1.02	Octaethylene glycol monododecyl ether
			**Go-19**
1	7.675	2.32	Acetic acid ethyl ester
2	8.486	0.12	Butanal, 3-methyl-

3	9.105	10.10	Ethanol
4	15.311	0.09	Hexanal
5	15.906	0.08	Butanoic acid, 3-hydroxy-, methyl ester
6	19.926	0.19	Cyclopentasiloxane, decamethyl-
7	22.178	0.12	Oxazole, trimethyl-
8	22.801	0.11	1-Butanol, 2-methyl-
9	22.877	0.40	1-Butanol, 3-methyl-
10	27.539	0.81	Acetoin
11	27.956	0.06	Octanal
12	29.773	0.06	1-Pentanol, 4-methyl-
13	30.138	0.42	Pyrazine, 2,5-dimethyl-
14	31.126	1.27	Cyclohexasiloxane, dodecamethyl-
15	31.392	0.62	Propanoic acid, 2-hydroxy-, ethyl ester, (S)-
16	31.677	0.09	Pyrazine, 2,3-dimethyl-
17	32.286	0.08	1-Hexanol
18	32.488	0.22	Tridecane, 2-methyl-
19	34.081	0.11	4-Methylpentyl 2-methylbutanoate
20	35.273	0.21	Tetradecane
21	35.496	2.21	Pyrazine, trimethyl-
22	36 .934	0 .07	Trans-2-tridecenal
23	37.462	0.06	Octanoic acid, ethyl ester
24	38.100	0.10	Pyrazine, 3-ethyl-2,5-dimethyl-
25	38.477	0.05	1-Octen-3-ol
26	38.821	0.24	2-Furan-carboxaldehyde

27	38.948	1.88	Acetic acid
28	39.160	1.75	Pyrazine, 3-ethyl-2,5-dimethyl-
29	40.054	54.31	2,3,5,6 Tetramethyl pyrazine
30	40.947	0.66	1-Hexanol, 2-ethyl-
31	41.458	0.14	Ethanone, 1-(2-furanyl)-
32	41.803	0.51	Tetradecamethyl-cyclo-hepta-siloxane
33	42.433	2.74	2,3,5-Trimethyl-6-ethylpyrazine
34	42.535	0.56	Benzaldehyde
35	43.749	0.07	Nonanoic acid, ethyl ester
36	43.871	1.44	2,3-Butanediol
37	44.233	0.17	1-Methyl-7,7-dimethyl-2,4-diazabicyclo[4.2.0]octa-l,5-diene-3-one
38	45.190	0.20	Acetic acid, methoxy-, ethyl ester
39	45.570	0.09	2-Furancarboxaldehyde, 5-methyl-
40	45.720	0.20	1,3-Dihydrobenoimidazol-2-one
41	46.075	0.92	2,3-Butanediol
42	46.546	0.06	2,4-Cyclopentadiene-l-carboxaldehyde, 1,2,3,4,5- pentamethyl-
43	46. 895	0.19	6-Methyl-3,5-heptadiene-2-one
44	47.028	0.11	Hexadecanoic acid, ethyl ester
45	47.554	0.37	Cyclohexanol, 2,6-dimethyl-
46	47.728	0.12	Propanoic acid, 2-methyl-
47	47. 915	0. 22	1-Butanol, 4-(l-methylethoxy)-
48	48.067	0.51	Neopentylidenecyclohexane
49	48.535	0.11	1-Benzyl-1-methyl-3-phenylurea
50	48.636	0.20	1-Cyclohexene-l-carboxaldehyde, 2,6,6-trimethyl-

51	49.626	0.54	Benzeneacetaldehyde
52	50.021	0.49	1H-Cycloprop[e]azulene, 1a,2,3,5,6,7,7a,7b-octahydro-l,l,4,7-tetramethyl-, [1aR-(1a.alpha.,7.alpha.,7a .beta.,7b.alpha.)] -
53	50.795	0.11	2-Furanmethanol
54	51.798	0.12	Butanedioic acid, diethyl ester
55	52.940	1.32	(E,Z)-.Alpha.-farnesene
56	53.112	0.20	Butanoic acid, 2-methyl-
57	57.819	0.14	l-(3,5-Dimethyl-2-pyrazinyl)-l-ethanone
58	58.914	0.28	Dimethyl-2,3-dihydro-6,7-5H-cyclopenta[b]pyrazine
59	59.427	0.13	B-Phenylethyl acetate
60	59.720	0.07	Silanediol, dimethyl-
61	61.610	0.24	Ethanone, l-(l-cyclohexen-l-yl)-
62	64.430	1.71	Benzenemethanol
63	65.959	0.47	Trans-.beta.-ionone
64	67.355	0.58	Ethanone, 1-(lH-pyrrol-2-yl)-
65	68.566	0.12	3-Buten-2-ol, 4-(2,6,6-trimethyl-1-cyclohexen-1-yl)-
66	68. 990	0. 15	Phenol
67	70.959	0.29	l,6,10-Dodecatrien-3-ol, 3,7,11-trimethyl-
68	71.480	0.11	Tetradecanoic acid, ethyl ester
69	72.451	0.10	15-Crown-5
70	73.650	0.08	3-Pyridinemethanol
71	76.673	0.08	4-Methoxy-3-methylaniline
72	77.060	0.31	Phenol, 4-ethyl-
73	77.939	1.36	2-Methoxy-4-vinylphenol
74	78.112	0.05	2-Methyl-benzoic acid

75	80.982	0.56	Hexadecanoic acid, ethyl ester
76	84.586	0.76	Dihydroactinidiolide
77	86.486	0.36	Silanol, trimethyl-, nitrate
78	90.526	1.52	1,4,7,10,13,16-Hexaoxacyclooctadecane
			**Go-20**
1	5. 436	0.14	Methane, thiobis-
2	6.462	0.20	Acetic acid, methyl ester
3	7.676	1.73	Ethyl acetate
4	7.953	0.44	Hydroxyacetic acid, hydrazide
5	8.367	0.12	6,8-Dioxabicyclo(3.2.1)octan-3L-ol-3-D1
6	8.487	0.26	Butanal, 3-methyl-
7	9. 107	17.72	Ethanol
8	15. 316	0.20	Hexanal
9	15.899	0.21	1- Propanol, 2-methyl-
10	18.571	0.12	1,4-Dimethylbenzene
11	19.929	0.72	Cyclopentasiloxane, decamethyl-
12	22.798	0.42	1-Butanol, 2-methyl-
13	22.877	0.40	1-Butanol, 3-methyl-
14	24.260	0.13	Furan, 2-pentyl-
15	24.494	0.56	Hexanoic acid, ethyl ester
16	27.538	0.15	2-Butanone, 3-hydroxy-
17	27.953	0.10	Octanal
18	28.657	0.07	7-Oxabicyclo[4.1.0]heptane, 1-methyl-
19	29.772	0.11	1-Pentanol, 4-methyl-

20	30.143	0.19	Pyrazine, 2,5-dimethyl-
21	30.978	0.13	Ethyl heptanoate
22	31.123	2.26	Cyclohexasiloxane, dodecamethyl-
23	31.383	0.10	2,3-Butanediol
24	32.285	0.13	1,5-Pentanediamine
25	32.757	0.16	4-Pyridinol
26	34.198	0.11	1,2-Cyclohexanediol
27	35.270	0.09	Nonane
28	35.490	0.56	Pyrazine, trimethyl-
29	36.936	0.12	E-2-Octenal
30	37.453	0.32	Octanoic acid, ethyl ester
31	37.975	0.10	Ethyl 2-(5-methyl-5-vinyltetrahydrofuran-2-yl)propan-2-yl carbonate
32	38.432	0.23	l-Octen-3-ol
33	38.821	4.93	2-Furan-carboxaldehyde
34	39.153	0.28	2,3-Dimethyl-5-ethylpyrazine
35	40.010	9.22	2,3,5,6 Tetramethyl pyrazine
36	40.939	0.53	1-Hexanol, 2-ethyl-
37	41.454	1.00	Ethanone, 1-(2-furanyl)-
38	41.799	1.72	Tetradecamethylcycloheptasiloxane
39	42.430	0.56	2,3,5-Trimethyl -6-ethylpyrazine
40	42.538	0.36	Benzaldehyde
41	43.745	0.14	Nonanoic acid, ethyl ester
42	44.374	0.51	Cyclohexene, 1- methyl-4-(1-methyl ethylidene)-
43	45.568	0.78	2-Furancarboxaldehyde, 5-methyl-

44	45.795	0.28	1-Propanone, l-(2-furanyl)-
45	46.150	0.41	Dodecane, 5-methyl-
46	46.891	0.57	6-Methyl-3,5-heptadiene-2-one
47	47.538	0.25	Cyclohexanol, 2 ,6-dimethyl-
48	48.074	0.47	Bicyclo[3.1.0]hexan-3-one, 1-acetyl-4-methyl-, [1R-(1.alpha.,4.alpha.,5.alpha.)]-
49	48.555	0.15	Benzaldehyde, 4-methyl-
50	48.634	0.27	1-Cyclohexene- 1-carboxaldehyde, 2,6,6-trimethyl-
51	49.652	0.33	Benzeneacetaldehyde
52	50.034	0.51	1,3-Cyclohexadiene-l-carboxaldehyde, 2,6,6-trimethyl-
53	50.798	1.53	2-Furanmethanol
54	51.193	0.30	Thiophene, 2-methyl-5-(1-methylpropyl)-
55	51.606	0.26	Cyclooctasiloxane, hexadecamethyl-
56	51.814	0.15	Butanedioic acid, diethyl ester
57	53.133	0.27	3-Cyclohexene-l -methanol, .alpha.,.alpha.,4-trimethyl-
58	57.170	3.28	Methyl salicylate
59	57.818	0.56	Benzeneacetic acid, ethyl ester
60	59.690	0.08	Silanediol, dimethyl-
61	60.947	0.09	2-Pentenenitrile, 2-(l-methylethenyi)-, (E)-
62	61.130	0.68	Dodecanoic acid, ethyl ester
63	61.618	0.28	Phenol, 2-methoxy-
64	62.589	0.23	Benzyl alcohol
65	64.429	0.23	Phenylethyl Alcohol
66	65.310	0.27	Benzeneacetaldehyde, .alpha.-ethylidene-
67	65.959	0.19	3-Buten-2-one, 4-(2,6,6-trimethyl-1-cyclohexen-l-yl)-

68	67.354	1.51	Ethanone, 1-(1H-pyrrol-2-yl)-
69	67.642	3.66	Maltol
70	69.547	0.26	Myristic acid, methyl ester
71	71.481	2.26	Tetradecanoic acid, ethyl ester
72	73.658	0.11	3-Pyridinemethanol
73	74.043	0.11	Ethyl 13-methyl-tetradecanoate
74	75.235	0.12	(E)-l-(3-Pyridyl)oct-3-en-l-yne
75	76.339	0.14	Butane, 1,2,3,4-tetramethoxy-
76	77.937	0.27	2-Methoxy-4-vinylphenol
77	79.282	1.79	Hexadecanoic acid, methyl ester
78	80.981	13.60	Hexadecanoic acid, ethyl ester
79	82.115	0.31	Octaethylene glycol monododecyl ether
80	84.577	0.36	Dihydroactinidiolide
81	86.481	0.32	4-Vinylphenol
82	90.521	1.88	Ethyl (9Z)-9-octadecenoate
83	90.905	0.91	5-Hydroxymethylfurfural
84	91.044	1.23	9,12-Octadecadienoic acid (Z,Z)-,methyl ester
85	92.423	7.70	Linolelc acid ethyl ester
86	94.469	3.44	1,4,7,10,13,16- Hexaoxacyclooctadecane
			**Go-21**
1	7.667	0.50	Acetic acid ethyl ester
2	8.477	0.48	Butanal, 3-methyl-
3	9.100	36.94	Ethanol
4	15.229	1.04	Hexanal

5	17.626	0.63	Benzene, ethyl-
6	18.087	0.21	Benzene, 1,4-dimethyl-
7	18.467	0.39	Benzene, 1,3-dimethyl-
8	19.914	0.90	Cyclopentasiloxane, decamethyl-
9	21.145	0.20	1,4-Dimethylbenzene
10	21.295	0.18	N Heptanal
11	22.131	0.17	Cyclohexene, l-methyl-4-(l-methylethenyl)-
12	22.824	0.12	1,1-Dimethylethylamine, N-methoxycarbonyloxy
13	22.905	0.16	1-Butanol, 3-methyl-
14	24.243	0.29	Furan, 2-pentyl-
15	24.482	0.23	Ethyl hexanoate
16	25.729	0.47	Benzene, ethenyl-
17	27.955	0.27	Octanal
18	29.781	0.18	10-Azido-1-decanethiol
19	30.145	0.48	2-Heptenal, (E)-
20	31.122	4.89	Cyclohexasiloxane, dodecamethyl-
21	32.286	0.30	1-Hexanol
22	32.469	0.24	Docosane
23	34.720	0.43	Nonanal
24	35.259	0.17	Methoxyacetic acid, 2-tridecyl ester
25	36.934	0.45	(E)-Oct-2-enal
26	37.451	0.28	Octanoic acid, ethyl ester
27	38.433	0.42	l-Octen-3-ol
28	38.822	3.08	2-Furan-carboxaldehyde

29	40.005	1.70	Pyrazine, tetramethyl-
30	40.853	0.72	2,4-Heptadienal, (E,E)-
31	40.934	0.34	2-Heptanol, 5-methyl-
32	41.457	1.07	Ethanone, 1-(2-furanyl)-
33	41.794	4.69	Cycloheptasiloxane, tetradecamethyl-
34	42.537	1.26	Benzaldehyde
35	43.535	0.41	(6Z)-6-Nonenal
36	43.753	0.21	Nonanoic acid, ethyl ester
37	44.375	0.33	Cyclohexene, l-methyl-4-(l-methylethylidene)-
38	45.570	0.33	2-Furancarboxaldehyde, 5-methyl-
39	46.151	1.69	2,4-Pentanedione, 3-acetyl-
40	46.896	0.42	6-Methyl-3,5-heptadien-2-one
41	47.536	0.74	Cyclohexanol, 2,6-dimethyl-
42	48.068	0.16	Furan, 2-ethenyltetrahydro-2-methyl-5-(5-methyl-l-methylene-4-hexeny 1)-, (2S-trans)-
43	48.638	0.56	1-Cyclohexene-l-carboxaldehyde, 2,6,6-trimethyl-
44	49.636	1.76	Benzeneacetaldehyde
45	50.034	1.19	1,3-Cyclohexadiene-l-carboxaldehyde, 2,6,6-trimethyl-
46	50.283	0.96	Silanediol, dimethyl-
47	50.799	3.65	2-Furanmethanol
48	51.186	0.39	2-Ethyl-5-propylthiophene
49	51.601	1.19	Cyclooctasiloxane, hexadecamethyl-
50	51.839	0.16	Trimethylsilyl ester of furan-3-carboxylic acid
51	52.614	0.32	2-Cyclohexene-l,4-dione, 2,6,6-tri methyl-
52	57.173	0.53	Methyl salicylate

53	59.167	0.47	2,4-Decadienal, (E,E)-
54	59.711	0.19	Silanediol, dimethyl-
55	60.353	0.26	l,l,l,5,7,7,7-Heptamethyl-3,3-bis(trimethylsiloxy)tetrasiloxane
56	60.942	0.21	2-Ethyl-.delta.-(l(2))-bicyclo[4.3 .0]nonene
57	62.592	0.31	Benzyl alcohol
58	64.429	0.71	Phenylethyl alcohol
59	65.955	0.70	Trans-.beta.-ionone
60	67.351	0.65	Ethanone, 1- (lH-pyrrol-2-yl)-
61	68.57	0.34	3-Buten-2-one, 4-(2,2,6-trimethyl-7-oxabicyclo[4.1.0]hept-1-yl)-
62	68.973	0.70	Furyl hydroxymethyl ketone
63	71.482	0.89	Tetradecanoic acid, ethyl ester
64	76.351	0.28	4-Butyl-5-ethylimidazole
65	77.943	0.62	2-Methoxy-4-vinylphenol
66	80.978	2.78	Hexadecanoic acid, ethyl ester
67	81.269	0.38	4H-Pyran-4-one, 2,3-dihydro-3,5-dihydroxy-6-methyl-
68	84.585	1.71	2(4H)-Benzofuranone, 5,6,7,7a-tetrahydro-4,4,7a-trimethyl-, (R)-
69	94.466	6.61	1,4,7,10,13,16-Hexaoxacyclooctadecane
70	95.343	3.32	Benzoic acid, phenylmethyl ester
			**Go-22**
1	7.675	0.78	Acetic acid ethyl ester
2	7.953	0.20	Acetic acld, hydroxy-
3	8.364	0.17	Butanal, 2-methyl-
4	8.485	0.43	Butanal, 3-methyl-
5	9.102	7.54	Ethanol

6	15.283	0.10	Hexanal
7	15.926	0.09	1-Propanol, 2-methyl-
8	19.924	0.32	Cyclopentasiloxane, decamethyl-
9	22.158	0.09	D-Limonene
10	22.802	0.09	1-Butanol, 2-methyl-
11	22.881	0.25	1-Butanol, 3-methyl-
12	24.258	0.32	Furan, 2-pentyl-
13	24.490	0.36	Hexanoic acid, ethyl ester
14	25.574	0.06	Cyclopropane, 1-(1-methylethenyl)-2-(2-methyl-1-propenyl)-, (1R-trans)-
15	27.549	0.11	Ethen-2-d-ol, (E)-
16	27.954	0.13	Octanal
17	30.143	0.33	Pyrazine, 2,5-dimethyl-
18	30.553	0.17	Pyrazine, 2,6-dimethyl-
19	30.968	0.06	Ethyl heptanoate
20	31.123	1.90	Cyclohexasiloxane, dodecamethyl-
21	31.390	0.18	1,2-Propanediol
22	32.476	0.42	Tridecane, 2-methyl-
23	33.740	0.13	Disulfide, dipropyl
24	34.719	0.07	Nonanal
25	35.267	0.16	Tetradecane
26	35.491	1.10	Pyrazine, trimethyl-
27	36.933	0.26	Benzene, 1,4-bis(1,1-dimethylethyl)-
28	37.452	0.58	Octanoic acid, ethyl ester
29	37.974	0.07	2-(5-Methyl-5-vinyltetrahydro-2-furanyl)-2-propanol

30	38.327	0.12	Propanal, 3-(methylthio)-
31	38.433	0.40	1-Octen-3-ol
32	38.822	5.59	2-Furan-carboxaldehyde
33	39.153	0.09	2-Amino-6-ethyl-3-methylpyridine
34	39.534	0.28	Hydrazine, 1,2-dimethyl-, dihydrochloride
35	39.689	0.91	Acetic acid
36	40.007	5.55	2,3,5,6 Tetramethyl pyrazine
37	41.799	1.58	Cycloheptasiloxane, tetradecamethyl-
38	42.535	3.40	Benzaldehyde
39	43.528	0.11	2-Nonenal, (E)-
40	43.746	0.24	Nonanoic acid, ethyl ester
41	43.878	0.11	2,3-Butanediol
42	44.376	1.52	L-Linalool
43	44.929	0.09	Docosane
44	45.571	0.59	3,4,5-Trimethylpyrazole
45	46.080	0.44	2,3-Butanediol
46	46.895	0.24	(3E)-6-Methyl-3,5-heptadien-2-one
47	47.023	0.20	2-Cyclohexen-l-one, 3,5,5-trimethyl-
48	47.551	0.17	Cyclohexanol, 2,6-dimethyl-
49	48.060	0.37	Neopentylidenecyclohexane
50	48.539	0.19	Benzoic acid, methyl ester
51	48.632	0.2	1-Cyclohexene-l-carboxaldehyde, 2,6,6-trimethyl-
52	49.651	2.01	Benzeneacetaldehyde
53	50.028	1.08	1,3-Cyclohexadiene-1-carboxaldehyde, 2,6,6-trimethyl-

54	50.149	0.21	Cyclohexene, 3-methylene-4-vinyl-
55	50.269	0.53	Phenyl acetaldehyde
56	50.796	0.44	2-Furanmethanol
57	51.196	0.31	2-Isopropylimidazole
58	51.360	0.85	Estragole
59	51.603	0.47	Hexadecamethylcyclooctasiloxane
60	51.826	0.25	2-Furancarboxamide, N-methyl-
61	52.619	0.60	2-Cyclohexene-l,4-dione, 2,6,6-trimethyl-
62	52.937	0.29	Gamma-Himachalene
63	53.128	0.43	3-Cyclohexene-l-methanol, .alpha., .alpha.,4-trimethyl-
64	57.167	2.57	Methyl salicylate
65	57.817	0.25	Ethyl 2-phenylacetate
66	58.350	0.19	Bicyclo[4.1.0]heptane, 1-methyl-
67	59.423	0.39	Acetic acid, 2-phenylethyl ester
68	59.715	0.09	Acetophenone, 2-hydroxy-
69	61.133	0.67	Dodecanoic acid, ethyl ester
70	61.618	0.35	Phenol, 2-methoxy-
71	64.429	1.35	Phenylethyl alcohol
72	65.957	0.21	Trans-.beta.-ionone
73	66.809	0.33	2-Methyl-3-methoxy-4H-pyran-4-one
74	67.354	0.19	Ethanone, 1-(1H-pyrrol-2-yl)-
75	69.548	0.24	Myristic acid, methyl ester
76	70.236	1.91	Benzeneethanol, 2-methoxy-
77	70.969	0.12	Nerolidol

78	71.029	0.07	Ethyl methylphosphonate
79	71.481	1.41	Tetradecanoic acid, ethyl ester
80	74.047	0.14	Ethyl 13-methyl-tetradecanoate
81	74.771	0.18	Thiophene, tetrahydro-
82	76.334	0.10	15-Crown-5
83	77.063	0.80	Phenol, 4-ethyl-
84	77.940	1.56	2-Methoxy-4-vinylphenol
85	79.282	1.30	Hexadecanoic acid, methyl ester
86	80.979	5.29	Hexadecanoic acid, ethyl ester
87	82.119	0.46	Ethyl 9-hexadecenoate
88	84.583	0.40	2(4H)-Benzofuranone, 5,6,7,7a-tetr ahydro-4,4,7a-trimethyl-, (R)-
89	86.483	0.69	Benzofuran, 2,3-dihydro-
90	89.087	1.54	7-Octadecenoic acid, methyl ester
91	89.395	2.55	1,4,7,10,13,16-Hexaoxacyclooctadecane
92	90.522	5.66	Ethyl oleate
93	90.855	0.52	15-Crown-5
94	91.041	4.43	9,12-Octadecadienoic acid (Z,Z)-,methyl ester
95	92.431	15.45	Linoleic acid ethyl ester
96	94.470	2.27	Ethyl 9,12,15-octadecatrienoate
			**Go-23**
1	7.672	0.93	Ethyl acetate
2	7.829	0.05	Ethane, 1,1-diethoxy-
3	7.960	0.24	Formic acid., propyl ester
4	9.161	31.9	Ethanol

5	19.915	0.05	*2*,2-Dideuteropropane
6	22.825	0.09	2-Methylbutan-1-ol
7	22.904	0.48	1-Butanol, 3-methyl-
8	25.724	0.03	Benzene, ethenyl-
9	27.565	0.42	2-Butanone, 3-hydroxy-
10	28.791	0.02	Methoxyacetic acid, 3-tetradecyl ester
11	30.955	0.01	Decanoic acid, ethyl ester
12	31.125	0.22	Cyclohexasiloxane, dodecamethyl-
13	31.401	0.16	Propanoic acid, 2-hydroxy-, ethyl ester, (S)-
14	32.289	0.02	1-Hexanol
15	32.462	0.03	Tridecane, 2-methyl-
16	35.252	0.04	Tetradecane
17	36.921	0.02	Benzene, l,3-bis(1,1-dimethylethyl)-
18	37.445	0.29	Octanoic acid, ethyl ester
19	38.818	0.17	2 -Furan -carboxaldehyde
20	39.677	0.19	Acetic acid
21	40.008	0.14	Pyrazine, tetramethyl-
22	40.883	0.05	Ethyl tridecanoate
23	41.803	0.17	Cycloheptasiloxane, tetradecamethyl-
24	42.537	0.23	Benzaldehyde
25	43.741	0.07	Nonanoic acid, ethyl ester
26	43.875	0.10	2,3-Butanediol, [R-(R@,R@)]-
27	44.371	0.07	L-Linalool
28	44.924	0.02	Hentriacontane

29	46.074	0.22	2,3-Butanediol
30	46.904	0.03	Methane, [(1-ethynylcyclohexyl)oxy]methoxy-
31	47.562	0.04	Hexadecane
32	48.060	0.23	Neopentylidenecyclohexane
33	49.634	0.04	Phenyl acetaldehyde
34	49.806	0.49	Decanoic acid, ethyl ester
35	50.014	0.07	1,3-Cyclohexadiene-l-carboxaldehyde, 2,6,6-trimethyl-
36	50.292	0.10	Silanediol, dimethyl-
37	50.798	0.06	2- Furanmethanol
38	51.190	0.05	Benzoic acid, ethyl ester
39	51.807	0.07	Butanedioic acid, diethyl ester
40	52.758	0.02	Ethyl 9-decenoate
41	52.93	0.04	cis-(-)-2,4a,5,6,9a-Hexahydro-3,5,5,9-tetramethyl(lH)benzocycloheptene
42	53.124	0.03	3-Cyclohexene-l-methanol, .alpha.,.alpha.,4-trimethyl-
43	57.172	0.13	Methyl salicylate
44	57.814	0.04	Benzeneacetic acid, ethyl ester
45	58.917	0.04	Dodecanoic acid, methyl ester
46	59.423	0.17	Acetic acid, 2-phenylethyl ester
47	59.734	0.02	Silanediol, dimethyl-
48	61.134	1.45	Dodecanoic acid, ethyl ester
49	64.431	1.27	Benzeneethanol
50	65.959	0.05	3-Buten-2-one, 4-(2,6,6-trimethyl-1-cyclohexen-l-yl)-
51	69.547	0.05	Methyl tetradecanoate
52	70.226	0.05	2(3H)-Furanone, dihydro-5-pentyl-

53	70.959	0.02	l,6,10-Dodecatrien-3-ol, 3,7,11-trimethyl-, (R)-
54	71.486	3.28	Tetradecanoic acid, ethyl ester
55	72.903	0.05	l-(l'-Azetidinyl)-2,6-dimethyl-l-cyclohexene
56	73.259	0.02	12-Crown-4
57	74.044	0.55	Pentadecanoic acid, ethyl ester
58	74.755	0.06	Octadecanoic acid, ethyl ester
59	77.058	0.02	Phenol, 3-ethyl-
60	77.939	0.06	2-(4-Methylphenyl)thiirane
61	79.028	0.06	Diethyl azelate
62	79.284	0.47	Hexadecanoic acid, methyl ester
63	81.035	20.77	Hexadecanoic acid, ethyl ester
64	81.790	0.3	15-Crown-5
65	82.128	1.07	Ethyl 9-hexadecenoate
66	84.578	0.09	2(4H)-Benzofuranone, 5,6,7,7a-tetrahydro-4,4,7a-trimethyl-, (R)-
67	85.440	0.07	Heptadecanoic acid, ethyl ester
68	89.738	0.71	Octadecanoic acid, ethyl ester
69	90.251	0.10	1(2H)-Acenaphthylenone, decahydro-, (2a.alpha. 5a.alpha. 8a.alpha. 8b.beta.)-
70	90.536	5.18	Ethyl oleate
71	90.862	0.55	(E)-9-Octadecenoic acid ethyl ester
72	91.046	0.59	9,12-Octadecadienoic acid (Z,Z)-,methyl ester
73	92.474	20.53	Linoleic acid ethyl ester
74	94.476	2.66	9,12,15-Octadecatrienoic acid, ethyl ester, (Z,Z,Z)-
75	95.146	1.54	1,4,7,10,13,16-Hexaoxacyclooctadecane
76	95.301	0.14	Octaethylene glycol monododecyl ether

			**Go-24**
1	5.261	0.04	Carbon disulfide
2	7.674	0.77	Acetic acid, ethyl ester
3	7.832	0.03	Ethane, 1,2-diethoxy-
4	7.954	0.04	1-Propanamine, 3-methoxy-
5	9.122	8.42	Ethanol
6	15.909	0.02	Carbonic acid, dimethyl ester
7	18.563	0.03	Benzene, 1,4-dimethyl-
8	19.929	0.03	N-(4'-Chlorophenyl)-8-fluoro-3-methyl-isoalloxazine
9	21.771	0.11	Pentanoic acid, 4-methyl-, ethyl ester
10	22.176	0.05	Oxazole, trimethyl-
11	22.876	0.08	1-Butanol, 3-methyl-
12	24.028	0.04	Isothiazole, 3-methyl-
13	24.260	0.03	Furan, 2-pentyl-
14	24.492	0.07	Ethyl hexanoate
15	27.379	0.03	Thiazole, 5-methyl-
16	27.537	0.16	2-Butanone, 3-hydroxy-
17	28.877	0.03	D-Xylose, 3,4,5-tri-o-methyl-
18	30.132	0.10	Pyrazine, *2*,5-dimethyl-
19	31.128	0.13	Cyclohexasiloxane, dodecamethyl-
20	31.676	0.02	Pyrazine, 2,3-dimethyl-
21	32.399	8.44	Allyl isothiocyanate
22	32.901	0.02	Allyl isothiocyanate
23	35.491	0.63	Pyrazine, trimethyl-

24	37.454	0.07	Octanoic acid, ethyl ester
25	38.112	0.21	1-Propene, 3-isothiocyanato-
26	38.819	0.03	2-Furan-carboxaldehyde
27	39.155	0.04	Pyrazine, 3-ethyl-2,5-dimethyl-
28	40.037	12.13	2,3,5,6 Tetramethyl pyrazine
29	41.799	0.29	Cycloheptasiloxane, tetradecamethyl-
30	42.428	0.29	2,3,5-Trimethyl-6-ethylpyrazine
31	42.534	0.11	Benzaldehyde
32	43.742	0.02	Nonanoic acid, ethyl ester
33	43.870	0.59	2,3-Butanediol
34	44.232	0.02	2-Pentanol, 3-methyl-
35	45.575	0.02	1,3-Cyclohexadien-1-yl methyl ether
36	45.712	0.04	l,3-Dihydrobenoimidazol-2-one
37	46.076	0.35	1,3-Butanediol
38	47.413	0.10	Propanoic acid, 2-methyl-
39	47.513	0.06	Acetic acid ethyl ester
40	47.728	0.02	Propanoic acid, 2-methyl-
41	47.913	0.02	Isobutyric acid, ethyl ester
42	48.068	0.03	Hydrazine, 1,1-diethyl-
43	48.541	0.04	Carbamic acid, 2-propenyl-, ethyl ester
44	48.930	0.01	Hydrazine, l-(l-methylethyl)-2-propyl-
45	49.603	0.03	Phenyl acetaldehyde
46	49.809	0.03	Decanoic acid, ethyl ester
47	50.802	0.03	3-Furanmethanol

48	51.181	0.02	Benzoic acid, ethyl ester
49	51.604	0.11	Cyclooctasiloxane, hexadecamethyl-
50	51.797	0.03	Butanedioic acid, diethyl ester
51	52.839	0.52	Butanoic acid, 3-methyl-
52	56.528	0.15	N-Hydroxymethylacetamide
53	57.813	0.18	Benzeneacetic acid, ethyl ester
54	59.746	0.03	Silanediol, dimethyl-
55	61.130	0.17	Dodecanoic acid, ethyl ester
56	63.098	0.60	Ethyl 3-phenylpropionate
57	64.426	0.16	Benzeneethanol
58	65.306	0.06	Benzeneacetaldehyde, .alpha.-ethylidene-
59	68.991	0.16	Phenol
60	69.110	0.34	Tetradecanoic acid, ethyl ester
61	69.375	0.08	5-Methylteytrahydrothiophen-2-one
62	71.48	1.20	Tetradecanoic acid, ethyl ester
63	72.003	0.02	Heptaethylene glycol monododecyl ether
64	74.757	1.99	Pentadecanoic acid, ethyl ester
65	76.667	0.04	4-Methoxy-N-methylbenzenamine.
66	76.813	0.03	12-Crown-4
67	77.354	0.02	Hexaethylene glycol
68	77.937	1.18	2-Methoxy-4-vinylphenol
69	79.284	0.09	Hexadecanoic acid, methyl ester
70	81.017	14.8	Hexadecanoic acid, ethyl ester
71	81.790	0.08	15-Crown-5

72	82.123	0.34	Ethyl 9-hexadecenoate
73	84.571	0.02	2(4H)-Benzofuranone, 5,6,7,7a-tetrahydro-4,4,7a-trimethyl-, (R)-
74	85.434	0.05	Heptadecanoic acid, ethyl ester
75	86.484	0.26	Benzofuran, 2,3-dihydro-
76	87.376	1.94	1,4,7,10,13,16-Hexaoxacyclooctadecane
77	89.743	0.61	Octadecanoic acid, ethyl ester
78	90.550	10.9	Ethyl oleate
79	90.864	0.81	(E)-9-Octadecenoic acid ethyl ester
80	91.049	0.25	9,12-Octadecadienoic acid (Z,Z)-, methyl ester
81	92.485	25.01	Linoleic acid ethyl ester
82	94.479	3.87	9,12,15-Octadecatrienoic acid, ethyl ester, (Z,Z,Z)-
			**Go-25**
1	6.453	0.07	Acetic acid. methyl ester
2	7.667	0.54	Acetic acid, ethyl ester
3	7.945	0.39	Acetic acid, hydroxy-
4	9.105	11.96	Ethanol
5	15.907	0.17	2-Butanol
6	19.923	0.10	Cyclopentasiloxane, decamethyl-
7	21.762	0.04	Pentanoic acid, 4-methyl-, ethyl ester
8	22.873	0.35	1 -Butanol, 3-methyl-
9	24.489	0.16	Hexanoic acid, ethyl ester
10	28.881	0.04	Glycine, N-[S-[5-(acetylamino)-2-hydroxyphenyl]-N-L-Gamma-glutamyl -L-cystein yl]-
11	30.135	0.22	Pyrazine, 2, 5-dimethyl-
12	31.124	0.76	Cyclohexasiloxane, dodecamethyl-

13	31.387	0.04	2,3-Butanediol
14	35.266	0.04	Undecane
15	35.489	0.19	Pyrazine, trimethyl-
16	37.450	0.18	Octanoic acid, ethyl ester
17	37.973	0.03	2-Furanmethanol, 5-ethenyltetrahydro-.alpha.,.alpha.,5-trimethyl-, cis-
18	38.817	0.31	2-Furan-carboxaldehyde
19	40.002	0.62	Pyrazine, tetramethyl-
20	41.448	0.15	Ethanone, l-(2-furanyl)-
21	41.795	1.03	Tetradecamethyl-cyclo-hepta-siloxane
22	42.531	0.12	Benzaldehyde
23	43.743	0.07	Nonanoic acid, ethyl ester
24	43.866	0.07	2,3-Butanediol
25	44.373	0.08	Cyclohexene, l-methyl-4-(l-methylethylidene)-
26	45.555	0.05	1, 3-Cyclohexadien-l-yl methyl ether
27	45.791	0.03	(2S,6S)-Bicyclo[2.2.2]octane-2,6-diol
28	46.070	0.21	2,3-Butanediol
29	46.886	0.06	2-Cyclopenten-l-one, 3,5,5-trimethyl-
30	47.026	0.03	Methyl oligosaccharide
31	47.530	0.05	3-Acetyl-2,6-heptanedione
32	48.068	0.11	(lR,3R,8S)-3-(Methyl)bicyclo[6.1.0]nonan-2-one
33	48.543	0.05	Benzoic acid, methyl ester
34	48.689	0.09	Azetidine, 1-nitroso-
35	49.630	0.18	Benzeneacetaldehyde
36	49.802	0.16	Decanoic acid, ethyl ester

37	50.025	0.20	1,3-Cyclohexadiene-l-carboxaldehyde, 2,6,6-trimethyl-
38	50.795	0.57	2- Furanmethanol
39	51.192	0.08	2-Oxabicyclo[3.1.0]hex-3-ene-4-carboxylic acid, methyl ester
40	51.600	0.31	Hexadecamethylcyclooctasiloxane
41	51.791	0.10	Butanedioic acid, diethyl ester
42	53.126	0.08	Beta. Fenchyl alcohol
43	56.354	0.12	Benzeneacetic acid, methyl ester
44	57.167	0.40	Methyl salicylate
45	57.813	0.23	Acetic acid, phenyl-, ethyl ester
46	58.910	0.09	Dodecanoic acid, methyl ester
47	59.419	0.06	B-Phenylethyl formate
48	59.715	0.05	Silanediol, dimethyl-
49	60.348	0.04	Cyclononasiloxane, octadecamethyl-
50	60.946	0.03	2-Ethyl-.delta.-(1(2))-bicyclo[4.3.0]nonene
51	61.127	0.77	Dodecanoic acid, ethyl ester
52	62.590	0.07	Benzyl alcohol
53	64.427	0.98	Benzeneethanol
54	67.348	0.21	Ethanone, l-(lH-pyrrol-2-yl)-
55	67.820	0.48	4H-Pyran-4-one, 3-hydroxy-2-methyl
56	68.143	0.15	Maltol
57	69.541	0.27	Myristic acid, methyl ester
58	71.480	2.39	Tetradecanoic acid, ethyl ester
59	72.173	0.04	Methyl 13-methyltetradecanoate
60	72.897	0.05	Tetradecanoic acid, 12-methyl-, methyl ester, (S)-

61	74.040	0.36	Pentadecanoic acid, ethyl ester
62	74.754	0.31	Ethyl 13-methyl-tetradecanoate
63	77.939	0.34	2-Methoxy-4-vinylphenol
64	78.779	0.09	Hexadecanoic acid, ethyl ester
65	79.028	0.08	12-Crown-4
66	79.280	2.06	Hexadecanoic acid, methyl ester
67	80.470	0.13	Methyl 9-hexadecenoate
68	80.994	15.43	Hexadecanoic acid, ethyl ester
69	82.118	1.11	Ethyl 9-hexadecenoate
70	83.260	0.09	Phenol, 2,4-bis(1,1-dimethylethyl)
71	84.573	0.15	Dihydroactinidiolide
72	84.710	0.08	15-Crown-5
73	86.478	0.27	Benzofuran, 2,3-dihydro-
74	88.240	3.39	1,4,7,10,13,16-Hexaoxacyclooctadecane
75	89.081	1.11	9-Octadecenoic acid, methyl ester, (E)-
76	89.732	0.74	Octadecanoic acid, ethyl ester
77	90.529	7.55	Ethyl (9Z)-9-octadecenoate
78	90.860	0.84	Ethyl oleate
79	91.046	5.13	9,12-Octadecadienoic acid (Z,Z)-,methyl ester
80	92.459	31.19	Linoleic acid ethyl ester
81	94.472	3.01	9,12,15-Octadecatrienoic acid, ethyl ester, (Z,Z,Z)-
			**Go-26**
1	6.453	0.07	Acetic acid, methyl ester
2	7.666	0.78	Acetic acid ethyl ester

3	7.946	0.25	Formic acid, propyl ester
4	8.478	0.07	Butanal, 3-methyl-
5	9.110	13.10	Ethanol
6	15.935	0.20	1-Propanol, 2-methyl-
7	17.618	0.06	Ethyl 2-cyano-3-oxobutanoate
8	19.920	0.07	6-Aza-5,7,12,14-tetrathiapentacene
9	22.880	1.04	1-Butanol, 3-methyl-
10	24.255	0.04	Furan, 2-pentyl-
11	24.485	0.22	Hexanoic acid, ethyl ester
12	25.737	0.06	Benzene, ethenyl-
13	27.535	0.08	2-Butanone, 3-hydroxy-
14	28.877	0.07	Nonanoic acid, 2,4,6-trimethyl-, ethyl ester, (2S,4R,6R)-(+)-
15	30.133	0.25	Pyrazine, 2,5-dimethyl-
16	30.539	0.03	Pyrazine, 2,6-dimethyl-
17	31.125	0.43	Cyclohexasiloxane, dodecamethyl-
18	31.387	0.05	Ethanol, 2,2'-oxybis-
19	32.274	0.05	1-Hexanol
20	32.468	0.13	Dodecane, 3-methyl-
21	35.262	0.09	Undecane
22	35.489	0.23	Pyrazine, trimethyl-
23	36.936	0.05	l-(2-Pentylphenyl)ethanone
24	37.449	0.25	Octanoic acid, ethyl ester
25	37.970	0.03	2-(5-Methyl-5-vinyltetrahydro-2-furanyl)-2-propanol
26	38.094	0.05	Pyrazlne, 2-ethyl-3,5-dimethyl-

27	38.431	0.05	1-Octen-3-ol
28	38.817	0.60	Furfural
29	39.146	0.03	Pyrazine, 5-ethyl-2,3-dimethyl-
30	39.679	0.08	2-Methyl-l-tetradecene
31	40.004	1.19	2,3,5,6 Tetramethyl pyrazine
32	40.884	0.06	Methyl-4-deoxy-2-o-methyl.beta.1-threo-hex-4-enopyranosid uronate
33	41.798	0.58	Cycloheptasiloxane, tetradecamethyl-
34	42.423	0.08	2,3,5-Trimethyl-6-ethylpyrazine
35	42.531	0.32	Benzaldehyde
36	43.744	0.09	Nonanoic acid, ethyl ester
37	43.866	0.19	1,3-Butanediol
38	44.050	0.09	2-Pentanol, 4-methyl-
39	44.376	0.12	L-Linalool
40	44.929	0.03	Docosane
41	45.529	0.08	1,3-Cyclohexadien-l-yl methyl ether
42	45.796	0.04	1-Propanone, l-(2-furanyl)-
43	46.072	0.36	2,3-Butanediol
44	46.891	0.07	2-Cyclopenten-l-one, 3,5,5-trimethyl-
45	47.025	0.20	Decanoic acid, ethyl ester
46	47.555	0.05	Cyclohexanone, 4-hydroxy-4-methyl-
47	48.063	0.77	Neopentylidenecyclohexane
48	48.259	0.06	1,6-Dideoxy-l-mannitol
49	48.545	0.14	Benzoic acid, methyl ester
50	49.693	0.07	2(3H)-Furanone, dihydro-

51	49.634	0.29	Benzeneacetaldehyde
52	49.801	0.29	Decanoic acid, ethyl ester
53	50.024	0.29	1,3-Cyclohexadiene-l-carboxaldehyde, 2,6,6-trimethyl-
54	50.295	0.18	Silanediol, dimethyl-
55	50.795	0.69	2-Furanmethanol
56	51.183	0.20	Benzoic acid, ethyl ester
57	51.602	0.13	Cyclooctasiloxane, hexadecamethyl-
58	51.800	0.05	Butanedioic acid, diethyl ester
59	52.952	0.14	5-Methylhexanoic acid
60	53.124	0.24	Butanoic acid, 3-methyl-
61	56.354	0.13	Benzeneacetic acid. methyl ester
62	57.171	0.31	Methyl salicylate
63	57.812	0.61	Benzeneacetic acid, ethyl ester
64	58.911	0.14	Dodecanoic acid, methyl ester
65	59.421	0.27	Acetic acid, 2-phenylethyl ester
66	59.738	0.04	Silanediol, dimethyl-
67	60.967	0.02	Methyl 2-hydroxythiazole-4-carboxylate
68	61.128	1.00	Dodecanoic acid, ethyl ester
69	61.607	0.12	Phenol, 2-methoxy-
70	62.585	0.08	Benzenemethanol
71	64.425	2.50	Benzeneethanol
72	65.305	0.19	Benzeneacetaldehyde, .alpha.-ethylidene-
73	65.955	0.12	Trans-.beta.-ionone
74	67.348	0.28	Ethanone, l-(lH-pyrrol-2-yl)-

75	67.836	0.16	4H-Pyran-4-one, 3-hydroxy-2-methyl
76	67.954	0.03	Maltol
77	69.108	0.13	Undecanoic acid, ethyl ester
78	69.539	0.34	Myristic acid, methyl ester
79	70.233	0.27	Benzeneethanol, 2-methoxy-
80	70.959	0.17	l,6,10-Dodecatrien-3-ol, 3,7,11-trimethyl-
81	71.48	2.40	Tetradecanoic acid, ethyl ester
82	72.182	0.10	Methyl 9-methyltetradecanoate
83	72.431	0.04	l,4,7,10,13,16,19-Heptaoxa-2-cycloheneicosanone
84	72.718	0.07	Benzene, 1-butynyl-
85	72.902	0.08	Methyl 13-methyltetradecanoate
86	73.643	0.05	1H-Pyrrole-2-carboxaldehyde, 5-methyl-
87	74.038	1.15	Pentadecanoic acid, ethyl ester
88	76.334	0.09	Ethyl 13-methyl-tetradecanoate
89	77.053	0.13	Phenol, 2-ethyl
90	77.936	0.11	4-Vinyl-2-methoxy-phenol
91	79.279	3.19	Hexadecanoic acid, methyl ester
92	80.466	0.06	Heptaethylene glycol monododecyl ether
93	80.998	16.35	Hexadecanoic acid, ethyl ester
94	82.119	0.62	Ethyl 9-hexadecenoate
95	84.581	0.09	Dihydroactinidiolide
96	86.479	0.11	1,3-Dithiane
97	88.236	2.29	1,4,7,10,13,16-Hexaoxacyclooctadecane
98	89.084	1.07	9-Octadecenoic acid (Z)-, methyl ester

99	89.732	0.56	Octadecanoic acid, ethyl ester
100	90.529	6.70	Ethyl oleate
101	90.86	0.60	(E)-9-Octadecenoic acid ethyl ester
102	91.044	4.57	9,12-Octadecadienoic acid (Z,Z)-,methyl ester
103	92.452	24.43	Linoleic acid ethyl ester
104	94.468	2.60	Ethyl 9,12,15-octadecatrienoate
			**Go-27**
1	5.423	0.03	Methane, thiobis-
2	7.664	1.18	Ethyl acetate
3	7.946	0.11	1, 2-Hydrazinedicarboxaldehyde
4	8.478	0.05	Butanal, 3-methyl-
5	9.120	21.07	Ethanol
6	14.608	0.41	Butanoic acid, 3-methyl-, ethyl ester
7	15.952	0.27	1- Propanol, 2-methyl-
8	17.613	0.10	1-Butanol, 3-methyl-, acetate
9	19.921	0.17	Cyclopentasiloxane, decamethyl-
10	22.023	0.03	Isopentyl 2-methylpropanoate
11	22.805	0.23	1-Butanol, 2-methyl-
12	22.885	1.07	1-Butanol, 3-methyl-
13	24.256	0.02	Furan, 2-pentyl-
14	24.487	0.35	Hexanoic acid, ethyl ester
15	27.371	0.05	Butanoic acid. 2-methyl-, 3-methyl
16	27.548	0.04	2-Propanol
17	27.782	0.03	2-Furylmethyl formate

18	28.872	0.08	4D-Methylhexanoic acid ethyl ester
19	30.133	0.17	Pyrazine, 2,5-dimethyl-
20	30.959	0.03	Ethyl heptanoate
21	31.124	0.44	Cyclohexasiloxane, dodecamethyl-
22	31.390	0.09	Ethyl 2-hydroxypropanoate
23	32.278	0.06	1-Hexanol
24	32.465	0.03	Tetradecane, 2,6,10-trimethyl-
25	32.752	0.08	2-[2-(Methoxy)ethyl]-4(5)-methylim idazole
26	35.256	0.03	Undecane
27	35.487	0.26	Pyrazine, trimethyl-
28	37.449	0.25	Octanoic acid, ethyl ester
29	37.974	0.03	2-Furanmethanol, 5-ethenyltetrahydro-.alpha., .alpha.,5-trimethyl-, trans-
30	38.428	0.04	l-Octen-3-ol
31	38.814	0.31	2-Furan-carboxaldehyde
32	39.151	0.02	2-Amino-6-ethyl-3-methylpyridine
33	40.005	1.98	Pyrazine, tetramethyl-
34	41.451	0.04	(3E,5E)-Hepta-3,5-dien-2-one
35	41.798	0.37	Tetradecamethylcycloheptasiloxane
36	42.427	0.16	2,3,5-Trimethyl-6-ethylpyrazine
37	42.530	0.19	Benzaldehyde
38	43.743	0.09	Nonanoic acid, ethyl ester
39	43.869	0.12	2,3-Butanediol
40	44.072	0.06	4-Heptanol, 2,6-dimethyl-
41	44.371	0.13	Cyclohexene, l-methyl-4-(l-methylethylidene)-

42	45.562	0.11	2-Furancarboxaldehyde, 5-methyl-
43	45.787	0.03	(2S,6S)-Bicyclo[2.2.2]octane-2,6-diol
44	46.074	0.30	2,3-Butanediol
45	46.904	0.03	5-Epi-13-Acetoxy-14,15-di-nor-ent-haliman-18,10.beta.-olide
46	47.021	0.05	Undecanoic acid, ethyl ester
47	47.542	0.03	Cyclohexanone, 4-hydroxy-4-methyl-
48	47.712	0.08	Butane, l-(ethenyloxy)-
49	47.819	0.03	Ether, 2-chloro-l-propyl isopropyl
50	47.918	0.05	Propanoic acid, 2-methyl-
51	48.067	0.31	2-Cyclopenten-l-one, 3,5,5-trimethyl-
52	48.705	0.07	5-Methyloxazolidine
53	49.637	0.15	Benzeneacetaldehyde
54	49.800	0.21	Decanoic acid, ethyl ester
55	50.030	0.13	1,3-Cyclohexadiene-l-carboxaldehyde, 2,6,6-trimethyl-
56	50.289	0.09	Perfluoropropionic acid
57	50.793	0.25	2-Furanmethanol
58	51.183	0.14	Benzoic acid, ethyl ester
59	51.600	0.03	3,4-Dihydroxyphenylglycol
60	51.794	0.43	Butanedioic acid, diethyl ester
61	52.940	0.31	Butanoic acid, 3-methyl-
62	53.12	0.23	Thiazole, 4,5-dihydro-2-methyl-
63	55.591	0.04	Alpha.-D-Xylofuranose, cyclic 1,2:3,5-bis(butylboronate)
64	57.165	0.15	Methyl salicylate
65	57.809	0.59	Benzeneacetic acid, ethyl ester

66	59.420	0.38	Acetic acid, 2-phenylethyl ester
67	61.127	0.83	Dodecanoic acid, ethyl ester
68	61.609	0.08	Phenol, 2-methoxy-
69	62.584	0.09	Benzenemethanol
70	63.058	0.12	Fumaric acid, 2-phenethyl 2,2-dichloroethyl ester
71	64.427	3.12	Benzeneethanol
72	65.305	0.10	Benzeneacetaldehyde, .alpha. -ethylidene-
73	65.948	0.07	3-Buten-2-one, 4-(2,6,6-trimethyl-1-cyclohexen-l-yl)-
74	66.806	0.05	2-Butanone, 4-(1-piperidinyl)-
75	67.347	0.34	Ethanone, 1-(lH-pyrrol-2-yl)-
76	67.815	0.30	Maltol
77	69.106	0.20	Tetradecanoic acid, ethyl ester
78	70. 233	1.60	Benzeneethanol, 2-methoxy-
79	71.017	0.06	12-Crown-4
80	71.482	2.39	Tetradecanoic acid, ethyl ester
81	72.892	0.04	Propanoic acid, ethyl ester
82	73.644	0.08	1H-Pyrrole-2-carboxaldehyde, 1-methyl-
83	74.041	0.82	Pentadecanoic acid, ethyl ester
84	74.755	0.83	Ethyl tridecanoate
85	76.149	0.29	Thiophene, 3-ethyl-
86	76.333	0.14	Pentadecanoic acid, ethyl ester
87	77.058	0.58	Phenol, 4-ethyl-
88	77.344	0.02	15-Crown-5
89	77.742	0.03	l-Benzothiophene-3-carbonitrile, 4,5,6,7-tetrahydro-

90	77.945	0.05	4-Vinyl-2-methoxy-phenol
91	78.77	21.09	Hexadecanoic acid, ethyl ester
92	79.019	0.14	Diethyl azelate
93	79.28	0.22	Hexadecanoic acid, methyl ester
94	82.123	0.60	Ethyl 9-hexadecenoate
95	83.31	0.27	Heptadecanoic acid, ethyl ester
96	84.577	0.11	2(4H)-Benzofuranone, 5,6,7,7a-tetrahydro-4,4,7a-trimethyl-, (R)-
97	89.088	1.80	1,4,7,10,13,16-Hexaoxacyclooctadecane
98	89.731	0.91	Octadecanoic acid, ethyl ester
99	90.534	6.35	Ethyl (9Z)-9-octadecenoate
100	90.862	0.69	(E)-9-Octadecenoic acid ethyl este
101	91.044	0.29	Methyl 10-trans,12-cis-octadecadienoate
102	92.45	17.75	Linoleic acid ethyl ester
103	94.473	3.07	Ethyl 9,12,15-octadecatrienoate
			**Go-28**
1	7.672	0.91	Acetic acid ethyl ester
2	7.948	0.16	1,2-Ethanediol, monoformate
3	8.483	0.08	Butanal, 3-methyl-
4	9.097	7.30	Ethanol
5	14.699	0.12	Butanoic acid, 3-methyl-, ethyl ester
6	15.62	0.10	Tetradecane, 4-methyl-
7	15.795	0.07	Hexadecane, 2,6,10,14-tetramethyl-
8	15.893	0.15	1-Propanol, 2-methyl-
9	16.041	0.12	Pentatriacontane

10	17.132	0.06	Propanedioic acid, acetyl-, diethy1 ester
11	17.572	0.19	Tetracosane, 2,6,10,15,19,23-hexamethyl-
12	18.134	0.13	Octane, 2,2-dimethyl-
13	18.468	0.10	2-(2-Hexyloxyethoxy)ethanol
14	18.740	0.07	1-Butanol, 4-butoxy-
15	19.395	0.10	Hexadecane, 3-methyl-
16	19.763	0.09	9-Methylheptadecane
17	19.915	0.24	Cyclopentasiloxane, decamethyl-
18	20.364	0.05	Heptadecane, 4-methyl-
19	22.371	0.09	Dodecane
20	22.787	0.10	1-Butanol, 2-methyl-
21	22.865	0.42	1-Butanol, 3-methyl-
22	24.251	0.09	Furan, 2-pentyl-
23	24.483	0.30	Ethyl hexanoate
24	27.525	0.19	2-Butanone, 3-hydroxy-
25	27.946	0.04	Octanal
26	28.808	0.16	Tridecane
27	30.127	0.12	Pyrimidine, 4,6-dimethyl-
28	30.952	0.04	Ethyl heptanoate
29	31.117	1.28	Cyclohexasiloxane, dodecamethyl-
30	31.381	0.05	2-Butanol
31	32.272	0.08	1-Pentanol, 4-methyl-
32	32.470	0.10	Tridecane, 2-methyl-
33	35.261	0.23	Tetradecane

34	35.482	0.58	Pyrazine, trimethyl-
35	37.445	0.19	Octanoic acid, ethyl ester
36	38.424	0.19	l-Octen-3-ol
37	38.809	0.38	2-Furan-carboxaldehyde
38	39.146	0.21	2,3-Dimethyl-5-ethylpyrazine
39	40.006	11.54	2,3,5,6 Tetramethyl pyrazine
40	41.794	1.41	Cycloheptasiloxane, tetradecamethyl-
41	42.422	0.41	2,3,5-Trimethyl-6-ethylpyrazine
42	42.528	0.30	Benzaldehyde
43	43.742	0.10	Nonanoic acid, ethyl ester
44	43.860	0.26	2,3-Butanediol
45	44.366	0.11	Cyclofenchene
46	45.569	0.06	2-Furancarboxaldehyde, 5-methyl-
47	45.709	0.07	1,3-Dihydrobenoimidazol-2-one
48	46.063	0.28	2,3-Butanediol
49	46.884	0.12	6-Methyl-3,5-heptadiene-2-one
50	47.019	0.06	Hexadecanoic acid, ethyl ester
51	47.544	0.15	Cyclohexanol, 2,6-dimethyl-
52	48.059	0.23	Ethyl 2-hydroxybutyrate
53	48.641	0.11	(1R,2S,4R)-l,2-Epoxy-p-menth-8-ene
54	49.625	0.51	Benzeneacetaldehyde
55	50.026	0.31	1,3-Cyclohexadiene-l-carboxaldehyde, 2,6,6-trimethyl-
56	50.791	0.54	2-Furanmethanol
57	51.180	0.20	Benzoic acid, ethyl ester

58	51.601	0.19	Hexadecamethylcyclooctasiloxane
59	51.829	0.05	2-Isopropylimidazole
60	52.61	0.09	2,6,6-Trimethyl-2-cyclohexene-l,4-dione
61	52.935	0.04	5-Hydroxy-6-methoxy-8-[(4-amino-l-methylbutyl)amino]quinoline trihydrobromide
62	53.121	0.05	2-(4-Methyl-3-cyclohexen-l-yl)-2-propanol
63	57.805	0.27	Ethylphenyl acetate
64	59.729	0.07	Silanediol, dimethyl-
65	61.126	0.25	Dodecanoic acid, ethyl ester
66	61.607	0.13	Phenol, 2-methoxy-
67	64.421	0.71	Phenylethyl alcohol
68	65.954	0.10	3-Buten-2-one, 4-(2,6,6-trimethyl-1-cyclohexen-l-yl)-
69	67.344	0.37	Ethanone, 1- (lH-pyrrol-2-yl)-
70	67.839	0.20	Maltol
71	70.234	11.23	Guaiacol, 4-ethyl-
72	71.477	0.98	Tetradecanoic acid, ethyl ester
73	74.039	0.29	Pentadecanoic acid, ethyl ester
74	74.758	0.25	Methyl 2,12-dimethyltetradecanoate
75	76.34	0.12	3,6,9,12,15-Pentaoxanonadecan-1-ol
76	77.061	0.78	Phenol, 4-ethyl-
77	77.932	6.35	2-Methoxy-4-vinylphenol
78	78.773	0.06	Octaethylene glycol monododecyl ether
79	79.148	0.47	Sorbic acid
80	79.278	1.66	Hexadecanoic acid, methyl ester
81	80.982	13.62	Hexadecanoic acid, ethyl ester

82	82.117	0.40	Ethyl 9-hexadecenoate
83	84.58	0.27	Dihydroactinidiollde
84	86.487	0.38	Benzofuran, 2,3-dihydro-
85	88.115	2.63	1,4,7,10,13,16-Hexaoxacyclooctacane
86	89.728	0.66	Octadecanoic acid, ethyl ester
87	90.52	5.44	Ethyl oleate
88	91.042	1.25	9,12-Octadecadienoic acid (Z,Z)-,methyl ester
89	92.429	16.02	Linoleic acid ethyl ester
90	94.469	2.86	Ethyl 9,12,15-octadecatrienoate
			**Go-29**
1	8.485	0.82	Butanal, 3-methyl-
2	9.100	1.17	Ethanol
3	15.311	1.91	Hexanal
4	15.899	0.28	1-Propanol, 2-methyl-
5	19.924	1.40	Cyclopentasiloxane, decamethyl-
6	21.154	0.36	2-Heptanone
7	21.307	0.59	N Heptanal
8	22.793	0.31	1-Butanol, 2-methyl-
9	22.871	1.04	1-Pentanol
10	23.295	0.28	2-Hexenal, (E)-
11	23.502	0.28	N-Hydroxymethylacetamide
12	24.256	1.37	Furan, 2-pentyl-
13	25.578	0.93	1,3,6-Octatriene, 3,7-dimethyl-, (E)-
14	25.745	0.48	Benzene, ethenyl-

15	27.952	0.49	Octanal
16	30.141	1.56	2-Heptenal, (E)-
17	31.12	8.18	Cyclohexasiloxane, dodecamethyl-
18	32.276	2.32	1-Hexanol
19	32.476	0.34	Eicosane, 10-methyl-
20	34.457	0.35	2-Nonanone
21	34.722	1.48	Nonanal
22	35.267	0.32	Nonane
23	36.089	0.53	(3Z)-3-Ethyl-2 -methyl-1,3-hexadien
24	36.929	1.21	3-Dodecen-l-al
25	38.429	1.27	1-Octen-3-ol
26	38.812	2.08	2-Furan-carboxaldehyde
27	39.060	0.51	Ethylcyclohex-1-ene
28	40.002	0.97	Pyrazine, tetramethyl-
29	40.837	1.17	2,4-Heptadienal, (E,E)-
30	41.444	1.56	Ethanone, l-(2-furanyl)-
31	41.792	8.05	Tetradecamethylcycloheptasiloxane
32	42.529	2.96	Benzaldehyde
33	43.529	0.62	(E)-Non-2 enal
34	43.865	0.20	Oxirane, (methoxymethyl)-
35	44.368	1.47	Cyclohexene, l-methyl-4-(1-methylethylidene)-
36	45.058	0.56	Orcinol
37	45.566	0.38	2-Furancarboxaldehyde, 5-methyl-
38	46.142	2.73	1-(1'-Hydroxycyclopentyl)-1-hydroxycyclopentane

39	46.893	0.75	2(1H)-Pyridinone, hydrazone
40	47.532	0.80	Cyclohexanol, 2,6-dimethyl-
41	48.635	0.73	1-Cyclohexene-l-carboxaldehyde,6,6-trimethyl-
42	49.084	0.37	(S)(+)-Z-13-Methyl-11-pentadecen-1-ol acetate
43	49.65	1.98	Benzeneacetaldehyde
44	50.026	1.09	1,3-Cyclohexadiene-l-carboxaldehyde, 2,6,6-trimethyl-
45	50.618	7.88	Butanoic acid
46	51.194	0.76	Hexanoic acid
47	51.597	3.18	Cyclooctasiloxane, hexadecamethyl-
48	52.611	0.41	Cyclopentanone, 4,4-dimethyl-2-(l-methylethenyl)-
49	53. 132	0.37	3-Cyclohexene-1-methanol, .alpha.,.alpha.,4-trimethyl-
50	59.162	1.91	2,4-Decadienal, (E,E)-
51	59.706	0.30	Silanediol, dimethyl-
52	60.349	0.45	Cyclononasiloxane, octadecamethyl-
53	64.424	2.32	Benzeneethanol
54	65.957	0.66	3-Buten-2-one, 4-(2,6,6-trimethyl-1-cyclohexen-l-yl)-
55	67.35	0.76	Ethanone, 1- (lH-pyrrol-2-yl)-
56	77.931	1.05	2-Methoxy-5-vinylphenol
57	84.574	1.26	2(4H)-Benzofuranone, 5,6,7,7a-tetrahydro-4,4,7a-trimethyl-, (R)-
58	95.535	20.44	1,4,7,10,13,16-Hexaoxacyclooctadecane
			**Go-30**
1	7.947	0.15	2-Pentanamine
2	8.478	0.14	1,2-Ethanediamine, N,N'-dimethyl-
3	9.095	9.62	Ethanol

4	15.907	1.11	1-Propanol, 2-methyl-
5	19.921	0.35	Cyclopentasiloxane, decamethyl-
6	22.173	0.18	Oxazole, trimethyl-
7	22.796	0.93	1-Butanol, 2-methyl-
8	22.875	3.16	1-Butanol, 3-methyl-
9	24.253	0.08	Furan, 2-pentyl-
10	25.732	1.05	Benzene, ethenyl-
11	27.531	1.01	2-Butanone, 3-hydroxy-
12	27.948	0.07	2-Methylcyclopropylmethanol
13	30.133	0.10	Pyrazine, 2,6-dimethyl-
14	31.123	1.86	Cyclohexasiloxane, dodecamethyl-
15	32.278	0.15	1-Hexanol
16	32.473	0.09	Methoxyacetic acid, 2-tridecyl ester
17	35.267	0.07	Undecane, 2-methyl-
18	35.486	0.77	Pyrazine, trimethyl-
19	38.429	0.15	l-Octen-3-ol
20	38.813	0.21	2-Furan-carboxaldehyde
21	39.151	0.46	Pyrazine, 5-ethyl-2,3-dimethyl-
22	40.032	51.59	2,3,5,6 Tetramethyl pyrazine
23	41.449	0.19	Ethanone, 1- (2-furanyl)-
24	41.792	2.36	Tetradecamethylcycloheptasiloxane
25	42.422	2.47	2,3,5-Trimethyl-6-ethylpyrazine
26	43.867	0.25	2,3-Butanediol
27	44.225	0.13	4H-Pyrido[l,2-a]pyrimidin-4-one, 6,7,8,9-tetrahydro-6-methyl-

28	44.368	0.16	L-Linalool
29	45.708	0.14	1H-l,2J3-Benzotriazol-4-amine
30	46.067	1.64	2,3-Butanediol
31	46.270	0.21	Propane, 1,1'-oxybis 2-chloro-
32	46.537	0.31	Pyrazine, trimethylpropyl-
33	47.542	0.09	Oxalic acid, hexyl neopentyl ester
34	49.635	0.24	Phenyl acetaldehyde
35	50.028	0.09	Acetamide, N-(2-pyridinylmethyl)-, conjugate monoacid
36	50.791	0.34	2- Furanmethanol
37	51.194	0.22	Methyl l-methyl-2-propyl-l-cyclopropene-3-carboxylate
38	51.598	0.67	Cyclooctasiloxane, hexadecamethyl-
39	51.828	0.08	2-Furancarboxamide, N-methyl-
40	57.164	1.74	Methyl salicylate
41	59.696	0.09	Silanediol, dimethyl-
42	60.350	0.07	Cyclononasiloxane, octadecamethyl-
43	60.416	0.09	2-Butanone, 4-(2,6,6-trimethyl-l-cyclohexen-l-yl)-
44	61.599	0.17	Phenol, 2-methoxy-
45	64.420	4.27	Benzeneethanol
46	70.950	0.08	1,6-Octadiene, 7-methyl-3-methylene-
47	71.478	0.08	Pentadecanoic acid, ethyl ester
48	76.346	0.14	3,4-Pyridinediamine
49	77.928	7.20	2-Methoxy-4-vinylphenol
50	80.973	0.61	Hexadecanoic acid, ethyl ester
51	84.577	0.34	2(4H)-Benzofuranone, 5,6,7,7a-tetrahydro-4,4,7a-trimethyl-, (R)-

52	86.472	1.83	4-Vinylphenol
53	92.411	0.41	1,4,7,10,13,16-Hexaoxacyclooctadecane
			**Go-31**
1	7.673	1.31	Acetic acid ethyl ester
2	7.956	0.22	2-Methyl-l,3-dioxolan-4-one
3	9.129	26.62	Ethanol
4	16.101	0.11	Ethanol, 2-ethoxy-
5	19.912	0.14	N-(4'-Chlorophenyl)-8-fluoro-3-methyl-isoalloxazine
6	22.906	0.26	Oxirane, 2-(1,1-dimethylethyl)-3-methyl-
7	24.238	0.08	Furan, 2-pentyl-
8	24.478	0.66	Hexanoic acid, ethyl ester
9	27.569	0.07	Ethanol, 2-(vinyloxy)-
10	27.947	0.05	1,3-Dioxolane, 2-methyl-
11	28.799	0.06	Methoxyacetic acid, 2-tridecyl ester
12	29.787	0.12	1-Butanol
13	30.948	0.09	Heptanoic acid, ethyl ester
14	31.121	0.85	Cyclohexasiloxane, dodecamethyl-
15	31.400	0.12	Ethyl 2-hydroxypropanoate
16	32.280	0.14	1-Hexanol
17	32.464	0.13	Dodecane, 3-methyl-
18	34.068	0.05	Butanoic acid, 3-methyl-, hexyl ester
19	35.258	0.10	Nonadecane
20	35.492	0.05	Pyrazine, trimethyl-
21	36.928	0.06	(E)-l-(Methoxymethoxy)-l-octene-3-ol

22	37.445	0.43	Octanoic acid, ethyl ester
23	38.426	0.07	1-Octen-3-ol
24	38.813	0.71	Furfural
25	39.676	0.08	(4E)-4-Methyl-4-decene
26	40.000	0.33	Pyrazine, tetramethyl-
27	40.877	0.17	Ethyl tridecanoate
28	41.792	1.08	Cycloheptasiloxane, tetradecamethyl-
29	42.529	0.36	Benzaldehyde
30	43.738	0.23	Nonanoic acid, ethyl ester
31	44.373	0.11	Cyclohexene, l-methyl-4-(l-methylethylidene)-
32	44.921	0.05	Methoxyacetic acid, 4-tridecyl ester
33	45.573	0.11	3,5-Octadien-2-one
34	46.070	0.18	2,3-Butanediol
35	46.885	0.20	6-Methyl-3,5-heptadiene-2-one
36	47.019	0.26	Heptadecanoic acid, ethyl ester
37	47.541	0.13	Cyclohexanol, 2,6-dimethyl-
38	48.059	0.74	Neopentylidenecyclohexane
39	48.628	0.14	1-Cyclohexene-l-carboxaldehyde, 2,6,6-trimethyl-
40	49.628	0.21	Phenyl acetaldehyde
41	49.799	0.30	Decanoic acid, ethyl ester
42	50.021	0.43	1,3-Cyclohexadiene-l-carboxaldehyde, 2,6,6-trimethyl-
43	50.291	0.33	Silanediol, dimethyl-
44	50.791	0.26	2- Furanmethanol
45	51.18	0.21	Benzoic acid, ethyl ester

46	51.599	0.34	Hexadecamethylcyclooctasiloxane
47	51.803	0.13	Butanedioic acid, diethyl ester
48	52.933	0.27	Gamma-Himachalene
49	53.124	0.07	1,4-Cyclohexadiene, l-methyl-4-(l-methylethyl)-
50	57.166	0.85	Methyl salicylate
51	57.804	0.17	Benzeneacetic acid, ethyl ester
52	59.152	0.09	2,4-Decadienal, (E,E)-
53	59.702	0.04	Silanediol, dimethyl-
54	60.348	0.05	Cyclopentadecasiloxane, triacontamethyl-
55	61.127	1.37	Dodecanoic acid, ethyl ester
56	61.600	0.17	Gamma, gamma-Dimethylallenyl -
57	64.418	0.40	Benzeneethanol
58	65.953	0.32	3-Buten-2-one, 4-(2,6,6-trimethyl-1-cyclohexen-l-yl)-
59	66.803	0.15	Pentanoic acid, 3-acetyl-4-oxo-, ethyl ester
60	67.348	0.33	Ethanone, 1-(lH-pyrrol-2-yl)-
61	68.566	0.10	3-Buten-2-one, 4-(2,2,6-trimethyl-7-oxabicyclo[4.1.0]hept-l-yl)-
62	69.542	0.08	Myristic acid, methyl ester
63	70.219	0.18	2(3H)-Furanone, dihydro-5-pentyl-
64	70.955	0.15	l,6,10-Dodecatrien-3-ol, 3,7,11-trimethyl-
65	71.480	3.65	Tetradecanoic acid, ethyl ester
66	72.448	1.82	1,4,7,10,13,16-Hexaoxacyclooctadecane
67	74.041	0.43	Ethyl 13-methyl-tetradecanoate
68	74.758	0.11	Ethyl 15-methyl-hexadecanoate
69	76.335	0.20	Pentadecanoic acid, ethyl ester

70	77.932	0.86	2-Methoxy-4-vinylphenol
71	79.019	0.16	Diethyl azelate
72	79.280	0.66	Hexadecanoic acid, methyl ester
73	80.995	20.81	Hexadecanoic acid, ethyl ester
74	82.121	0.87	Ethyl 9-hexadecenoate
75	84.576	0.53	2(4H)-Benzofuranone, 5,6,7,7a-tetrahydro-4,4,7a-trimethyl-, (R)-
76	86.473	0.26	Benzofuran, 2,3-dihydro-
77	89.730	0.51	Octadecanoic acid. ethyl ester
78	90.524	4.03	(E)-9-Octadecenoic acid ethyl ester
79	91.040	0.87	9,12-Octadecadienoic acid, methyl ester
80	92.435	17.12	Linoleic acid ethyl ester
81	94.469	3.41	9,12,15-Octadecatrienoic acid, ethyl ester, (Z,Z,Z)-
			**Go-32**
1	6.459	0.12	Acetic acid, methyl ester
2	7.674	1.99	Acetic acid ethyl ester
3	7.950	0.18	1,2-Hydrazinedicarboxaldehyde
4	8.363	0.09	Butanal, 2-methyl-
5	8.485	0.18	Butanal, 3-methyl-
6	9.103	6.36	Ethanol
7	15.282	0.03	DL-Cystathionine
8	15.922	0.10	1-Propanol, 2-methyl-
9	17.620	0.20	1-Butanol, 3-methyl-, acetate
10	19.922	0.11	Cyclopentasiloxane, decamethyl-
11	22.874	0.37	1-Butanol, 3-methyl-

12	24.256	0.08	Furan, 2-pentyl-
13	24.488	0.25	Hexanoic acid., ethyl ester
14	25.559	0.05	1,3,6-Octatriene, 3,7-dimethyl-, (E)-
15	27.536	0.07	2-Butanone, 3-hydroxy-
16	30.143	0.06	(+-)-5-Methyl-2 -hexanol
17	30.956	0.03	Heptanoic acid, ethyl ester
18	31.122	0.58	Cyclohexasiloxane, dodecamethyl-
19	31.382	0.16	Ethyl 2-hydroxypropanoate
20	32.470	0.10	Eicosane, 10-methyl-
21	35.259	0.04	Decane
22	36.920	0.09	Benzene, 1,3-bis(l,1-dimethylethyl)-
23	37.447	0.29	Octanoic acid, ethyl ester
24	38.426	0.04	1-Octen-3-ol
25	38.815	3.50	2-Furan-carboxaldehyde
26	39.379	0.65	Acetic acid
27	39.999	0.15	2,3,5,6 Tetramethyl pyrazine
28	40.885	0.21	Ethyl 13-methyl-tetradecanoate
29	41.793	0.89	Tetradecamethylcycloheptasiloxane
30	42.526	0.53	Benzaldehyde
31	43.739	0.08	Nonanoic acid, ethyl ester
32	43.860	0.63	2,3-Butanediol, [R-(R@,R@)]-
33	44.369	0.42	l,6-Octadien-3-ol, 3,7-dimethyl-
34	44.924	0.04	Methoxyacetlc acid, 4-tetradecyl ester
35	45.174	0.04	Acetic acid, methoxy-, ethyl ester

36	45.542	0.14	2-Furancarboxaldehyde, 5-methyl-
37	46.062	0.30	2,3-Butanediol
38	46.893	0.08	6-Methyl-3,5-heptadiene-2-one
39	47.021	0.23	Undecanoic acid, ethyl ester
40	47.426	0.02	Benzonitrile
41	48.059	0.63	Cyclopropane, ethyl(l,2,2-trimethylpropylidene)-, (Z)-
42	48.544	0.04	Benzoic acid, hydrazide
43	48.640	0.09	1-Cyclohexene-l-carboxaldehyde, 2,6,6-trimethyl-
44	49.639	0.35	Benzeneacetaldehyde
45	49.795	0.28	Decanoic acid, ethyl ester
46	50.028	0.35	1,3-Cyclohexadiene-l-carboxaldehyde, 2,6,6-trimethyl-
47	50.276	0.17	Silanediol, dimethyl-
48	50.789	0.68	2-Furanmethanol
49	51.179	0.23	Benzoic acid, ethyl ester
50	51.598	0.30	Cyclooctasiloxane, hexadecamethyl-
51	51.791	0.22	Butanedioic acid, diethyl ester
52	52.926	0.04	cis-(-)-2,4a,5,6,9a-Hexahydro-3,5,5,9-tetramethyl(lH)benzocycloheptene
53	53.122	0.10	Beta. Fenchyl alcohol
54	57.162	1.51	Methyl salicylate
55	57.807	0.16	Benzeneacetic acid, ethyl ester
56	58.904	0.06	Dodecanoic acid, methyl ester
57	59.418	0.30	Acetic acid, 2-phenylethyl ester
58	59.708	0.05	Silanediol, dimethyl-
59	59.848	0.05	2-Buten-l-one, l-(2,6,6-trimethyl-1,3-cyclohexadien-l-yl)-, (E)-

60	61.127	0.90	Dodecanoic acid, ethyl ester
61	61.607	0.12	Phenol, 2-methoxy-
62	62.582	0.11	Benzenemethanol
63	64.420	1.94	Benzeneethanol
64	65.305	0.07	Benzeneacetaldehyde, .alpha.-ethylidene-
65	65.949	0.14	3-Buten-2-one, 4-(2,6,6-trimethyl-1-cyclohexen-l-yl)-
66	67.339	0.17	Ethanone, l-(lH-pyrrol-2-yl)-
67	69.543	0.18	Myristic acid, methyl ester
68	70.215	0.18	4-Pentylbutan-4-olide
69	71.017	0.06	2,5,5,8A-Tetramethyloctahydro-7H-chromen-7-one
70	71.480	2.62	Tetradecanoic acid, ethyl ester
71	74.044	0.15	Pentadecanoic acid, ethyl ester
72	76.330	0.11	Ethyl 13-methyl-tetradecanoate
73	77.932	0.38	2-Methoxy-4-vinylphenol
74	79.278	1.77	Hexadecanoic acid, methyl ester
75	81.006	21.74	Hexadecanoic acid, ethyl ester
76	82.121	0.69	Ethyl 9-hexadecenoate
77	84.575	0.22	Dihydroactinidiolide
78	86.476	0.16	4-Vinylphenol
79	89.087	0.59	9-Octadecenoic acid, methyl ester
80	89.731	0.76	Octadecanoic acid, ethyl ester
81	90.526	6.48	Ethyl (9Z)-9-octadecenoate
82	90.863	0.62	Ethyl oleate
83	91.045	2.82	9,12-Octadecadienoic acid (Z,Z)-, methyl ester

84	92.451	27.11	Linoleic acid ethyl ester
85	94.472	2.90	Ethyl 9,12,15-octadecatrienoate
86	96.981	1.88	1,4,7,10,13,16-Hexaoxacyclooctadecane
			**Go-33**
1	7.671	0.68	Ethyl Acetate
2	7.956	0.08	1,2-Hydrazinedicarboxaldehyde
3	8.366	0.03	Butanal, 2-methyl-
4	8.486	0.06	Butanal, 3-methyl-
5	9.165	36	Ethanol
6	21.759	0.09	Lyxopyranoside, methyl 2,3,4-tri-0-methyl-, .beta.-D-
7	22.804	0.13	2-Methylbutan-l-ol
8	22.885	0.42	1-Butanol, 3-methyl-
9	24.481	0.07	Hexanoic acid, ethyl ester
11	27.544	0.05	2-Butanone, 3-hydroxy-
11	31.121	0.34	Cyclohexasiloxane, dodecamethyl-
12	31.386	0.45	Ethyl (s)-(-)-lactate
13	32.750	0.03	(1H-Imidazol-4-yl)Oxoacetic acid
14	37.444	0.12	Octanoic acid, ethyl ester
15	38.812	0.44	2-Furan-carboxaldehyde
16	39.999	0.15	Pyrazine, tetramethyl-
17	40.093	0.08	Diallyl disulphide
18	41.793	0.62	Tetradecamethyl-cyclo-hepta-siloxane
19	42.527	0.20	Benzaldehyde
20	43.740	0.02	Nonanoic acid, ethyl ester

21	43.861	0.22	2,3-Butanediol
22	44.063	0.03	2-Hexanol
23	44.372	0.02	Hexan-2-ol
24	45.558	0.11	2-Furancarboxaldehyde, 5-methyl-
25	46.065	0.49	2,3-Butanediol
26	46.884	0.08	6-Methyl-3, 5- heptadiene-2-one
27	47.013	0.04	Ethyl 13-methyl-tetradecanoate
28	48.057	0.11	Neopentylidenecyclohexane
29	48.542	0.02	Benzoic acid, methyl ester
30	48.644	0.04	2-Acetoxybornane-3-ol
31	49.599	0.06	Phenyl acetaldehyde
32	49.800	0.11	Decanoic acid, ethyl ester
33	50.026	0.09	1,3-Cyclohexadiene-l-carboxaldehyde, 2,6,6-trimethyl-
34	50.790	0.07	2-Furanmethanol
35	51.174	0.22	Benzoic acid, ethyl ester
36	51.596	0.23	Cyclooctasiloxane, hexadecamethyl-
37	51.788	0.32	Butanedioic acid, diethyl ester
38	57.162	0.09	Benzoic acid, 2-hydroxy-, methyl ester
39	57.807	0.37	Acetic acid, phenyl-, ethyl ester
40	58.906	0.10	Dodecanoic acid, methyl ester
41	59.428	0.07	B-Phenylethyl acetate
42	59.675	0.24	Silanediol, dimethyl-
43	60.344	0.03	Cyclononasiloxane, octadecamethyl-
44	61.125	0.88	Dodecanoic acid, ethyl ester

45	61.595	0.10	5,9-Undecadien-2-one, 6,10-dimethyl-, (E)-
46	63.092	1.19	Benzenepropanoic acid, ethyl ester
47	64.419	1.02	Benzeneethanol
48	65.293	0.09	Benzeneacetaldehyde, .alpha.-ethylidene-
49	65.950	0.08	3-Buten-2-one, 4-(2,6,6-trimethyl-l-cyclohexen-l-yl) -
50	67.340	0.07	Ethanone, l-(lH-pyrrol-2-yl)-
51	69.541	0.16	Myristic acid, methyl ester
52	70.229	1.40	Benzeneethanol, 2-methoxy-
53	71.479	2.96	Tetradecanoic acid, ethyl ester
54	72.435	0.05	5-Methyl-2-phenyl-2-hexenal
55	74.045	0.09	Pentadecanoic acid, ethyl ester
56	74.160	1.11	1,4,7,10,13,16-Hexaoxacyclooctadecane
57	74.751	0.05	Ethyl 13-methyl-tetradecanoate
58	76.146	0.06	Diallylvinylmethylsilane
59	76.327	0.10	Pentadecanoic acid, ethyl ester
60	77.056	0.10	Phenol, 4-ethyl-
61	77.934	0.08	2-Methoxy-5-vinylphenol
62	79.013	0.08	(E)-l-Methyl-3-(prop-l-en-l-yl)trisulfane
63	79.279	0.28	Hexadecanoic acid, methyl ester
64	80.468	0.14	Octaethylene glycol monododecyl ether
65	81.015	19.91	Hexadecanoic acid, ethyl ester
66	82.121	0.45	Ethyl 9-hexadecenoate
67	84.574	0.12	2(4H)-Benzofuranone, 5,6,7,7a-tetrahydro-4,4,7a-trimethyl-, (R)-
68	89.082	0.24	9-Octadecenoic acid (Z)-, methyl ester

69	89.731	0.71	Octadecanoic acid, ethyl ester
70	90.527	5.92	Ethyl oleate
71	90.860	0.55	(E)-9-Octadecenoic acid ethyl ester
72	91.041	0.94	9,12-Octadecadienoic acid (Z,Z)-,methyl ester
73	92.445	15.75	Linoleic acid ethyl ester
74	94.471	2.10	9,12,15-Octadecatrienoic acid, ethyl ester, (Z,Z,Z)-
75	99.651	0.13	3,6,9,12,15-Pentaoxanonadecan-1-ol
			**Go-34**
1	4.409	1.58	2-Methoxy-N-methylethylamine
2	7.672	4.84	Ethyl acetate
3	9.109	26.49	Ethanol
4	11.982	0.40	Acetic acid, 2-methyl-propyl este
5	15.261	0.08	1,5-Pentanediol
6	15.954	0.67	1-Propanol, 2-methyl-
7	17.607	9.01	1-Butanol, 3-methyl, acetate
8	18.527	0.08	1,4-Dimethylbenzene
9	19.919	0.18	Cyclopentasiloxane, decamethyl-
10	22.880	2.89	1-Butanol, 3-methyl
11	24.249	0.12	Furan, 2-pentyl-
12	24.485	0.05	Ethyl hezanoate
13	26.960	0.08	Acetic acid, hexyl ester
14	30.136	0.17	Heptenal
15	31.121	1.39	Cyclopentasiloxane, decamethyl-
16	31.384	0.29	Ethyl 2-hydrozypropanoate

17	32.273	0.07	Formic acid, hexyl ester
18	32.467	0.23	Tridecane, 2-methyl-
19	32.742	0.06	4-Aminopyrimidine
20	33.698	0.11	1-Propanol, 3-ethoxy-
21	34.716	0.13	Nonanal
22	35.264	0.13	Tetradecane
23	35.486	0.10	Pyrazine, trimethyl-
24	36.926	0.11	3-Dodecen-1-al
25	37.444	0.17	Octanoic acid, ethyl ester
26	38.812	5.23	2-Furan-carboxaldehyde
27	39.480	1.17	Acetic acid
28	40.000	1.14	Pyrazine, tetramethyl-
29	41.443	0.23	(3E, 5E)-Hepta-3,5-dien-2-one
30	41.536	0.20	Pentadecane
31	47.798	0.94	Tetradecamethylcycloheptasiloxane
32	42.526	0.86	Benzaldehyde
33	43.456	0.50	2-Furanmethaol, acetate
34	44.366	0.60	1,6-Octadien-3-ol, 3,7-dimethyl-
35	44.924	0.15	Hentriacontane
36	45.567	0.20	1H-Pyrazole, 1,3,5-trimethyl-
37	46.062	0.86	2,3-Butanediol
38	46.891	0.23	5-Hepten-2-one, 3-methylene-
39	47.549	0.15	Hexadecane
40	48.049	0.10	2,3-Epoxy-1-(methoxymethoxy)gerani

41	48.535	0.07	Bezaldehyde, 2-methyl-
42	48.627	0.16	1-Cyclohezene-1-carboxaldehyde, 2,6,6-trimethyl
43	49.617	0.20	Benzeneacetaldehyde
44	49.655	0.08	Phenyl acetaldehyde
45	49.788	0.37	Pentadecanoic acid, ethyl ester
46	50.012	0.32	Oxirane, [(phenylmethoxy)methyl]-
47	50.274	0.40	Silanediol, dimethyl-
48	50.788	0.79	2-Furanmethanol
49	51.183	0.15	Pyrimidine, 4-amino-2-methoxy-
50	51.604	0.07	Cyclooctasiloxane, hexadecamethyl-
51	52.935	0.11	5-Hydroxy-6-methoxy-8-[(4-amino-1-methylbutyl)amino]quinoline trihydrobromide
52	53.122	0.21	.Beta. Fenchyl alcohol
53	53.316	0.15	1,1,7-Trimethyl-4-methylenedecahydro-1H-cyclopropa[E]azulene
54	54.086	1.19	Ethanol, 2-bromo-
55	54.384	2.18	1,3-Cyclohezadiene, 5-(1,5-dimethy 1-4-hexenyl)-2-methyl-, [S-(R*, S*)]-
56	54.644	1.65	Acetic acid, phenylmethyl ester
57	57.161	0.75	Benzoic acid, 2-hydroxy-, methyl ester
58	58.920	0.22	Ethyl 4-hydroxybutanoate
59	59.156	0.18	2,4-Decadienal, (E,E)-
60	59.419	10.84	Acetic acid, 2-phenylethyl ester
61	59.690	0.10	Silanediol, dimethyl-
62	61.124	0.16	Dodecanoic acid, ethyl ester
63	61.599	0.11	2-Cyclopenten-1-one, 2,3,4-trimethyl-
64	62.597	0.55	Benzenemethanol

65	63.041	0.13	Propanoic acid, 2-phenylethyl este.beta.-Phenylethyl butyrate
66	64.419	9.45	Benzeneethanol
67	65.947	0.16	(3E)-4-(2,6,6-Trimethyl-1-cyclohexen-1-yl)-3-buten-2-one
68	66.800	0.19	4H-Pyran-4-one, 3-hydroxy-2, 6-dimethyl-
69	67.340	0.94	Ethanone, 1-(1H-pyrrol-2-yl)-
70	67.957	0.06	4H-Pyran-4-one, 3-hydroxy-2-dimethyl-
71	68.978	0.15	Phenol
72	70.220	0.36	Gamma, gamma-dimethylallenty-1-butynyl sulfaide
73	70.399	0.15	Octyldiglycol
74	70.953	0.07	3,7,11-Trimethyl-3-hydroxy-6, 10-dodecadien-1-yl acetate
75	71.475	0.13	Tetradecanoic acid, ethyl ester
76	76.341	0.08	Majapol D
77	77.423	0.36	15-Crown-5
78	77.930	0.63	2-Methoxy-4-vinylphenol
79	80.971	1.00	Hexadecanoic acid, methyl ester
80	84.575	0.24	2(4H)-Benzofuranone, 5,6,7,7a-tetrahydro-4,4,7a-trimethyl-, (r)-
81	86.472	2.06	1,4,7,10,13,16-Hexaoxacycloocatadecane
82	96.966	0.41	21-Krone-7
			**Go-35**
1	7.672	0.73	Acetic acid, ethyl ester
2	7.948	0.09	1-Amino-2-butanol
3	8.487	0.05	Butanal, 3-methyl-
4	9.120	14.01	Ethanol
5	15.894	0.19	1-Propanol, 2-methyl-

6	17.625	0.04	(l-Hydroxyethylidene)malonic acid diethyl ester
7	19.925	0.05	Cyclopentasiloxane, decamethyl-
8	22.869	0.61	1-Butanol, 3-methyl-
9	24.486	0.15	Hexanoic acid, ethyl ester
10	30.150	0.03	Ethane, 1,1-diethoxy-
11	30.954	0.02	Heptanoic acid, ethyl ester
12	31.123	0.37	Cyclohexasiloxane, dodecamethyl-
13	31.381	0.07	Ethyl 2-hydroxypropanoate
14	32.468	0.04	1-Decanol, 2,2-dimethyl-
15	32.746	0.04	3-Furanacetic acid, .alpha.-oxo-
16	35.261	0.03	Tetradecane, 2,6,10-trimethyl-
17	35.483	0.04	Pyrazine, trimethyl-
18	37.447	0.32	Octanoic acid, ethyl ester
19	38.812	1.65	Furfural
20	39.256	0.03	5-Hepten-2-ol, 6-methyl-
21	39.995	0.29	2,3,5,6 Tetramethyl pyrazine
22	40.882	0.08	D-(+)-Xylose, tetramethyl ether
23	41.445	0.12	Ethanone, l-(2-furanyl)-
24	41.798	0.31	Cycloheptasiloxane, tetradecamethyl-
25	42.523	0.22	Benzaldehyde
26	43.743	0.05	Nonanoic acid, ethyl ester
27	43.863	0.09	2,3-Butanediol
28	44.064	0.05	Propane, 1,1'-[ethylidenebis(oxy)]bis-
29	44.366	0.09	l,6-Octadien-3-ol, 3,7-dimethyl-

30	44.928	0.02	Heneicosane
31	45.554	0.15	2-Furancarboxaldehyde, 5-methyl-
32	45.789	0.03	1-Propanone, 1-(2-furanyl)-
33	46.058	0.20	2,3-Butanediol
34	47.014	0.12	Dodecanoic acid, ethyl ester
35	47.542	0.05	(lR*,4R/S*,5S*)-4,5-Dimethyl-9-oxabicyclo[3.3.1]nonan-1-ol
36	48.059	0.23	Neopentylidenecyclohexane
37	48.537	0.06	Benzoic acid, methyl ester
38	48.623	0.05	1-Cyclohexene-l-carboxaldehyde, 2,6,6-trimethyl-
39	49.664	0.10	Benzeneacetaldehyde
40	49.800	0.22	Decanoic acid, ethyl ester
41	50.022	0.11	1,3-Cyclohexadiene-l-carboxaldehyde, 2,6,6-trimethyl-
42	50.783	0.32	2-Furanmethanol
43	51.180	0.20	Benzoic acid, ethyl ester
44	51.600	0.02	Cystine, TBS 2X
45	51.791	0.14	Butanedioic acid, diethyl ester
46	52.599	0.04	2,6,6-Trimethyl-2-cyclohexene-l,4-dione
47	52.935	0.06	5-Hydroxy-6-methoxy-8-[(4-amino-l-methylbutyl)amino]quinoline trihydrobromide
48	53.116	0.07	Cyclofenchene
49	57.164	0.11	Benzoic acid, 2-hydroxy-, methyl ester
50	57.806	0.06	Acetic acid, phenyl-, ethyl ester
51	58.343	0.08	Ethanone, l-(2-furanyl)-
52	58.902	0.05	Dodecanoic acid, methyl ester
53	59.420	0.20	Acetic acid, 2-phenylethyl ester

54	59.704	0.04	Silanediol, dimethyl-
55	61.128	1.39	Dodecanoic acidm ethyl ester
56	61.602	0.07	l,l-Dimethyl-l-silacyclo-2,4-hexadiene
57	64.416	1.19	Benzeneethanol
58	65.283	0.08	Benzeneacetaldehyde, .alpha.-ethylidene-
59	65.951	0.10	3-Buten-2-one, 4-(2,6,6-trimethyl-1-cyclohexen-l-yl)-
60	66.796	0.08	2-Methyl-3-methoxy-4H-pyran-4-one
61	67.341	0.17	Ethanone, 1- (lH-pyrrol-2-yl)-
62	69.54	0.10	Myristic acid, methyl ester
63	70.216	0.05	1,3-Butadiene, l-(ethylthio)-
64	70.963	0.05	(6E)-3-Hydroxy-3,7,ll-trimethyl-6,10-dodecadienyl acetate
65	71.477	3.22	Tetradecanoic acid, ethyl ester
66	73.636	0.06	Nicotinyl alcohol
67	74.035	0.32	Ethyl 13-methyl-tetradecanoate
68	74.145	0.05	Diethyl suberate
69	74.753	0.04	Tetradecanoic acid, 2-methyl-, methyl ester
70	76.144	0.16	Furan, 2-(l,2-dimethoxyethyl)-
71	76.325	0.14	Pentadecanoic acid, ethyl ester
72	77.344	0.11	15-Crown-5
73	77.934	0.10	2-Methoxy-4-vinylphenol
74	79.015	0.16	Diethyl azelate
75	79.275	1.10	Hexadecanoic acid, methyl ester
76	81.022	25.09	Hexadecanoic acid, ethyl ester
77	82.119	1.11	Ethyl 9-hexadecenoate

78	84.568	0.19	2(4H)-Benzofuranone, 5,6,7,7a-tetrahydro-4,4,7a-trimethyl-, (R)-
79	85.427	0.08	Heptadecanoic acid, ethyl ester
80	89.077	0.42	9-Octadecenoic acid, methyl ester, (E)-
81	89.728	1.02	Octadecanoic acid, ethyl ester
82	90.525	6.98	Ethyl (9Z)-9-octadecenoate
83	90.859	0.88	(E)-9-Octadecenoic acid ethyl ester
84	91.040	1.46	9,12-Octadecadienoic acid (Z,Z)-,methyl ester
85	92.462	25.58	Linoleic acid ethyl ester
86	94.470	4.16	9,12,15-Octadecatrienoic acid, ethyl ester, (Z,Z,Z)-
87	96.971	1.76	1,4,7,10,13,16-Hexaoxacyclooctadecane
